# Supplementary material for: Deep learning using electroencephalogram (EEG) data for diagnosing and predicting SSRI response in major depressive disorder
Source: Commun Med (Lond). 2026 Mar 23;6:159. doi: 10.1038/s43856-026-01394-z (PMC13009148; doi:10.1038/s43856-026-01394-z)
Supplement: Supplementary file 2 — Supplementary Information [file 43856_2026_1394_MOESM2_ESM.pdf]

# Supplementary Methods and Results

## 1.1 Channel number and names for the different recording sites

Leipzig I: Number of channels in selected files: 27

Channels: ['C3', 'C4', 'CP1', 'CP2', 'CP5', 'CP6', 'F3', 'F4', 'F7', 'F8', 'FC1', 'FC2', 'FC5', 'FC6', 'Fp1', 'Fp2', 'O1', 'O2', 'Oz', 'P3', 'P4', 'P7', 'P8', 'T7', 'T8', 'TP10', 'TP9']

Leipzig II: Number of channels in selected files: 31

Channels: ['C3', 'C4', 'CP1', 'CP2', 'CP5', 'CP6', 'Cz', 'F3', 'F4', 'F7', 'F8', 'FC1', 'FC2', 'FC5', 'FC6', 'Fp1', 'Fp2', 'Fz', 'O1', 'O2', 'Oz', 'P3', 'P4', 'P7', 'P8', 'POz', 'Pz', 'T7', 'T8', 'TP10', 'TP9']

Prague I: Number of channels in selected files: 19

Channels: ['C3', 'C4', 'Cz', 'F3', 'F4', 'F7', 'F8', 'FP1', 'FP2', 'Fz', 'O1', 'O2', 'P3', 'P4', 'Pz', 'T3', 'T4', 'T5', 'T6']

Prague II: Number of channels in selected files: 19

Channels: ['C3', 'C4', 'Cz', 'F3', 'F4', 'F7', 'F8', 'FP1', 'FP2', 'Fz', 'O1', 'O2', 'P3', 'P4', 'Pz', 'T3', 'T4', 'T5', 'T6']

Canada I: Number of channels in selected files: 32

Channels: ['C3', 'C4', 'CP1', 'CP2', 'CP5', 'CP6', 'Cz', 'F3', 'F4', 'F7', 'F8', 'FC1', 'FC2', 'FC5', 'FC6', 'FT10', 'FT9', 'Fp1', 'Fp2', 'Fz', 'O1', 'O2', 'Oz', 'P3', 'P4', 'P7', 'P8', 'Pz', 'T7', 'T8', 'TP10', 'TP9']

CANBIND: Number of channels in selected files: 58

Channels: ['AF3', 'AF4', 'C1', 'C2', 'C3', 'C4', 'C5', 'C6', 'CP1', 'CP2', 'CP3', 'CP4', 'CP5', 'CP6', 'CPz', 'Cz', 'F1', 'F2', 'F3', 'F4', 'F5', 'F6', 'F7', 'F8', 'FC1', 'FC2', 'FC3', 'FC4', 'FC5', 'FC6', 'FCz', 'FP1', 'FP2', 'FPz', 'FT7', 'FT8', 'Fz', 'O1', 'O2', 'Oz', 'P1', 'P2', 'P3', 'P4', 'P5', 'P6', 'P7', 'P8', 'PO3', 'PO4', 'PO7', 'PO8', 'POz', 'Pz', 'T7', 'T8', 'TP7', 'TP8']

## 1.2 Results of classification using only two frontal electrodes F3 and F4

Classification of HC versus MDD: Test accuracy for all pooled segments of unseen subjects from the test set was 52.4% in total (not subject-wise) and the percentage of correctly classified unseen subjects based on the number of correct classified segments of a subject was 53.2% with a standard deviation of 41.4% across subjects.

Classification of Responders versus Non-Responders: Test accuracy for all pooled segments of unseen subjects from the test set was 54.1% in total (not subject-wise) and the percentage of correctly classified unseen subjects based on the number of correct classified segments of a subject was 53.1% with a standard deviation of 39.2% across subjects.

## 1.3 Machine Learning Algorithms and results of ML classification

1. AdaBoost (Adaptive Boosting)

- Type: Ensemble learning (Boosting)
- Features: Iteratively improves weak classifiers; sensitive to noise; works well with structured data but prone to overfitting on noisy datasets.

2. Decision Tree

- Type: Tree-based model
- Features: Simple, interpretable; prone to overfitting; splits data hierarchically for classification.

3. Gradient Boosting

- Type: Ensemble learning (Boosting)
- Features: Strong learner built from weak classifiers; powerful but computationally intensive; good for capturing complex patterns.

4. K-Nearest Neighbors (KNN)

- Type: Distance-based (Instance-based learning)
- Features: Non-parametric; classifies based on proximity to neighboring data points; sensitive to feature scaling and high-dimensional data.

5. LightGBM

- Type: Gradient boosting framework
- Features: Optimized for large datasets; computationally efficient; fast training while maintaining high accuracy.

6. Logistic Regression

- Type: Statistical model
- Features: Linear classifier; probabilistic output; interpretable; works well for simple decision boundaries.

7. Naïve Bayes

- Type: Probabilistic classifier
- Features: Assumes feature independence; computationally efficient; effective in text and medical classification tasks.

8. Random Forest

- Type: Ensemble learning (Bagging)
- Features: Multiple decision trees reduce overfitting; robust and generalizable; handles non-linearity well.

9. Support Vector Machine (SVM)

- Type: Margin-based classifier
- Features: Finds the optimal hyperplane for classification; effective in high-dimensional spaces; supports non-linear classification via kernels.

10. XGBoost (Extreme Gradient Boosting)

- Type: Optimized gradient boosting
- Features: Fast training; built-in regularization to prevent overfitting; widely used in competition-grade ML applications.

Results of classification

| **Algorithm** | **MDD vs HC [%]** | **Responders vs Non-Responders [%]** |
|---|---|---|
| AdaBoost | 0.5685 | 0.6029 |
| Decision Tree | 0.5308 | 0.4803 |
| Gradient Boosting | 0.5753 | 0.4705 |
| KNN | 0.6164 | 0.5 |
| LightGBM | 0.6404 | 0.4509 |
| Logistic Regression | 0.5685 | 0.5245 |
| Naive Bayes | 0.5205 | 0.4852 |
| Random Forest | 0.6609 | 0.446 |
| SVM | 0.589 | 0.5245 |
| XGBoost | 0.6061 | 0.4313 |

Table 1: Used Machine Learning Algorithms and corresponding results (Major Depressive Disorder = MDD; Healthy Control = HC, K-Nearest Neighbors=KNN, Light Gradient Boosting Machine=LightGBM, Support Vector Machine = SVM, Extreme Gradient Boosing = XGBoost)

## 1.4 Description of EEGNet and parameters used

1. Temporal Convolution Block (Learning Frequency Filters)

- Conv2D (1,64, 16 filters, no bias, same padding): Applies temporal convolutions across the EEG time dimension to learn frequency-specific features.
- Batch Normalization: Normalizes the activations, improving stability and convergence.

2. Depthwise Convolution Block (Learning Spatial Features)

- DepthwiseConv2D (10,1, depth multiplier=2): Applies spatial convolutions, learning EEG channel-wise dependencies while keeping the number of parameters low.
- Batch Normalization: Normalizes feature maps.
- Activation (ELU): Adds non-linearity to improve feature extraction.
- Average Pooling (1,4): Reduces spatial dimensions, retaining key information.
- Dropout (0.5): Prevents overfitting by randomly deactivating neurons.

3. Separable Convolution Block (Time-Frequency Refinement)

- SeparableConv2D (1,16, 32 filters, same padding): Further refines extracted features by applying pointwise and depthwise convolutions.
- Batch Normalization: Maintains stable activations.

- Activation (ELU): Adds non-linearity.
- Average Pooling (1,8): Further reduces dimensions for efficiency.
- Dropout (0.5): Additional regularization.

4. Fully Connected Classification Layers

- Flatten: Converts feature maps into a vector for classification.
- Dense (64 Neurons, ELU): A fully connected layer for feature representation.
- Dropout (0.5): Further reduces overfitting.
- Dense (2 Neurons, Softmax Output): The final layer classifying EEG data into two categories.

Model Summary

- Total Parameters: 34,114
- Trainable Parameters: 33,954
- Non-trainable Parameters: 160

| Layer (type) | Output Shape | Param # |
|---|---|---|
| Conv2D | (None, 10, 500, 16) | 1024 |
| BatchNormalization | (None, 10, 500, 16) | 64 |
| DepthwiseConv2D | (None, 1, 500, 32) | 320 |
| BatchNormalization | (None, 1, 500, 32) | 128 |
| Activation | (None, 1, 500, 32) | 0 |
| AveragePooling2D | (None, 1, 125, 32) | 0 |
| Dropout | (None, 1, 125, 32) | 0 |
| SeparableConv2D | (None, 1, 125, 32) | 1536 |
| BatchNormalization | (None, 1, 125, 32) | 128 |
| Activation | (None, 1, 125, 32) | 0 |
| AveragePooling2D | (None, 1, 15, 32) | 0 |
| Dropout | (None, 1, 15, 32) | 0 |
| Flatten | (None, 480) | 0 |
| Dense | (None, 64) | 30784 |
| Dropout | (None, 64) | 0 |
| Dense | (None, 2) | 130 |
| Total Parameters | | 34114 |
| Trainable Parameters | | 33954 |
| Non-trainable Parameters | | 160 |

Table 2: Architecture of the EEGNet model (Convolutional 2 Dimensional = Conv2D)

# 1.5 Results of the EEGnet classification

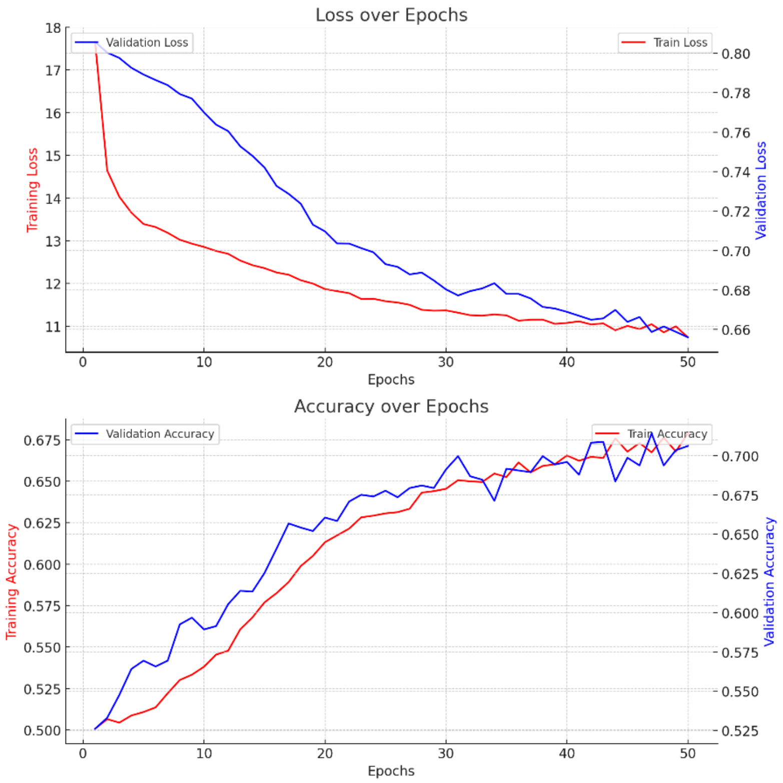


Figure 1: Results of the EEGNet with training and validation losses (top tile) and accuracies (bottom tile) (note that the results have been truncated at epoch 50)

# 1.6 Hyperparameter Tuning

Details of the Training Process:

1. Number of Epochs Trained:

Each model configuration in the random search was trained for 50 epochs.

2. Number of Hyperparameter Configurations Checked:

The RandomizedSearchCV was set to explore 10 different hyperparameter combinations.

The grid included variations in:

- Number of convolutional filters: [100, 200, 300]
- Dense layer size: [2000, 5000, 7000]
- Dropout rate: [0.3, 0.5]
- Learning rate: [0.00005, 0.0001, 0.0005]

3. Data Used for Training and Evaluation:

- During hyperparameter tuning, cross-validation (CV) was performed with a 3-fold split of the training data.
- After selecting the best model, it was evaluated on the test set.

## 1.7 Recording Site Info

| EEG Recording Details | | | | | | | | | |
|---|---|---|---|---|---|---|---|---|---|
| | CANBIND | | | | Canada 1 | Leipzig 1 | Leipzig 2 | Prague 1 | Prague 2 |
| | CAMH | TGH/TWH | UBC | QNS | | | | | |
| Model of EEG recording system: | Compumedics Neuroscan Synamp | Biosemi Active-Two amplifier system | QuickAmp amplifier | EGI NetAmps 300 | QuickAmp amplifier | QuickAmp amplifier | BrainAmp amplifier | BrainScope amplifier | BrainScope amplifier |
| Number of electrodes: | 64 + 4 EOG | 64 + 4 EOG | 64 + 4 EOG | 128 | 64 + 4 EOG | 27 + 2 EOG | 31 + 2 EOG | 19 + 2 EOG | 19 + 2 EOG |
| Montage: | 10-10 | 10-10 | 10-10 | HydroCel GSN 128 | 10-10 | 10-10 | 10-10 | 10-20 | 10-20 |
| Electrode material: | Ag/AgCl | Ag/AgCl | Ag/AgCl | Silver chloride plated carbon-fiber pellet | Ag/AgCl | Ag/AgCl | Ag/AgCl | Ag/AgCl | Ag/AgCl |
| Conductive material: | Conductive gel | Conductive gel | Quik Gel Conductive gel | Potassium Chloride solution | Conductive gel | Quik Gel Conductive gel | Conductive gel | Conductive gel | Conductive gel |
| Reference electrode: | Posterior to Cz | CMS/DRL (Common Mode Sense/Driven Right Leg) | Cz | Cz | Av | Av | Cz | Cz | Cz |
| Sampling rate: | 1000 Hz | 512 Hz | 1000 Hz | 1000 Hz | 512 Hz | 1000 Hz | 1000 Hz | 250 Hz | 1000 Hz |
| Analog filters: | (none) | Anti-aliasing first-order filter, -3dB at 3.6 kHz | -unknown- | 6 kHz anti-aliasing filter | -unknown- | (none) | (none) | -unknown- | -unknown- |
| Digital filters: | High-pass filter 0.05 Hz, low-pass filter 100 Hz | Low-pass 5th order sinc filter, -3dB at 20% of sampling frequency | High-pass filter 0.01 Hz, low-pass filter 499 Hz | 4 kHz anti-aliasing filter, 0.04 Hz first order high-pass filter, 100 Hz FIR low-pass filter | High-pass filter 0.01 Hz, low-pass filter 80 Hz | High-pass filter 0.01 Hz, low-pass filter 70 Hz | High-pass filter 0.01 Hz, low-pass filter 70 Hz | High-pass filter 0.5 Hz, low-pass filter 70 Hz | High-pass filter 0.5 Hz, low-pass filter 70 Hz |

Table 3: Detailed description of the used technical equipment of the different recording sites

(CAMH/TGH/TWH/UBC/QNS = recoding sites, electrooculogram = EOG, Ag/AgCl = Silver/SilverChloride)

## 1.8 Sociodemographic Info

| | CANBIND | | | | Ottawa | Leipzig 1 | Leipzig 2 | Prague 1 | Prague 2 |
|---|---|---|---|---|---|---|---|---|---|
| | CAMH | TGH/TWH | UBC | QNS | | | | | |
| Age [years (SD)] | 30.4 (13.0) | 42.7 (14.4) | 35.8 (13.0) | 35.6 (12.0) | 42.5 (10.4) | 39.5 (11.8) | 36.5 (8.1) | 47.5 (8.7) | 45.6 (8.6) |
| Gender (Female [%]) | 100.0 | 50.0 | 58.7 | 64.8 | 63.1 | 64.1 | 60.8 | 76.2 | 76.5 |

Table 4: Detailed description of the age and sex of participants from the different recording sites

(CAMH/TGH/TWH/UBC/QNS = recoding sites, Standard Deviation = SD)

## 1.9 Site Specific Accuracies

| | CANBIND | Ottawa | Leipzig 1 | Leipzig 2 | Prague 1 | Prague 2 |
|---|---|---|---|---|---|---|
| HC versus MDD | 73.4% | 63.5% | 63.2% | 60.1% | 61.0% | 61.0% |

| | | | | | | |
|---|---|---|---|---|---|---|
| Responder versus Non Responder | 81.1% | 75.0% | 82.2% | 85.4% | 78.3% | 78.3% |

Table 5: Site Specific Accuracies (Major Depressive Disorder = MDD, Healthy Control = HC)

## 1.10 ROC curves and AUC values for different DL models

To make the results more interpretable, we calculated the Receiver-Operator-Characteristics (ROC) curves and Area-under-the-Curve (AUC) values for models with increasing accuracies for differentiation of Responders and Non-Responders are given below. Due to a lack of different thresholds within the same model, the results should be interpreted with caution.

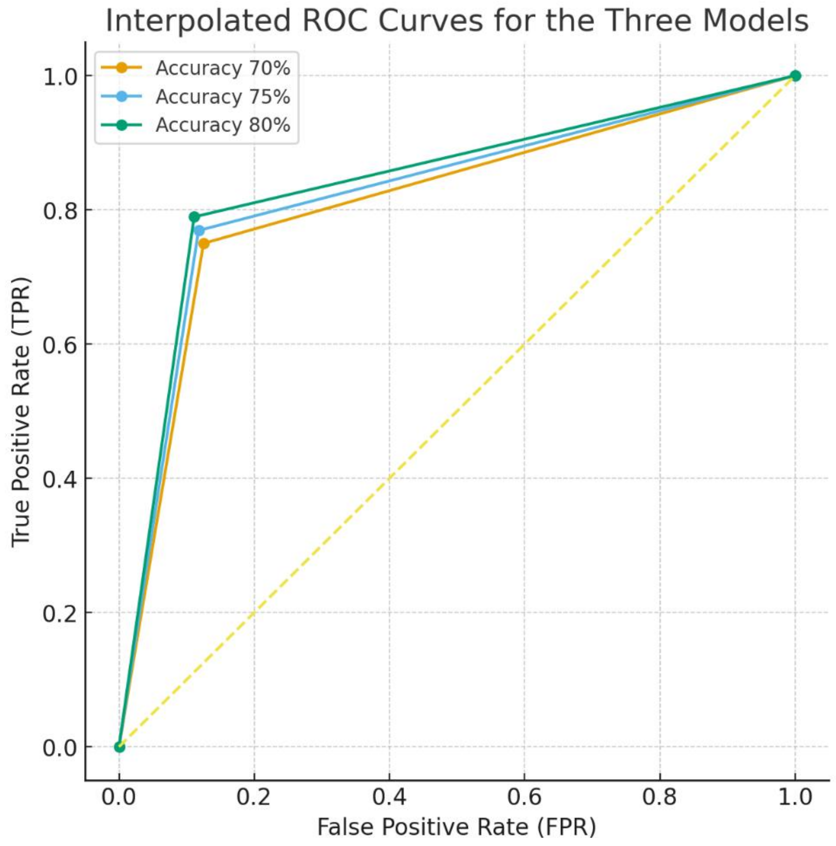


Figure 2: Receiver-Operator Characteristics (ROC) for the used models

| *Model* | *AUC* |
|---|---|
| *Accuracy 70%* | *0.8125* |
| *Accuracy 75%* | *0.8258* |
| *Accuracy 80%* | *0.8392* |

Table 6: Corresponding area-under-the-curve (AUC) estimations

## 1.11 Usage of 30Hz low-pass filter

Since the frequency range between 30 and 45Hz is prone to sources from muscle activity, we repeated the main analyses of this work after the application of a more rigorous low-pass filter at

30Hz and then repeated the training procedure for the classification tasks (MDD versus MC and Responders versus Non-Responders).

Classification of MDD subjects versus HC subjects from all datasets showed a train accuracy of 70.92% (compared to 73.16% with a 45Hz filter) and a validation accuracy of 68.0% (compared to 71.13% with a 45Hz filter). Test accuracy for all pooled segments of unseen subjects from the test set was 63.44% (compared to 64.84% with a 45Hz filter) in total (not subject-wise) and the percentage of correctly classified unseen subjects based on the number of correct classified segments of a subject was 65% (compared to 67.5% with 45Hz filter).

Classification of responders versus non-responders of SSRI treatment showed 69.94% (versus 71.8% for 45Hz filter) accuracy for single segments for the training set after 708 training epochs. Validation set accuracy was 65.42% (versus 66.4% in the 45Hz filter model). The test set reached 59% (64% for 45Hz filter) for pooled segments and a mean value of 55% (against 79% for 45Hz filter) for correct subject-wise classification.

Thus, all results were slightly worse when applying a low-pass filter of 30Hz instead of 45Hz. Although it cannot be ruled out that muscle activity in the range from 30-45Hz contributes to the correct labelling, another explanation would be that EEG-gamma activity in this range yields important information for a correct classification. Since answering this question was beyond the scope of the presented work, further studies on this topic seem necessary.

## 1.12 Differences of eye-related movements in the EEG data across the groups

To determine whether the number of eye-derived artefacts was different between the tested groups and thus may have contributed to the classification results, additional analysis was performed. Eye blinks were automatically identified using the MNE-Python function find_eog_events. The EEG was band-pass filtered between 1 and 8 Hz to isolate the slow ocular deflections characteristic of blinks. A bipolar channel based on electrooculogram EOG) data or reconstructed EOG channels (F7-F8) served as an electrooculographic (EOG) surrogate. Peaks exceeding an amplitude threshold of 150 µV were classified as blink events and used for subsequent quantification (blinks per minute). The amount of eye-related movement events per minute for all groups (HC versus MDD and Responders versus Non-Responders) were then tested against each other using a t-test. No differences between MDD and HC were found nor between Responders and Non-Responders.

| Group | Mean | SD (%) |
|---|---|---|
| HC | 0.26 | 1.12% |
| MDD | 0.35 | 1.47% |
| Responders | 0.40 | 1.91% |
| Non-Responders | 0.33 | 0.99% |

Table 7: Given are the mean numbers of eye-movement events per minute, standard deviation for the different groups (Major Depressive Disorder = MDD, Healthy Control = HC, Standard Deviation = SD)

| Comparison | t-value | p-value |
|---|---|---|
| HC vs MDD | -0.69 | 0.49 |
| Responders vs Non-Responders | 0.34 | 0.74 |

Table 8: Results of the comparison between groups for eye-movement related events across the different labelling groups. There were no significant differences between MDD and HC and between Responders and Non-Responders (Major Depressive Disorder = MDD, Healthy Control = HC)
