# Supplementary material for: Deep learning using electroencephalogram (EEG) data for diagnosing and predicting SSRI response in major depressive disorder
Source: Commun Med (Lond). 2026 Mar 23;6:159. doi: 10.1038/s43856-026-01394-z (PMC13009148; doi:10.1038/s43856-026-01394-z)
Supplement: Supplementary file 3 — Description for supplementary data files [file 43856_2026_1394_MOESM3_ESM.docx]

Description for supplementary data files

1. Filename „Supplementary Data 1“: Raw values for generating the accuracy and loss-plots of Figure 2.
2. Filename „Supplementary Data 2“: Data for generating topoplots of Figure 2 and 4 with electrode names, values for the topoplots and codes for generating.
3. Filename „Supplementary Data 3“: Raw values for generating the accuracy and loss-plots of Figure 4.
4. Filename „Supplementary Data 4“: Raw values for generating overlay plot of Figure 5
