# Supplementary material for: Deep learning using electroencephalogram (EEG) data for diagnosing and predicting SSRI response in major depressive disorder
Source: Commun Med (Lond). 2026 Mar 23;6:159. doi: 10.1038/s43856-026-01394-z (PMC13009148; doi:10.1038/s43856-026-01394-z)
Supplement: Supplementary file 4 — Supplementary Data 1 [file 43856_2026_1394_MOESM4_ESM.docx]

HC versus MDD

Total params: 4,536,302

Trainable params: 4,536,302

Non-trainable params: 0

_________________________________________________________________

Epoch 1/400

702/702 [==============================] - 6s 8ms/step - loss: 1.6636 - accuracy: 0.5729 - val_loss: 0.6946 - val_accuracy: 0.4226

Epoch 00001: val_loss improved from inf to 0.69456, saving model to path_to_save_best_model.h5

Epoch 2/400

702/702 [==============================] - 5s 7ms/step - loss: 1.6628 - accuracy: 0.5088 - val_loss: 0.6942 - val_accuracy: 0.4505

Epoch 00002: val_loss improved from 0.69456 to 0.69417, saving model to path_to_save_best_model.h5

Epoch 3/400

702/702 [==============================] - 5s 7ms/step - loss: 1.6630 - accuracy: 0.5055 - val_loss: 0.6929 - val_accuracy: 0.4867

Epoch 00003: val_loss improved from 0.69417 to 0.69294, saving model to path_to_save_best_model.h5

Epoch 4/400

702/702 [==============================] - 5s 7ms/step - loss: 1.6622 - accuracy: 0.5187 - val_loss: 0.6910 - val_accuracy: 0.5257

Epoch 00004: val_loss improved from 0.69294 to 0.69104, saving model to path_to_save_best_model.h5

Epoch 5/400

702/702 [==============================] - 5s 7ms/step - loss: 1.6605 - accuracy: 0.5131 - val_loss: 0.6871 - val_accuracy: 0.5436

Epoch 00005: val_loss improved from 0.69104 to 0.68707, saving model to path_to_save_best_model.h5

Epoch 6/400

702/702 [==============================] - 5s 7ms/step - loss: 1.6519 - accuracy: 0.5243 - val_loss: 0.6616 - val_accuracy: 0.6594

Epoch 00006: val_loss improved from 0.68707 to 0.66156, saving model to path_to_save_best_model.h5

Epoch 7/400

702/702 [==============================] - 5s 7ms/step - loss: 1.6257 - accuracy: 0.5750 - val_loss: 0.6334 - val_accuracy: 0.6722

Epoch 00007: val_loss improved from 0.66156 to 0.63336, saving model to path_to_save_best_model.h5

Epoch 8/400

702/702 [==============================] - 5s 7ms/step - loss: 1.5972 - accuracy: 0.6157 - val_loss: 0.6227 - val_accuracy: 0.6727

Epoch 00008: val_loss improved from 0.63336 to 0.62274, saving model to path_to_save_best_model.h5

Epoch 9/400

702/702 [==============================] - 5s 7ms/step - loss: 1.5675 - accuracy: 0.6342 - val_loss: 0.6223 - val_accuracy: 0.6830

Epoch 00009: val_loss improved from 0.62274 to 0.62228, saving model to path_to_save_best_model.h5

Epoch 10/400

702/702 [==============================] - 5s 7ms/step - loss: 1.5670 - accuracy: 0.6370 - val_loss: 0.6212 - val_accuracy: 0.6822

Epoch 00010: val_loss improved from 0.62228 to 0.62124, saving model to path_to_save_best_model.h5

Epoch 11/400

702/702 [==============================] - 5s 7ms/step - loss: 1.5603 - accuracy: 0.6446 - val_loss: 0.6173 - val_accuracy: 0.6825

Epoch 00011: val_loss improved from 0.62124 to 0.61733, saving model to path_to_save_best_model.h5

Epoch 12/400

702/702 [==============================] - 5s 7ms/step - loss: 1.5542 - accuracy: 0.6469 - val_loss: 0.6168 - val_accuracy: 0.6844

Epoch 00012: val_loss improved from 0.61733 to 0.61684, saving model to path_to_save_best_model.h5

Epoch 13/400

702/702 [==============================] - 5s 7ms/step - loss: 1.5482 - accuracy: 0.6534 - val_loss: 0.6318 - val_accuracy: 0.6697

Epoch 00013: val_loss did not improve from 0.61684

Epoch 14/400

702/702 [==============================] - 5s 7ms/step - loss: 1.5456 - accuracy: 0.6470 - val_loss: 0.6294 - val_accuracy: 0.6751

Epoch 00014: val_loss did not improve from 0.61684

Epoch 15/400

702/702 [==============================] - 5s 7ms/step - loss: 1.5380 - accuracy: 0.6551 - val_loss: 0.6218 - val_accuracy: 0.6814

Epoch 00015: val_loss did not improve from 0.61684

Epoch 16/400

702/702 [==============================] - 5s 7ms/step - loss: 1.5382 - accuracy: 0.6557 - val_loss: 0.6287 - val_accuracy: 0.6741

Epoch 00016: val_loss did not improve from 0.61684

Epoch 17/400

702/702 [==============================] - 5s 7ms/step - loss: 1.5321 - accuracy: 0.6569 - val_loss: 0.6298 - val_accuracy: 0.6714

Epoch 00017: val_loss did not improve from 0.61684

Epoch 18/400

702/702 [==============================] - 5s 7ms/step - loss: 1.5286 - accuracy: 0.6586 - val_loss: 0.6393 - val_accuracy: 0.6611

Epoch 00018: val_loss did not improve from 0.61684

Epoch 19/400

702/702 [==============================] - 5s 7ms/step - loss: 1.5347 - accuracy: 0.6577 - val_loss: 0.6343 - val_accuracy: 0.6670

Epoch 00019: val_loss did not improve from 0.61684

Epoch 20/400

702/702 [==============================] - 5s 7ms/step - loss: 1.5252 - accuracy: 0.6572 - val_loss: 0.6441 - val_accuracy: 0.6524

Epoch 00020: val_loss did not improve from 0.61684

Epoch 21/400

702/702 [==============================] - 5s 7ms/step - loss: 1.5218 - accuracy: 0.6620 - val_loss: 0.6318 - val_accuracy: 0.6670

Epoch 00021: val_loss did not improve from 0.61684

Epoch 22/400

702/702 [==============================] - 5s 7ms/step - loss: 1.5220 - accuracy: 0.6598 - val_loss: 0.6309 - val_accuracy: 0.6673

Epoch 00022: val_loss did not improve from 0.61684

Epoch 23/400

702/702 [==============================] - 5s 7ms/step - loss: 1.5248 - accuracy: 0.6582 - val_loss: 0.6369 - val_accuracy: 0.6613

Epoch 00023: val_loss did not improve from 0.61684

Epoch 24/400

702/702 [==============================] - 5s 7ms/step - loss: 1.5124 - accuracy: 0.6662 - val_loss: 0.6283 - val_accuracy: 0.6697

Epoch 00024: val_loss did not improve from 0.61684

Epoch 25/400

702/702 [==============================] - 5s 7ms/step - loss: 1.5174 - accuracy: 0.6619 - val_loss: 0.6311 - val_accuracy: 0.6659

Epoch 00025: val_loss did not improve from 0.61684

Epoch 26/400

702/702 [==============================] - 5s 7ms/step - loss: 1.5193 - accuracy: 0.6596 - val_loss: 0.6289 - val_accuracy: 0.6678

Epoch 00026: val_loss did not improve from 0.61684

Epoch 27/400

702/702 [==============================] - 5s 7ms/step - loss: 1.5160 - accuracy: 0.6622 - val_loss: 0.6335 - val_accuracy: 0.6624

Epoch 00027: val_loss did not improve from 0.61684

Epoch 28/400

702/702 [==============================] - 5s 7ms/step - loss: 1.5169 - accuracy: 0.6602 - val_loss: 0.6311 - val_accuracy: 0.6659

Epoch 00028: val_loss did not improve from 0.61684

Epoch 29/400

702/702 [==============================] - 5s 7ms/step - loss: 1.5096 - accuracy: 0.6613 - val_loss: 0.6354 - val_accuracy: 0.6600

Epoch 00029: val_loss did not improve from 0.61684

Epoch 30/400

702/702 [==============================] - 5s 7ms/step - loss: 1.5132 - accuracy: 0.6627 - val_loss: 0.6390 - val_accuracy: 0.6554

Epoch 00030: val_loss did not improve from 0.61684

Epoch 31/400

702/702 [==============================] - 5s 7ms/step - loss: 1.5122 - accuracy: 0.6627 - val_loss: 0.6280 - val_accuracy: 0.6676

Epoch 00031: val_loss did not improve from 0.61684

Epoch 32/400

702/702 [==============================] - 5s 7ms/step - loss: 1.5087 - accuracy: 0.6628 - val_loss: 0.6398 - val_accuracy: 0.6521

Epoch 00032: val_loss did not improve from 0.61684

Epoch 33/400

702/702 [==============================] - 5s 7ms/step - loss: 1.5112 - accuracy: 0.6597 - val_loss: 0.6327 - val_accuracy: 0.6605

Epoch 00033: val_loss did not improve from 0.61684

Epoch 34/400

702/702 [==============================] - 5s 7ms/step - loss: 1.5002 - accuracy: 0.6693 - val_loss: 0.6404 - val_accuracy: 0.6500

Epoch 00034: val_loss did not improve from 0.61684

Epoch 35/400

702/702 [==============================] - 5s 7ms/step - loss: 1.4948 - accuracy: 0.6682 - val_loss: 0.6364 - val_accuracy: 0.6562

Epoch 00035: val_loss did not improve from 0.61684

Epoch 36/400

702/702 [==============================] - 5s 7ms/step - loss: 1.4927 - accuracy: 0.6688 - val_loss: 0.6439 - val_accuracy: 0.6451

Epoch 00036: val_loss did not improve from 0.61684

Epoch 37/400

702/702 [==============================] - 5s 7ms/step - loss: 1.4974 - accuracy: 0.6674 - val_loss: 0.6380 - val_accuracy: 0.6554

Epoch 00037: val_loss did not improve from 0.61684

Epoch 38/400

702/702 [==============================] - 5s 7ms/step - loss: 1.4953 - accuracy: 0.6684 - val_loss: 0.6414 - val_accuracy: 0.6473

Epoch 00038: val_loss did not improve from 0.61684

Epoch 39/400

702/702 [==============================] - 5s 7ms/step - loss: 1.4985 - accuracy: 0.6631 - val_loss: 0.6323 - val_accuracy: 0.6638

Epoch 00039: val_loss did not improve from 0.61684

Epoch 40/400

702/702 [==============================] - 5s 7ms/step - loss: 1.4969 - accuracy: 0.6674 - val_loss: 0.6276 - val_accuracy: 0.6692

Epoch 00040: val_loss did not improve from 0.61684

Epoch 41/400

702/702 [==============================] - 5s 7ms/step - loss: 1.4852 - accuracy: 0.6722 - val_loss: 0.6485 - val_accuracy: 0.6424

Epoch 00041: val_loss did not improve from 0.61684

Epoch 42/400

702/702 [==============================] - 5s 7ms/step - loss: 1.4929 - accuracy: 0.6693 - val_loss: 0.6537 - val_accuracy: 0.6381

Epoch 00042: val_loss did not improve from 0.61684

Epoch 43/400

702/702 [==============================] - 5s 7ms/step - loss: 1.4919 - accuracy: 0.6686 - val_loss: 0.6330 - val_accuracy: 0.6624

Epoch 00043: val_loss did not improve from 0.61684

Epoch 44/400

702/702 [==============================] - 5s 7ms/step - loss: 1.4876 - accuracy: 0.6703 - val_loss: 0.6239 - val_accuracy: 0.6741

Epoch 00044: val_loss did not improve from 0.61684

Epoch 45/400

702/702 [==============================] - 5s 7ms/step - loss: 1.4885 - accuracy: 0.6733 - val_loss: 0.6388 - val_accuracy: 0.6535

Epoch 00045: val_loss did not improve from 0.61684

Epoch 46/400

702/702 [==============================] - 5s 7ms/step - loss: 1.4887 - accuracy: 0.6694 - val_loss: 0.6228 - val_accuracy: 0.6757

Epoch 00046: val_loss did not improve from 0.61684

Epoch 47/400

702/702 [==============================] - 5s 7ms/step - loss: 1.4858 - accuracy: 0.6686 - val_loss: 0.6403 - val_accuracy: 0.6527

Epoch 00047: val_loss did not improve from 0.61684

Epoch 48/400

702/702 [==============================] - 5s 7ms/step - loss: 1.4958 - accuracy: 0.6700 - val_loss: 0.6351 - val_accuracy: 0.6600

Epoch 00048: val_loss did not improve from 0.61684

Epoch 49/400

702/702 [==============================] - 5s 7ms/step - loss: 1.4840 - accuracy: 0.6738 - val_loss: 0.6316 - val_accuracy: 0.6619

Epoch 00049: val_loss did not improve from 0.61684

Epoch 50/400

702/702 [==============================] - 5s 7ms/step - loss: 1.4841 - accuracy: 0.6731 - val_loss: 0.6318 - val_accuracy: 0.6632

Epoch 00050: val_loss did not improve from 0.61684

Epoch 51/400

702/702 [==============================] - 5s 7ms/step - loss: 1.4817 - accuracy: 0.6771 - val_loss: 0.6550 - val_accuracy: 0.6410

Epoch 00051: val_loss did not improve from 0.61684

Epoch 52/400

702/702 [==============================] - 5s 7ms/step - loss: 1.4790 - accuracy: 0.6762 - val_loss: 0.6315 - val_accuracy: 0.6635

Epoch 00052: val_loss did not improve from 0.61684

Epoch 53/400

702/702 [==============================] - 5s 7ms/step - loss: 1.4822 - accuracy: 0.6732 - val_loss: 0.6391 - val_accuracy: 0.6548

Epoch 00053: val_loss did not improve from 0.61684

Epoch 54/400

702/702 [==============================] - 5s 7ms/step - loss: 1.4834 - accuracy: 0.6760 - val_loss: 0.6235 - val_accuracy: 0.6703

Epoch 00054: val_loss did not improve from 0.61684

Epoch 55/400

702/702 [==============================] - 5s 7ms/step - loss: 1.4788 - accuracy: 0.6731 - val_loss: 0.6346 - val_accuracy: 0.6589

Epoch 00055: val_loss did not improve from 0.61684

Epoch 56/400

702/702 [==============================] - 5s 7ms/step - loss: 1.4773 - accuracy: 0.6773 - val_loss: 0.6317 - val_accuracy: 0.6613

Epoch 00056: val_loss did not improve from 0.61684

Epoch 57/400

702/702 [==============================] - 5s 7ms/step - loss: 1.4803 - accuracy: 0.6762 - val_loss: 0.6280 - val_accuracy: 0.6695

Epoch 00057: val_loss did not improve from 0.61684

Epoch 58/400

702/702 [==============================] - 5s 7ms/step - loss: 1.4747 - accuracy: 0.6788 - val_loss: 0.6263 - val_accuracy: 0.6695

Epoch 00058: val_loss did not improve from 0.61684

Epoch 59/400

702/702 [==============================] - 5s 7ms/step - loss: 1.4764 - accuracy: 0.6801 - val_loss: 0.6462 - val_accuracy: 0.6494

Epoch 00059: val_loss did not improve from 0.61684

Epoch 60/400

702/702 [==============================] - 5s 7ms/step - loss: 1.4679 - accuracy: 0.6816 - val_loss: 0.6264 - val_accuracy: 0.6689

Epoch 00060: val_loss did not improve from 0.61684

Epoch 61/400

702/702 [==============================] - 5s 7ms/step - loss: 1.4732 - accuracy: 0.6793 - val_loss: 0.6391 - val_accuracy: 0.6546

Epoch 00061: val_loss did not improve from 0.61684

Epoch 62/400

702/702 [==============================] - 5s 7ms/step - loss: 1.4750 - accuracy: 0.6783 - val_loss: 0.6372 - val_accuracy: 0.6559

Epoch 00062: val_loss did not improve from 0.61684

Epoch 63/400

702/702 [==============================] - 5s 7ms/step - loss: 1.4731 - accuracy: 0.6805 - val_loss: 0.6281 - val_accuracy: 0.6700

Epoch 00063: val_loss did not improve from 0.61684

Epoch 64/400

702/702 [==============================] - 5s 7ms/step - loss: 1.4714 - accuracy: 0.6775 - val_loss: 0.6468 - val_accuracy: 0.6451

Epoch 00064: val_loss did not improve from 0.61684

Epoch 65/400

702/702 [==============================] - 5s 7ms/step - loss: 1.4669 - accuracy: 0.6814 - val_loss: 0.6324 - val_accuracy: 0.6627

Epoch 00065: val_loss did not improve from 0.61684

Epoch 66/400

702/702 [==============================] - 5s 7ms/step - loss: 1.4682 - accuracy: 0.6842 - val_loss: 0.6254 - val_accuracy: 0.6687

Epoch 00066: val_loss did not improve from 0.61684

Epoch 67/400

702/702 [==============================] - 5s 7ms/step - loss: 1.4643 - accuracy: 0.6841 - val_loss: 0.6377 - val_accuracy: 0.6546

Epoch 00067: val_loss did not improve from 0.61684

Epoch 68/400

702/702 [==============================] - 5s 7ms/step - loss: 1.4611 - accuracy: 0.6824 - val_loss: 0.6385 - val_accuracy: 0.6565

Epoch 00068: val_loss did not improve from 0.61684

Epoch 69/400

702/702 [==============================] - 5s 7ms/step - loss: 1.4683 - accuracy: 0.6846 - val_loss: 0.6386 - val_accuracy: 0.6611

Epoch 00069: val_loss did not improve from 0.61684

Epoch 70/400

702/702 [==============================] - 5s 7ms/step - loss: 1.4704 - accuracy: 0.6816 - val_loss: 0.6224 - val_accuracy: 0.6711

Epoch 00070: val_loss did not improve from 0.61684

Epoch 71/400

702/702 [==============================] - 5s 7ms/step - loss: 1.4622 - accuracy: 0.6848 - val_loss: 0.6335 - val_accuracy: 0.6627

Epoch 00071: val_loss did not improve from 0.61684

Epoch 72/400

702/702 [==============================] - 5s 7ms/step - loss: 1.4612 - accuracy: 0.6849 - val_loss: 0.6312 - val_accuracy: 0.6600

Epoch 00072: val_loss did not improve from 0.61684

Epoch 73/400

702/702 [==============================] - 5s 7ms/step - loss: 1.4572 - accuracy: 0.6847 - val_loss: 0.6325 - val_accuracy: 0.6608

Epoch 00073: val_loss did not improve from 0.61684

Epoch 74/400

702/702 [==============================] - 5s 7ms/step - loss: 1.4591 - accuracy: 0.6860 - val_loss: 0.6196 - val_accuracy: 0.6751

Epoch 00074: val_loss did not improve from 0.61684

Epoch 75/400

702/702 [==============================] - 5s 7ms/step - loss: 1.4529 - accuracy: 0.6869 - val_loss: 0.6134 - val_accuracy: 0.6833

Epoch 00075: val_loss improved from 0.61684 to 0.61343, saving model to path_to_save_best_model.h5

Epoch 76/400

702/702 [==============================] - 5s 7ms/step - loss: 1.4569 - accuracy: 0.6860 - val_loss: 0.6272 - val_accuracy: 0.6651

Epoch 00076: val_loss did not improve from 0.61343

Epoch 77/400

702/702 [==============================] - 5s 7ms/step - loss: 1.4565 - accuracy: 0.6851 - val_loss: 0.6069 - val_accuracy: 0.6922

Epoch 00077: val_loss improved from 0.61343 to 0.60687, saving model to path_to_save_best_model.h5

Epoch 78/400

702/702 [==============================] - 5s 7ms/step - loss: 1.4495 - accuracy: 0.6918 - val_loss: 0.6287 - val_accuracy: 0.6714

Epoch 00078: val_loss did not improve from 0.60687

Epoch 79/400

702/702 [==============================] - 5s 7ms/step - loss: 1.4572 - accuracy: 0.6886 - val_loss: 0.6334 - val_accuracy: 0.6665

Epoch 00079: val_loss did not improve from 0.60687

Epoch 80/400

702/702 [==============================] - 5s 7ms/step - loss: 1.4557 - accuracy: 0.6864 - val_loss: 0.6454 - val_accuracy: 0.6505

Epoch 00080: val_loss did not improve from 0.60687

Epoch 81/400

702/702 [==============================] - 5s 7ms/step - loss: 1.4496 - accuracy: 0.6912 - val_loss: 0.6214 - val_accuracy: 0.6770

Epoch 00081: val_loss did not improve from 0.60687

Epoch 82/400

702/702 [==============================] - 5s 7ms/step - loss: 1.4558 - accuracy: 0.6886 - val_loss: 0.6211 - val_accuracy: 0.6779

Epoch 00082: val_loss did not improve from 0.60687

Epoch 83/400

702/702 [==============================] - 5s 7ms/step - loss: 1.4497 - accuracy: 0.6915 - val_loss: 0.6300 - val_accuracy: 0.6687

Epoch 00083: val_loss did not improve from 0.60687

Epoch 84/400

702/702 [==============================] - 5s 7ms/step - loss: 1.4576 - accuracy: 0.6861 - val_loss: 0.6198 - val_accuracy: 0.6808

Epoch 00084: val_loss did not improve from 0.60687

Epoch 85/400

702/702 [==============================] - 5s 7ms/step - loss: 1.4484 - accuracy: 0.6914 - val_loss: 0.6275 - val_accuracy: 0.6705

Epoch 00085: val_loss did not improve from 0.60687

Epoch 86/400

702/702 [==============================] - 5s 7ms/step - loss: 1.4489 - accuracy: 0.6934 - val_loss: 0.6149 - val_accuracy: 0.6835

Epoch 00086: val_loss did not improve from 0.60687

Epoch 87/400

702/702 [==============================] - 5s 7ms/step - loss: 1.4504 - accuracy: 0.6913 - val_loss: 0.6193 - val_accuracy: 0.6806

Epoch 00087: val_loss did not improve from 0.60687

Epoch 88/400

702/702 [==============================] - 5s 7ms/step - loss: 1.4432 - accuracy: 0.6939 - val_loss: 0.6230 - val_accuracy: 0.6781

Epoch 00088: val_loss did not improve from 0.60687

Epoch 89/400

702/702 [==============================] - 5s 7ms/step - loss: 1.4533 - accuracy: 0.6862 - val_loss: 0.6157 - val_accuracy: 0.6846

Epoch 00089: val_loss did not improve from 0.60687

Epoch 90/400

702/702 [==============================] - 5s 7ms/step - loss: 1.4503 - accuracy: 0.6922 - val_loss: 0.6248 - val_accuracy: 0.6757

Epoch 00090: val_loss did not improve from 0.60687

Epoch 91/400

702/702 [==============================] - 5s 7ms/step - loss: 1.4428 - accuracy: 0.6928 - val_loss: 0.6095 - val_accuracy: 0.6881

Epoch 00091: val_loss did not improve from 0.60687

Epoch 92/400

702/702 [==============================] - 5s 8ms/step - loss: 1.4440 - accuracy: 0.6926 - val_loss: 0.6144 - val_accuracy: 0.6833

Epoch 00092: val_loss did not improve from 0.60687

Epoch 93/400

702/702 [==============================] - 5s 7ms/step - loss: 1.4441 - accuracy: 0.6921 - val_loss: 0.6307 - val_accuracy: 0.6662

Epoch 00093: val_loss did not improve from 0.60687

Epoch 94/400

702/702 [==============================] - 5s 8ms/step - loss: 1.4502 - accuracy: 0.6893 - val_loss: 0.6326 - val_accuracy: 0.6700

Epoch 00094: val_loss did not improve from 0.60687

Epoch 95/400

702/702 [==============================] - 5s 7ms/step - loss: 1.4446 - accuracy: 0.6908 - val_loss: 0.6124 - val_accuracy: 0.6830

Epoch 00095: val_loss did not improve from 0.60687

Epoch 96/400

702/702 [==============================] - 5s 7ms/step - loss: 1.4397 - accuracy: 0.6937 - val_loss: 0.6238 - val_accuracy: 0.6751

Epoch 00096: val_loss did not improve from 0.60687

Epoch 97/400

702/702 [==============================] - 5s 7ms/step - loss: 1.4435 - accuracy: 0.6909 - val_loss: 0.6043 - val_accuracy: 0.6930

Epoch 00097: val_loss improved from 0.60687 to 0.60428, saving model to path_to_save_best_model.h5

Epoch 98/400

702/702 [==============================] - 5s 7ms/step - loss: 1.4382 - accuracy: 0.6968 - val_loss: 0.6430 - val_accuracy: 0.6570

Epoch 00098: val_loss did not improve from 0.60428

Epoch 99/400

702/702 [==============================] - 5s 7ms/step - loss: 1.4437 - accuracy: 0.6920 - val_loss: 0.6077 - val_accuracy: 0.6911

Epoch 00099: val_loss did not improve from 0.60428

Epoch 100/400

702/702 [==============================] - 5s 8ms/step - loss: 1.4396 - accuracy: 0.6921 - val_loss: 0.6175 - val_accuracy: 0.6814

Epoch 00100: val_loss did not improve from 0.60428

Epoch 101/400

702/702 [==============================] - 5s 7ms/step - loss: 1.4392 - accuracy: 0.6915 - val_loss: 0.6259 - val_accuracy: 0.6749

Epoch 00101: val_loss did not improve from 0.60428

Epoch 102/400

702/702 [==============================] - 5s 8ms/step - loss: 1.4390 - accuracy: 0.6929 - val_loss: 0.6106 - val_accuracy: 0.6857

Epoch 00102: val_loss did not improve from 0.60428

Epoch 103/400

702/702 [==============================] - 5s 7ms/step - loss: 1.4378 - accuracy: 0.6952 - val_loss: 0.6159 - val_accuracy: 0.6846

Epoch 00103: val_loss did not improve from 0.60428

Epoch 104/400

702/702 [==============================] - 5s 7ms/step - loss: 1.4368 - accuracy: 0.6959 - val_loss: 0.6127 - val_accuracy: 0.6868

Epoch 00104: val_loss did not improve from 0.60428

Epoch 105/400

702/702 [==============================] - 5s 7ms/step - loss: 1.4329 - accuracy: 0.6971 - val_loss: 0.6141 - val_accuracy: 0.6846

Epoch 00105: val_loss did not improve from 0.60428

Epoch 106/400

702/702 [==============================] - 5s 7ms/step - loss: 1.4300 - accuracy: 0.6996 - val_loss: 0.6219 - val_accuracy: 0.6768

Epoch 00106: val_loss did not improve from 0.60428

Epoch 107/400

702/702 [==============================] - 5s 7ms/step - loss: 1.4387 - accuracy: 0.6909 - val_loss: 0.6278 - val_accuracy: 0.6776

Epoch 00107: val_loss did not improve from 0.60428

Epoch 108/400

702/702 [==============================] - 5s 7ms/step - loss: 1.4368 - accuracy: 0.6952 - val_loss: 0.6109 - val_accuracy: 0.6906

Epoch 00108: val_loss did not improve from 0.60428

Epoch 109/400

702/702 [==============================] - 5s 7ms/step - loss: 1.4358 - accuracy: 0.6945 - val_loss: 0.6082 - val_accuracy: 0.6895

Epoch 00109: val_loss did not improve from 0.60428

Epoch 110/400

702/702 [==============================] - 5s 7ms/step - loss: 1.4311 - accuracy: 0.6968 - val_loss: 0.6009 - val_accuracy: 0.6957

Epoch 00110: val_loss improved from 0.60428 to 0.60090, saving model to path_to_save_best_model.h5

Epoch 111/400

702/702 [==============================] - 5s 7ms/step - loss: 1.4279 - accuracy: 0.6993 - val_loss: 0.6201 - val_accuracy: 0.6792

Epoch 00111: val_loss did not improve from 0.60090

Epoch 112/400

702/702 [==============================] - 5s 7ms/step - loss: 1.4319 - accuracy: 0.6973 - val_loss: 0.6108 - val_accuracy: 0.6909

Epoch 00112: val_loss did not improve from 0.60090

Epoch 113/400

702/702 [==============================] - 5s 7ms/step - loss: 1.4253 - accuracy: 0.7000 - val_loss: 0.6106 - val_accuracy: 0.6854

Epoch 00113: val_loss did not improve from 0.60090

Epoch 114/400

702/702 [==============================] - 5s 7ms/step - loss: 1.4290 - accuracy: 0.6982 - val_loss: 0.6087 - val_accuracy: 0.6930

Epoch 00114: val_loss did not improve from 0.60090

Epoch 115/400

702/702 [==============================] - 5s 7ms/step - loss: 1.4357 - accuracy: 0.6946 - val_loss: 0.6219 - val_accuracy: 0.6784

Epoch 00115: val_loss did not improve from 0.60090

Epoch 116/400

702/702 [==============================] - 5s 7ms/step - loss: 1.4202 - accuracy: 0.7028 - val_loss: 0.6159 - val_accuracy: 0.6781

Epoch 00116: val_loss did not improve from 0.60090

Epoch 117/400

702/702 [==============================] - 6s 8ms/step - loss: 1.4277 - accuracy: 0.6991 - val_loss: 0.6192 - val_accuracy: 0.6792

Epoch 00117: val_loss did not improve from 0.60090

Epoch 118/400

702/702 [==============================] - 6s 8ms/step - loss: 1.4279 - accuracy: 0.7000 - val_loss: 0.6122 - val_accuracy: 0.6849

Epoch 00118: val_loss did not improve from 0.60090

Epoch 119/400

702/702 [==============================] - 6s 8ms/step - loss: 1.4232 - accuracy: 0.6978 - val_loss: 0.6166 - val_accuracy: 0.6749

Epoch 00119: val_loss did not improve from 0.60090

Epoch 120/400

702/702 [==============================] - 6s 8ms/step - loss: 1.4307 - accuracy: 0.6955 - val_loss: 0.6091 - val_accuracy: 0.6854

Epoch 00120: val_loss did not improve from 0.60090

Epoch 121/400

702/702 [==============================] - 6s 8ms/step - loss: 1.4207 - accuracy: 0.7026 - val_loss: 0.6183 - val_accuracy: 0.6738

Epoch 00121: val_loss did not improve from 0.60090

Epoch 122/400

702/702 [==============================] - 6s 8ms/step - loss: 1.4237 - accuracy: 0.7012 - val_loss: 0.6303 - val_accuracy: 0.6654

Epoch 00122: val_loss did not improve from 0.60090

Epoch 123/400

702/702 [==============================] - 5s 8ms/step - loss: 1.4194 - accuracy: 0.7013 - val_loss: 0.6152 - val_accuracy: 0.6779

Epoch 00123: val_loss did not improve from 0.60090

Epoch 124/400

702/702 [==============================] - 5s 7ms/step - loss: 1.4256 - accuracy: 0.6969 - val_loss: 0.6220 - val_accuracy: 0.6735

Epoch 00124: val_loss did not improve from 0.60090

Epoch 125/400

702/702 [==============================] - 5s 7ms/step - loss: 1.4306 - accuracy: 0.6982 - val_loss: 0.6010 - val_accuracy: 0.6990

Epoch 00125: val_loss did not improve from 0.60090

Epoch 126/400

702/702 [==============================] - 5s 7ms/step - loss: 1.4182 - accuracy: 0.7021 - val_loss: 0.6195 - val_accuracy: 0.6746

Epoch 00126: val_loss did not improve from 0.60090

Epoch 127/400

702/702 [==============================] - 5s 7ms/step - loss: 1.4178 - accuracy: 0.7013 - val_loss: 0.6115 - val_accuracy: 0.6822

Epoch 00127: val_loss did not improve from 0.60090

Epoch 128/400

702/702 [==============================] - 5s 7ms/step - loss: 1.4202 - accuracy: 0.7012 - val_loss: 0.6259 - val_accuracy: 0.6673

Epoch 00128: val_loss did not improve from 0.60090

Epoch 129/400

702/702 [==============================] - 5s 7ms/step - loss: 1.4281 - accuracy: 0.6958 - val_loss: 0.6165 - val_accuracy: 0.6762

Epoch 00129: val_loss did not improve from 0.60090

Epoch 130/400

702/702 [==============================] - 5s 7ms/step - loss: 1.4185 - accuracy: 0.7022 - val_loss: 0.6230 - val_accuracy: 0.6770

Epoch 00130: val_loss did not improve from 0.60090

Epoch 131/400

702/702 [==============================] - 5s 7ms/step - loss: 1.4156 - accuracy: 0.7023 - val_loss: 0.6146 - val_accuracy: 0.6825

Epoch 00131: val_loss did not improve from 0.60090

Epoch 132/400

702/702 [==============================] - 5s 7ms/step - loss: 1.4220 - accuracy: 0.6990 - val_loss: 0.6149 - val_accuracy: 0.6819

Epoch 00132: val_loss did not improve from 0.60090

Epoch 133/400

702/702 [==============================] - 5s 7ms/step - loss: 1.4160 - accuracy: 0.7030 - val_loss: 0.6076 - val_accuracy: 0.6854

Epoch 00133: val_loss did not improve from 0.60090

Epoch 134/400

702/702 [==============================] - 5s 7ms/step - loss: 1.4164 - accuracy: 0.7011 - val_loss: 0.6302 - val_accuracy: 0.6630

Epoch 00134: val_loss did not improve from 0.60090

Epoch 135/400

702/702 [==============================] - 5s 7ms/step - loss: 1.4110 - accuracy: 0.7016 - val_loss: 0.6246 - val_accuracy: 0.6646

Epoch 00135: val_loss did not improve from 0.60090

Epoch 136/400

702/702 [==============================] - 5s 7ms/step - loss: 1.4168 - accuracy: 0.7007 - val_loss: 0.6127 - val_accuracy: 0.6816

Epoch 00136: val_loss did not improve from 0.60090

Epoch 137/400

702/702 [==============================] - 5s 7ms/step - loss: 1.4220 - accuracy: 0.6997 - val_loss: 0.6112 - val_accuracy: 0.6792

Epoch 00137: val_loss did not improve from 0.60090

Epoch 138/400

702/702 [==============================] - 5s 7ms/step - loss: 1.4138 - accuracy: 0.7036 - val_loss: 0.6093 - val_accuracy: 0.6846

Epoch 00138: val_loss did not improve from 0.60090

Epoch 139/400

702/702 [==============================] - 5s 7ms/step - loss: 1.4148 - accuracy: 0.7025 - val_loss: 0.6136 - val_accuracy: 0.6765

Epoch 00139: val_loss did not improve from 0.60090

Epoch 140/400

702/702 [==============================] - 5s 7ms/step - loss: 1.4122 - accuracy: 0.7034 - val_loss: 0.6083 - val_accuracy: 0.6808

Epoch 00140: val_loss did not improve from 0.60090

Epoch 141/400

702/702 [==============================] - 5s 7ms/step - loss: 1.4170 - accuracy: 0.7012 - val_loss: 0.6065 - val_accuracy: 0.6816

Epoch 00141: val_loss did not improve from 0.60090

Epoch 142/400

702/702 [==============================] - 5s 7ms/step - loss: 1.4208 - accuracy: 0.7028 - val_loss: 0.6294 - val_accuracy: 0.6619

Epoch 00142: val_loss did not improve from 0.60090

Epoch 143/400

702/702 [==============================] - 5s 7ms/step - loss: 1.4077 - accuracy: 0.7041 - val_loss: 0.6046 - val_accuracy: 0.6887

Epoch 00143: val_loss did not improve from 0.60090

Epoch 144/400

702/702 [==============================] - 5s 7ms/step - loss: 1.4053 - accuracy: 0.7036 - val_loss: 0.6082 - val_accuracy: 0.6849

Epoch 00144: val_loss did not improve from 0.60090

Epoch 145/400

702/702 [==============================] - 5s 7ms/step - loss: 1.4146 - accuracy: 0.7015 - val_loss: 0.6075 - val_accuracy: 0.6849

Epoch 00145: val_loss did not improve from 0.60090

Epoch 146/400

702/702 [==============================] - 5s 7ms/step - loss: 1.4145 - accuracy: 0.7017 - val_loss: 0.6249 - val_accuracy: 0.6638

Epoch 00146: val_loss did not improve from 0.60090

Epoch 147/400

702/702 [==============================] - 5s 7ms/step - loss: 1.4069 - accuracy: 0.7042 - val_loss: 0.6142 - val_accuracy: 0.6762

Epoch 00147: val_loss did not improve from 0.60090

Epoch 148/400

702/702 [==============================] - 5s 7ms/step - loss: 1.4122 - accuracy: 0.7018 - val_loss: 0.6141 - val_accuracy: 0.6697

Epoch 00148: val_loss did not improve from 0.60090

Epoch 149/400

702/702 [==============================] - 5s 7ms/step - loss: 1.3991 - accuracy: 0.7072 - val_loss: 0.6167 - val_accuracy: 0.6700

Epoch 00149: val_loss did not improve from 0.60090

Epoch 150/400

702/702 [==============================] - 5s 7ms/step - loss: 1.4117 - accuracy: 0.7018 - val_loss: 0.6134 - val_accuracy: 0.6770

Epoch 00150: val_loss did not improve from 0.60090

Epoch 151/400

702/702 [==============================] - 5s 7ms/step - loss: 1.4122 - accuracy: 0.7000 - val_loss: 0.6109 - val_accuracy: 0.6773

Epoch 00151: val_loss did not improve from 0.60090

Epoch 152/400

702/702 [==============================] - 5s 7ms/step - loss: 1.4053 - accuracy: 0.7044 - val_loss: 0.6155 - val_accuracy: 0.6719

Epoch 00152: val_loss did not improve from 0.60090

Epoch 153/400

702/702 [==============================] - 5s 7ms/step - loss: 1.4094 - accuracy: 0.7052 - val_loss: 0.6041 - val_accuracy: 0.6862

Epoch 00153: val_loss did not improve from 0.60090

Epoch 154/400

702/702 [==============================] - 5s 7ms/step - loss: 1.4137 - accuracy: 0.7033 - val_loss: 0.6020 - val_accuracy: 0.6903

Epoch 00154: val_loss did not improve from 0.60090

Epoch 155/400

702/702 [==============================] - 5s 7ms/step - loss: 1.4166 - accuracy: 0.7011 - val_loss: 0.6241 - val_accuracy: 0.6689

Epoch 00155: val_loss did not improve from 0.60090

Epoch 156/400

702/702 [==============================] - 5s 7ms/step - loss: 1.4084 - accuracy: 0.7040 - val_loss: 0.6030 - val_accuracy: 0.6895

Epoch 00156: val_loss did not improve from 0.60090

Epoch 157/400

702/702 [==============================] - 5s 7ms/step - loss: 1.4038 - accuracy: 0.7052 - val_loss: 0.6178 - val_accuracy: 0.6735

Epoch 00157: val_loss did not improve from 0.60090

Epoch 158/400

702/702 [==============================] - 5s 7ms/step - loss: 1.4096 - accuracy: 0.7024 - val_loss: 0.6301 - val_accuracy: 0.6603

Epoch 00158: val_loss did not improve from 0.60090

Epoch 159/400

702/702 [==============================] - 5s 7ms/step - loss: 1.4077 - accuracy: 0.7011 - val_loss: 0.6137 - val_accuracy: 0.6798

Epoch 00159: val_loss did not improve from 0.60090

Epoch 160/400

702/702 [==============================] - 5s 7ms/step - loss: 1.4114 - accuracy: 0.7028 - val_loss: 0.6209 - val_accuracy: 0.6640

Epoch 00160: val_loss did not improve from 0.60090

Epoch 161/400

702/702 [==============================] - 5s 7ms/step - loss: 1.4051 - accuracy: 0.7034 - val_loss: 0.6021 - val_accuracy: 0.6857

Epoch 00161: val_loss did not improve from 0.60090

Epoch 162/400

702/702 [==============================] - 5s 7ms/step - loss: 1.4041 - accuracy: 0.7049 - val_loss: 0.6061 - val_accuracy: 0.6800

Epoch 00162: val_loss did not improve from 0.60090

Epoch 163/400

702/702 [==============================] - 5s 7ms/step - loss: 1.4044 - accuracy: 0.7061 - val_loss: 0.6171 - val_accuracy: 0.6711

Epoch 00163: val_loss did not improve from 0.60090

Epoch 164/400

702/702 [==============================] - 5s 7ms/step - loss: 1.4030 - accuracy: 0.7052 - val_loss: 0.6169 - val_accuracy: 0.6700

Epoch 00164: val_loss did not improve from 0.60090

Epoch 165/400

702/702 [==============================] - 5s 7ms/step - loss: 1.4102 - accuracy: 0.7028 - val_loss: 0.6088 - val_accuracy: 0.6795

Epoch 00165: val_loss did not improve from 0.60090

Epoch 166/400

702/702 [==============================] - 5s 7ms/step - loss: 1.4063 - accuracy: 0.7046 - val_loss: 0.6101 - val_accuracy: 0.6800

Epoch 00166: val_loss did not improve from 0.60090

Epoch 167/400

702/702 [==============================] - 5s 7ms/step - loss: 1.4025 - accuracy: 0.7043 - val_loss: 0.6136 - val_accuracy: 0.6724

Epoch 00167: val_loss did not improve from 0.60090

Epoch 168/400

702/702 [==============================] - 5s 8ms/step - loss: 1.4085 - accuracy: 0.7055 - val_loss: 0.6208 - val_accuracy: 0.6692

Epoch 00168: val_loss did not improve from 0.60090

Epoch 169/400

702/702 [==============================] - 5s 7ms/step - loss: 1.4017 - accuracy: 0.7073 - val_loss: 0.6107 - val_accuracy: 0.6765

Epoch 00169: val_loss did not improve from 0.60090

Epoch 170/400

702/702 [==============================] - 5s 8ms/step - loss: 1.4117 - accuracy: 0.7017 - val_loss: 0.6092 - val_accuracy: 0.6781

Epoch 00170: val_loss did not improve from 0.60090

Epoch 171/400

702/702 [==============================] - 5s 7ms/step - loss: 1.4056 - accuracy: 0.7054 - val_loss: 0.6246 - val_accuracy: 0.6654

Epoch 00171: val_loss did not improve from 0.60090

Epoch 172/400

702/702 [==============================] - 5s 7ms/step - loss: 1.3997 - accuracy: 0.7049 - val_loss: 0.6215 - val_accuracy: 0.6700

Epoch 00172: val_loss did not improve from 0.60090

Epoch 173/400

702/702 [==============================] - 5s 7ms/step - loss: 1.3972 - accuracy: 0.7068 - val_loss: 0.6241 - val_accuracy: 0.6665

Epoch 00173: val_loss did not improve from 0.60090

Epoch 174/400

702/702 [==============================] - 5s 7ms/step - loss: 1.3946 - accuracy: 0.7059 - val_loss: 0.5972 - val_accuracy: 0.6909

Epoch 00174: val_loss improved from 0.60090 to 0.59722, saving model to path_to_save_best_model.h5

Epoch 175/400

702/702 [==============================] - 5s 7ms/step - loss: 1.3969 - accuracy: 0.7083 - val_loss: 0.6337 - val_accuracy: 0.6622

Epoch 00175: val_loss did not improve from 0.59722

Epoch 176/400

702/702 [==============================] - 5s 7ms/step - loss: 1.4014 - accuracy: 0.7056 - val_loss: 0.6090 - val_accuracy: 0.6798

Epoch 00176: val_loss did not improve from 0.59722

Epoch 177/400

702/702 [==============================] - 5s 7ms/step - loss: 1.3948 - accuracy: 0.7090 - val_loss: 0.6115 - val_accuracy: 0.6776

Epoch 00177: val_loss did not improve from 0.59722

Epoch 178/400

702/702 [==============================] - 5s 7ms/step - loss: 1.3969 - accuracy: 0.7084 - val_loss: 0.6027 - val_accuracy: 0.6857

Epoch 00178: val_loss did not improve from 0.59722

Epoch 179/400

702/702 [==============================] - 5s 7ms/step - loss: 1.4006 - accuracy: 0.7091 - val_loss: 0.6171 - val_accuracy: 0.6697

Epoch 00179: val_loss did not improve from 0.59722

Epoch 180/400

702/702 [==============================] - 5s 7ms/step - loss: 1.3996 - accuracy: 0.7074 - val_loss: 0.6169 - val_accuracy: 0.6714

Epoch 00180: val_loss did not improve from 0.59722

Epoch 181/400

702/702 [==============================] - 5s 7ms/step - loss: 1.3983 - accuracy: 0.7087 - val_loss: 0.6052 - val_accuracy: 0.6816

Epoch 00181: val_loss did not improve from 0.59722

Epoch 182/400

702/702 [==============================] - 5s 7ms/step - loss: 1.4029 - accuracy: 0.7036 - val_loss: 0.6088 - val_accuracy: 0.6811

Epoch 00182: val_loss did not improve from 0.59722

Epoch 183/400

702/702 [==============================] - 5s 7ms/step - loss: 1.3977 - accuracy: 0.7086 - val_loss: 0.6071 - val_accuracy: 0.6800

Epoch 00183: val_loss did not improve from 0.59722

Epoch 184/400

702/702 [==============================] - 5s 7ms/step - loss: 1.3978 - accuracy: 0.7065 - val_loss: 0.6120 - val_accuracy: 0.6781

Epoch 00184: val_loss did not improve from 0.59722

Epoch 185/400

702/702 [==============================] - 5s 8ms/step - loss: 1.3908 - accuracy: 0.7081 - val_loss: 0.6100 - val_accuracy: 0.6776

Epoch 00185: val_loss did not improve from 0.59722

Epoch 186/400

702/702 [==============================] - 5s 7ms/step - loss: 1.3911 - accuracy: 0.7120 - val_loss: 0.6068 - val_accuracy: 0.6814

Epoch 00186: val_loss did not improve from 0.59722

Epoch 187/400

702/702 [==============================] - 5s 7ms/step - loss: 1.4018 - accuracy: 0.7070 - val_loss: 0.6091 - val_accuracy: 0.6806

Epoch 00187: val_loss did not improve from 0.59722

Epoch 188/400

702/702 [==============================] - 5s 7ms/step - loss: 1.3952 - accuracy: 0.7099 - val_loss: 0.6033 - val_accuracy: 0.6844

Epoch 00188: val_loss did not improve from 0.59722

Epoch 189/400

702/702 [==============================] - 5s 7ms/step - loss: 1.4044 - accuracy: 0.7017 - val_loss: 0.6074 - val_accuracy: 0.6754

Epoch 00189: val_loss did not improve from 0.59722

Epoch 190/400

702/702 [==============================] - 5s 7ms/step - loss: 1.3992 - accuracy: 0.7043 - val_loss: 0.5997 - val_accuracy: 0.6890

Epoch 00190: val_loss did not improve from 0.59722

Epoch 191/400

702/702 [==============================] - 5s 7ms/step - loss: 1.3956 - accuracy: 0.7091 - val_loss: 0.5967 - val_accuracy: 0.6922

Epoch 00191: val_loss improved from 0.59722 to 0.59669, saving model to path_to_save_best_model.h5

Epoch 192/400

702/702 [==============================] - 5s 7ms/step - loss: 1.3876 - accuracy: 0.7116 - val_loss: 0.6129 - val_accuracy: 0.6743

Epoch 00192: val_loss did not improve from 0.59669

Epoch 193/400

702/702 [==============================] - 5s 7ms/step - loss: 1.3938 - accuracy: 0.7077 - val_loss: 0.6057 - val_accuracy: 0.6852

Epoch 00193: val_loss did not improve from 0.59669

Epoch 194/400

702/702 [==============================] - 5s 7ms/step - loss: 1.3963 - accuracy: 0.7078 - val_loss: 0.6010 - val_accuracy: 0.6825

Epoch 00194: val_loss did not improve from 0.59669

Epoch 195/400

702/702 [==============================] - 5s 7ms/step - loss: 1.3941 - accuracy: 0.7117 - val_loss: 0.6265 - val_accuracy: 0.6651

Epoch 00195: val_loss did not improve from 0.59669

Epoch 196/400

702/702 [==============================] - 5s 7ms/step - loss: 1.3959 - accuracy: 0.7082 - val_loss: 0.6085 - val_accuracy: 0.6846

Epoch 00196: val_loss did not improve from 0.59669

Epoch 197/400

702/702 [==============================] - 5s 7ms/step - loss: 1.3922 - accuracy: 0.7094 - val_loss: 0.5995 - val_accuracy: 0.6865

Epoch 00197: val_loss did not improve from 0.59669

Epoch 198/400

702/702 [==============================] - 5s 7ms/step - loss: 1.3992 - accuracy: 0.7078 - val_loss: 0.6233 - val_accuracy: 0.6659

Epoch 00198: val_loss did not improve from 0.59669

Epoch 199/400

702/702 [==============================] - 5s 7ms/step - loss: 1.3911 - accuracy: 0.7084 - val_loss: 0.6112 - val_accuracy: 0.6754

Epoch 00199: val_loss did not improve from 0.59669

Epoch 200/400

702/702 [==============================] - 5s 7ms/step - loss: 1.3900 - accuracy: 0.7100 - val_loss: 0.6107 - val_accuracy: 0.6760

Epoch 00200: val_loss did not improve from 0.59669

Epoch 201/400

702/702 [==============================] - 5s 7ms/step - loss: 1.3914 - accuracy: 0.7078 - val_loss: 0.6037 - val_accuracy: 0.6808

Epoch 00201: val_loss did not improve from 0.59669

Epoch 202/400

702/702 [==============================] - 5s 7ms/step - loss: 1.3875 - accuracy: 0.7118 - val_loss: 0.6100 - val_accuracy: 0.6733

Epoch 00202: val_loss did not improve from 0.59669

Epoch 203/400

702/702 [==============================] - 5s 7ms/step - loss: 1.3894 - accuracy: 0.7093 - val_loss: 0.6036 - val_accuracy: 0.6881

Epoch 00203: val_loss did not improve from 0.59669

Epoch 204/400

702/702 [==============================] - 5s 8ms/step - loss: 1.3886 - accuracy: 0.7098 - val_loss: 0.5993 - val_accuracy: 0.6871

Epoch 00204: val_loss did not improve from 0.59669

Epoch 205/400

702/702 [==============================] - 5s 8ms/step - loss: 1.3872 - accuracy: 0.7100 - val_loss: 0.6080 - val_accuracy: 0.6773

Epoch 00205: val_loss did not improve from 0.59669

Epoch 206/400

702/702 [==============================] - 5s 8ms/step - loss: 1.3871 - accuracy: 0.7110 - val_loss: 0.6066 - val_accuracy: 0.6789

Epoch 00206: val_loss did not improve from 0.59669

Epoch 207/400

702/702 [==============================] - 5s 7ms/step - loss: 1.3845 - accuracy: 0.7161 - val_loss: 0.6079 - val_accuracy: 0.6787

Epoch 00207: val_loss did not improve from 0.59669

Epoch 208/400

702/702 [==============================] - 5s 7ms/step - loss: 1.3875 - accuracy: 0.7106 - val_loss: 0.6150 - val_accuracy: 0.6719

Epoch 00208: val_loss did not improve from 0.59669

Epoch 209/400

702/702 [==============================] - 5s 7ms/step - loss: 1.3903 - accuracy: 0.7089 - val_loss: 0.5949 - val_accuracy: 0.6898

Epoch 00209: val_loss improved from 0.59669 to 0.59488, saving model to path_to_save_best_model.h5

Epoch 210/400

702/702 [==============================] - 5s 7ms/step - loss: 1.3864 - accuracy: 0.7100 - val_loss: 0.6077 - val_accuracy: 0.6803

Epoch 00210: val_loss did not improve from 0.59488

Epoch 211/400

702/702 [==============================] - 5s 7ms/step - loss: 1.3901 - accuracy: 0.7104 - val_loss: 0.6009 - val_accuracy: 0.6846

Epoch 00211: val_loss did not improve from 0.59488

Epoch 212/400

702/702 [==============================] - 5s 7ms/step - loss: 1.3928 - accuracy: 0.7112 - val_loss: 0.6090 - val_accuracy: 0.6754

Epoch 00212: val_loss did not improve from 0.59488

Epoch 213/400

702/702 [==============================] - 5s 7ms/step - loss: 1.3860 - accuracy: 0.7111 - val_loss: 0.5983 - val_accuracy: 0.6849

Epoch 00213: val_loss did not improve from 0.59488

Epoch 214/400

702/702 [==============================] - 5s 7ms/step - loss: 1.3938 - accuracy: 0.7115 - val_loss: 0.5998 - val_accuracy: 0.6860

Epoch 00214: val_loss did not improve from 0.59488

Epoch 215/400

702/702 [==============================] - 5s 7ms/step - loss: 1.3814 - accuracy: 0.7130 - val_loss: 0.6017 - val_accuracy: 0.6827

Epoch 00215: val_loss did not improve from 0.59488

Epoch 216/400

702/702 [==============================] - 5s 7ms/step - loss: 1.3809 - accuracy: 0.7131 - val_loss: 0.5970 - val_accuracy: 0.6944

Epoch 00216: val_loss did not improve from 0.59488

Epoch 217/400

702/702 [==============================] - 5s 7ms/step - loss: 1.3792 - accuracy: 0.7153 - val_loss: 0.5937 - val_accuracy: 0.6911

Epoch 00217: val_loss improved from 0.59488 to 0.59369, saving model to path_to_save_best_model.h5

Epoch 218/400

702/702 [==============================] - 5s 7ms/step - loss: 1.3771 - accuracy: 0.7128 - val_loss: 0.6046 - val_accuracy: 0.6862

Epoch 00218: val_loss did not improve from 0.59369

Epoch 219/400

702/702 [==============================] - 5s 7ms/step - loss: 1.3775 - accuracy: 0.7149 - val_loss: 0.5964 - val_accuracy: 0.6930

Epoch 00219: val_loss did not improve from 0.59369

Epoch 220/400

702/702 [==============================] - 5s 7ms/step - loss: 1.3796 - accuracy: 0.7119 - val_loss: 0.6026 - val_accuracy: 0.6884

Epoch 00220: val_loss did not improve from 0.59369

Epoch 221/400

702/702 [==============================] - 5s 7ms/step - loss: 1.3783 - accuracy: 0.7147 - val_loss: 0.6053 - val_accuracy: 0.6784

Epoch 00221: val_loss did not improve from 0.59369

Epoch 222/400

702/702 [==============================] - 5s 7ms/step - loss: 1.3865 - accuracy: 0.7093 - val_loss: 0.5966 - val_accuracy: 0.6960

Epoch 00222: val_loss did not improve from 0.59369

Epoch 223/400

702/702 [==============================] - 5s 7ms/step - loss: 1.3851 - accuracy: 0.7135 - val_loss: 0.6058 - val_accuracy: 0.6803

Epoch 00223: val_loss did not improve from 0.59369

Epoch 224/400

702/702 [==============================] - 5s 7ms/step - loss: 1.3789 - accuracy: 0.7126 - val_loss: 0.6026 - val_accuracy: 0.6808

Epoch 00224: val_loss did not improve from 0.59369

Epoch 225/400

702/702 [==============================] - 5s 7ms/step - loss: 1.3833 - accuracy: 0.7106 - val_loss: 0.5941 - val_accuracy: 0.6927

Epoch 00225: val_loss did not improve from 0.59369

Epoch 226/400

702/702 [==============================] - 5s 7ms/step - loss: 1.3748 - accuracy: 0.7144 - val_loss: 0.5976 - val_accuracy: 0.6930

Epoch 00226: val_loss did not improve from 0.59369

Epoch 227/400

702/702 [==============================] - 5s 7ms/step - loss: 1.3776 - accuracy: 0.7127 - val_loss: 0.5920 - val_accuracy: 0.6960

Epoch 00227: val_loss improved from 0.59369 to 0.59202, saving model to path_to_save_best_model.h5

Epoch 228/400

702/702 [==============================] - 5s 7ms/step - loss: 1.3794 - accuracy: 0.7131 - val_loss: 0.5941 - val_accuracy: 0.6995

Epoch 00228: val_loss did not improve from 0.59202

Epoch 229/400

702/702 [==============================] - 5s 7ms/step - loss: 1.3795 - accuracy: 0.7150 - val_loss: 0.6035 - val_accuracy: 0.6800

Epoch 00229: val_loss did not improve from 0.59202

Epoch 230/400

702/702 [==============================] - 5s 7ms/step - loss: 1.3750 - accuracy: 0.7149 - val_loss: 0.6004 - val_accuracy: 0.6860

Epoch 00230: val_loss did not improve from 0.59202

Epoch 231/400

702/702 [==============================] - 5s 7ms/step - loss: 1.3759 - accuracy: 0.7150 - val_loss: 0.6047 - val_accuracy: 0.6830

Epoch 00231: val_loss did not improve from 0.59202

Epoch 232/400

702/702 [==============================] - 5s 7ms/step - loss: 1.3776 - accuracy: 0.7137 - val_loss: 0.5970 - val_accuracy: 0.6927

Epoch 00232: val_loss did not improve from 0.59202

Epoch 233/400

702/702 [==============================] - 5s 7ms/step - loss: 1.3869 - accuracy: 0.7104 - val_loss: 0.5991 - val_accuracy: 0.6868

Epoch 00233: val_loss did not improve from 0.59202

Epoch 234/400

702/702 [==============================] - 5s 7ms/step - loss: 1.3795 - accuracy: 0.7116 - val_loss: 0.6053 - val_accuracy: 0.6800

Epoch 00234: val_loss did not improve from 0.59202

Epoch 235/400

702/702 [==============================] - 5s 7ms/step - loss: 1.3753 - accuracy: 0.7140 - val_loss: 0.6035 - val_accuracy: 0.6827

Epoch 00235: val_loss did not improve from 0.59202

Epoch 236/400

702/702 [==============================] - 5s 7ms/step - loss: 1.3795 - accuracy: 0.7132 - val_loss: 0.5896 - val_accuracy: 0.7011

Epoch 00236: val_loss improved from 0.59202 to 0.58961, saving model to path_to_save_best_model.h5

Epoch 237/400

702/702 [==============================] - 5s 7ms/step - loss: 1.3789 - accuracy: 0.7136 - val_loss: 0.5971 - val_accuracy: 0.6887

Epoch 00237: val_loss did not improve from 0.58961

Epoch 238/400

702/702 [==============================] - 5s 7ms/step - loss: 1.3652 - accuracy: 0.7171 - val_loss: 0.5881 - val_accuracy: 0.6987

Epoch 00238: val_loss improved from 0.58961 to 0.58812, saving model to path_to_save_best_model.h5

Epoch 239/400

702/702 [==============================] - 5s 7ms/step - loss: 1.3770 - accuracy: 0.7148 - val_loss: 0.5918 - val_accuracy: 0.6946

Epoch 00239: val_loss did not improve from 0.58812

Epoch 240/400

702/702 [==============================] - 5s 7ms/step - loss: 1.3798 - accuracy: 0.7143 - val_loss: 0.5940 - val_accuracy: 0.6952

Epoch 00240: val_loss did not improve from 0.58812

Epoch 241/400

702/702 [==============================] - 5s 7ms/step - loss: 1.3701 - accuracy: 0.7179 - val_loss: 0.6006 - val_accuracy: 0.6865

Epoch 00241: val_loss did not improve from 0.58812

Epoch 242/400

702/702 [==============================] - 5s 7ms/step - loss: 1.3780 - accuracy: 0.7149 - val_loss: 0.5941 - val_accuracy: 0.6936

Epoch 00242: val_loss did not improve from 0.58812

Epoch 243/400

702/702 [==============================] - 5s 7ms/step - loss: 1.3823 - accuracy: 0.7122 - val_loss: 0.5940 - val_accuracy: 0.6963

Epoch 00243: val_loss did not improve from 0.58812

Epoch 244/400

702/702 [==============================] - 5s 7ms/step - loss: 1.3797 - accuracy: 0.7132 - val_loss: 0.6102 - val_accuracy: 0.6792

Epoch 00244: val_loss did not improve from 0.58812

Epoch 245/400

702/702 [==============================] - 5s 7ms/step - loss: 1.3757 - accuracy: 0.7139 - val_loss: 0.5967 - val_accuracy: 0.6849

Epoch 00245: val_loss did not improve from 0.58812

Epoch 246/400

702/702 [==============================] - 5s 7ms/step - loss: 1.3667 - accuracy: 0.7175 - val_loss: 0.6010 - val_accuracy: 0.6814

Epoch 00246: val_loss did not improve from 0.58812

Epoch 247/400

702/702 [==============================] - 5s 7ms/step - loss: 1.3666 - accuracy: 0.7173 - val_loss: 0.6101 - val_accuracy: 0.6789

Epoch 00247: val_loss did not improve from 0.58812

Epoch 248/400

702/702 [==============================] - 5s 7ms/step - loss: 1.3767 - accuracy: 0.7099 - val_loss: 0.6179 - val_accuracy: 0.6735

Epoch 00248: val_loss did not improve from 0.58812

Epoch 249/400

702/702 [==============================] - 5s 7ms/step - loss: 1.3699 - accuracy: 0.7184 - val_loss: 0.6045 - val_accuracy: 0.6806

Epoch 00249: val_loss did not improve from 0.58812

Epoch 250/400

702/702 [==============================] - 5s 7ms/step - loss: 1.3728 - accuracy: 0.7171 - val_loss: 0.6093 - val_accuracy: 0.6784

Epoch 00250: val_loss did not improve from 0.58812

Epoch 251/400

702/702 [==============================] - 5s 7ms/step - loss: 1.3675 - accuracy: 0.7159 - val_loss: 0.6032 - val_accuracy: 0.6830

Epoch 00251: val_loss did not improve from 0.58812

Epoch 252/400

702/702 [==============================] - 6s 8ms/step - loss: 1.3730 - accuracy: 0.7175 - val_loss: 0.5911 - val_accuracy: 0.6949

Epoch 00252: val_loss did not improve from 0.58812

Epoch 253/400

702/702 [==============================] - 5s 7ms/step - loss: 1.3641 - accuracy: 0.7219 - val_loss: 0.5872 - val_accuracy: 0.6973

Epoch 00253: val_loss improved from 0.58812 to 0.58720, saving model to path_to_save_best_model.h5

Epoch 254/400

702/702 [==============================] - 5s 7ms/step - loss: 1.3664 - accuracy: 0.7198 - val_loss: 0.5895 - val_accuracy: 0.6946

Epoch 00254: val_loss did not improve from 0.58720

Epoch 255/400

702/702 [==============================] - 5s 7ms/step - loss: 1.3683 - accuracy: 0.7183 - val_loss: 0.6025 - val_accuracy: 0.6857

Epoch 00255: val_loss did not improve from 0.58720

Epoch 256/400

702/702 [==============================] - 5s 7ms/step - loss: 1.3657 - accuracy: 0.7184 - val_loss: 0.5988 - val_accuracy: 0.6895

Epoch 00256: val_loss did not improve from 0.58720

Epoch 257/400

702/702 [==============================] - 5s 8ms/step - loss: 1.3663 - accuracy: 0.7181 - val_loss: 0.5966 - val_accuracy: 0.6909

Epoch 00257: val_loss did not improve from 0.58720

Epoch 258/400

702/702 [==============================] - 5s 7ms/step - loss: 1.3667 - accuracy: 0.7178 - val_loss: 0.6026 - val_accuracy: 0.6849

Epoch 00258: val_loss did not improve from 0.58720

Epoch 259/400

702/702 [==============================] - 5s 7ms/step - loss: 1.3676 - accuracy: 0.7147 - val_loss: 0.5952 - val_accuracy: 0.6881

Epoch 00259: val_loss did not improve from 0.58720

Epoch 260/400

702/702 [==============================] - 5s 8ms/step - loss: 1.3624 - accuracy: 0.7210 - val_loss: 0.6077 - val_accuracy: 0.6760

Epoch 00260: val_loss did not improve from 0.58720

Epoch 261/400

702/702 [==============================] - 5s 8ms/step - loss: 1.3651 - accuracy: 0.7170 - val_loss: 0.5914 - val_accuracy: 0.6941

Epoch 00261: val_loss did not improve from 0.58720

Epoch 262/400

702/702 [==============================] - 5s 8ms/step - loss: 1.3643 - accuracy: 0.7183 - val_loss: 0.5908 - val_accuracy: 0.6922

Epoch 00262: val_loss did not improve from 0.58720

Epoch 263/400

702/702 [==============================] - 5s 7ms/step - loss: 1.3616 - accuracy: 0.7210 - val_loss: 0.5997 - val_accuracy: 0.6852

Epoch 00263: val_loss did not improve from 0.58720

Epoch 264/400

702/702 [==============================] - 5s 7ms/step - loss: 1.3562 - accuracy: 0.7206 - val_loss: 0.5963 - val_accuracy: 0.6925

Epoch 00264: val_loss did not improve from 0.58720

Epoch 265/400

702/702 [==============================] - 5s 7ms/step - loss: 1.3619 - accuracy: 0.7183 - val_loss: 0.5889 - val_accuracy: 0.7009

Epoch 00265: val_loss did not improve from 0.58720

Epoch 266/400

702/702 [==============================] - 5s 7ms/step - loss: 1.3658 - accuracy: 0.7164 - val_loss: 0.5894 - val_accuracy: 0.6995

Epoch 00266: val_loss did not improve from 0.58720

Epoch 267/400

702/702 [==============================] - 5s 7ms/step - loss: 1.3656 - accuracy: 0.7180 - val_loss: 0.6118 - val_accuracy: 0.6757

Epoch 00267: val_loss did not improve from 0.58720

Epoch 268/400

702/702 [==============================] - 5s 7ms/step - loss: 1.3634 - accuracy: 0.7175 - val_loss: 0.5985 - val_accuracy: 0.6884

Epoch 00268: val_loss did not improve from 0.58720

Epoch 269/400

702/702 [==============================] - 5s 7ms/step - loss: 1.3570 - accuracy: 0.7226 - val_loss: 0.5927 - val_accuracy: 0.6930

Epoch 00269: val_loss did not improve from 0.58720

Epoch 270/400

702/702 [==============================] - 5s 7ms/step - loss: 1.3580 - accuracy: 0.7212 - val_loss: 0.5870 - val_accuracy: 0.6960

Epoch 00270: val_loss improved from 0.58720 to 0.58699, saving model to path_to_save_best_model.h5

Epoch 271/400

702/702 [==============================] - 5s 7ms/step - loss: 1.3608 - accuracy: 0.7203 - val_loss: 0.5943 - val_accuracy: 0.6938

Epoch 00271: val_loss did not improve from 0.58699

Epoch 272/400

702/702 [==============================] - 5s 7ms/step - loss: 1.3683 - accuracy: 0.7175 - val_loss: 0.5918 - val_accuracy: 0.6984

Epoch 00272: val_loss did not improve from 0.58699

Epoch 273/400

702/702 [==============================] - 5s 8ms/step - loss: 1.3629 - accuracy: 0.7193 - val_loss: 0.5926 - val_accuracy: 0.6944

Epoch 00273: val_loss did not improve from 0.58699

Epoch 274/400

702/702 [==============================] - 5s 8ms/step - loss: 1.3711 - accuracy: 0.7152 - val_loss: 0.5996 - val_accuracy: 0.6857

Epoch 00274: val_loss did not improve from 0.58699

Epoch 275/400

702/702 [==============================] - 5s 7ms/step - loss: 1.3557 - accuracy: 0.7197 - val_loss: 0.5867 - val_accuracy: 0.6952

Epoch 00275: val_loss improved from 0.58699 to 0.58667, saving model to path_to_save_best_model.h5

Epoch 276/400

702/702 [==============================] - 5s 7ms/step - loss: 1.3598 - accuracy: 0.7207 - val_loss: 0.5945 - val_accuracy: 0.6936

Epoch 00276: val_loss did not improve from 0.58667

Epoch 277/400

702/702 [==============================] - 5s 7ms/step - loss: 1.3594 - accuracy: 0.7187 - val_loss: 0.5921 - val_accuracy: 0.6949

Epoch 00277: val_loss did not improve from 0.58667

Epoch 278/400

702/702 [==============================] - 5s 7ms/step - loss: 1.3641 - accuracy: 0.7182 - val_loss: 0.5959 - val_accuracy: 0.6949

Epoch 00278: val_loss did not improve from 0.58667

Epoch 279/400

702/702 [==============================] - 5s 7ms/step - loss: 1.3547 - accuracy: 0.7228 - val_loss: 0.5904 - val_accuracy: 0.7003

Epoch 00279: val_loss did not improve from 0.58667

Epoch 280/400

702/702 [==============================] - 5s 7ms/step - loss: 1.3593 - accuracy: 0.7193 - val_loss: 0.5888 - val_accuracy: 0.6979

Epoch 00280: val_loss did not improve from 0.58667

Epoch 281/400

702/702 [==============================] - 5s 7ms/step - loss: 1.3593 - accuracy: 0.7214 - val_loss: 0.5928 - val_accuracy: 0.6914

Epoch 00281: val_loss did not improve from 0.58667

Epoch 282/400

702/702 [==============================] - 5s 7ms/step - loss: 1.3562 - accuracy: 0.7222 - val_loss: 0.6062 - val_accuracy: 0.6852

Epoch 00282: val_loss did not improve from 0.58667

Epoch 283/400

702/702 [==============================] - 5s 7ms/step - loss: 1.3555 - accuracy: 0.7214 - val_loss: 0.5850 - val_accuracy: 0.7025

Epoch 00283: val_loss improved from 0.58667 to 0.58504, saving model to path_to_save_best_model.h5

Epoch 284/400

702/702 [==============================] - 5s 7ms/step - loss: 1.3586 - accuracy: 0.7202 - val_loss: 0.5925 - val_accuracy: 0.6949

Epoch 00284: val_loss did not improve from 0.58504

Epoch 285/400

702/702 [==============================] - 5s 7ms/step - loss: 1.3611 - accuracy: 0.7186 - val_loss: 0.5980 - val_accuracy: 0.6936

Epoch 00285: val_loss did not improve from 0.58504

Epoch 286/400

702/702 [==============================] - 5s 7ms/step - loss: 1.3642 - accuracy: 0.7190 - val_loss: 0.5883 - val_accuracy: 0.6965

Epoch 00286: val_loss did not improve from 0.58504

Epoch 287/400

702/702 [==============================] - 5s 7ms/step - loss: 1.3550 - accuracy: 0.7230 - val_loss: 0.6118 - val_accuracy: 0.6757

Epoch 00287: val_loss did not improve from 0.58504

Epoch 288/400

702/702 [==============================] - 5s 7ms/step - loss: 1.3467 - accuracy: 0.7239 - val_loss: 0.6130 - val_accuracy: 0.6779

Epoch 00288: val_loss did not improve from 0.58504

Epoch 289/400

702/702 [==============================] - 5s 7ms/step - loss: 1.3570 - accuracy: 0.7194 - val_loss: 0.6011 - val_accuracy: 0.6857

Epoch 00289: val_loss did not improve from 0.58504

Epoch 290/400

702/702 [==============================] - 5s 8ms/step - loss: 1.3563 - accuracy: 0.7205 - val_loss: 0.5947 - val_accuracy: 0.6968

Epoch 00290: val_loss did not improve from 0.58504

Epoch 291/400

702/702 [==============================] - 5s 8ms/step - loss: 1.3562 - accuracy: 0.7189 - val_loss: 0.5933 - val_accuracy: 0.6938

Epoch 00291: val_loss did not improve from 0.58504

Epoch 292/400

702/702 [==============================] - 5s 7ms/step - loss: 1.3475 - accuracy: 0.7237 - val_loss: 0.5915 - val_accuracy: 0.6952

Epoch 00292: val_loss did not improve from 0.58504

Epoch 293/400

702/702 [==============================] - 5s 7ms/step - loss: 1.3585 - accuracy: 0.7194 - val_loss: 0.5825 - val_accuracy: 0.7047

Epoch 00293: val_loss improved from 0.58504 to 0.58250, saving model to path_to_save_best_model.h5

Epoch 294/400

702/702 [==============================] - 5s 7ms/step - loss: 1.3469 - accuracy: 0.7241 - val_loss: 0.6028 - val_accuracy: 0.6800

Epoch 00294: val_loss did not improve from 0.58250

Epoch 295/400

702/702 [==============================] - 5s 7ms/step - loss: 1.3587 - accuracy: 0.7188 - val_loss: 0.5812 - val_accuracy: 0.7033

Epoch 00295: val_loss improved from 0.58250 to 0.58120, saving model to path_to_save_best_model.h5

Epoch 296/400

702/702 [==============================] - 5s 7ms/step - loss: 1.3499 - accuracy: 0.7241 - val_loss: 0.5902 - val_accuracy: 0.6957

Epoch 00296: val_loss did not improve from 0.58120

Epoch 297/400

702/702 [==============================] - 5s 7ms/step - loss: 1.3494 - accuracy: 0.7232 - val_loss: 0.6023 - val_accuracy: 0.6873

Epoch 00297: val_loss did not improve from 0.58120

Epoch 298/400

702/702 [==============================] - 5s 7ms/step - loss: 1.3502 - accuracy: 0.7212 - val_loss: 0.5938 - val_accuracy: 0.6941

Epoch 00298: val_loss did not improve from 0.58120

Epoch 299/400

702/702 [==============================] - 5s 7ms/step - loss: 1.3518 - accuracy: 0.7214 - val_loss: 0.5858 - val_accuracy: 0.6984

Epoch 00299: val_loss did not improve from 0.58120

Epoch 300/400

702/702 [==============================] - 5s 7ms/step - loss: 1.3464 - accuracy: 0.7234 - val_loss: 0.5978 - val_accuracy: 0.6917

Epoch 00300: val_loss did not improve from 0.58120

Epoch 301/400

702/702 [==============================] - 5s 7ms/step - loss: 1.3574 - accuracy: 0.7224 - val_loss: 0.5887 - val_accuracy: 0.7014

Epoch 00301: val_loss did not improve from 0.58120

Epoch 302/400

702/702 [==============================] - 5s 7ms/step - loss: 1.3464 - accuracy: 0.7235 - val_loss: 0.5894 - val_accuracy: 0.6965

Epoch 00302: val_loss did not improve from 0.58120

Epoch 303/400

702/702 [==============================] - 5s 7ms/step - loss: 1.3458 - accuracy: 0.7258 - val_loss: 0.5842 - val_accuracy: 0.7001

Epoch 00303: val_loss did not improve from 0.58120

Epoch 304/400

702/702 [==============================] - 5s 7ms/step - loss: 1.3505 - accuracy: 0.7201 - val_loss: 0.5926 - val_accuracy: 0.6925

Epoch 00304: val_loss did not improve from 0.58120

Epoch 305/400

702/702 [==============================] - 5s 7ms/step - loss: 1.3515 - accuracy: 0.7230 - val_loss: 0.5861 - val_accuracy: 0.6968

Epoch 00305: val_loss did not improve from 0.58120

Epoch 306/400

702/702 [==============================] - 5s 7ms/step - loss: 1.3529 - accuracy: 0.7214 - val_loss: 0.5905 - val_accuracy: 0.6922

Epoch 00306: val_loss did not improve from 0.58120

Epoch 307/400

702/702 [==============================] - 5s 7ms/step - loss: 1.3528 - accuracy: 0.7218 - val_loss: 0.5866 - val_accuracy: 0.6973

Epoch 00307: val_loss did not improve from 0.58120

Epoch 308/400

702/702 [==============================] - 5s 7ms/step - loss: 1.3418 - accuracy: 0.7262 - val_loss: 0.5840 - val_accuracy: 0.7033

Epoch 00308: val_loss did not improve from 0.58120

Epoch 309/400

702/702 [==============================] - 5s 7ms/step - loss: 1.3493 - accuracy: 0.7229 - val_loss: 0.5902 - val_accuracy: 0.6944

Epoch 00309: val_loss did not improve from 0.58120

Epoch 310/400

702/702 [==============================] - 5s 7ms/step - loss: 1.3525 - accuracy: 0.7200 - val_loss: 0.5936 - val_accuracy: 0.6957

Epoch 00310: val_loss did not improve from 0.58120

Epoch 311/400

702/702 [==============================] - 5s 7ms/step - loss: 1.3505 - accuracy: 0.7215 - val_loss: 0.5870 - val_accuracy: 0.6973

Epoch 00311: val_loss did not improve from 0.58120

Epoch 312/400

702/702 [==============================] - 5s 7ms/step - loss: 1.3538 - accuracy: 0.7220 - val_loss: 0.5827 - val_accuracy: 0.7055

Epoch 00312: val_loss did not improve from 0.58120

Epoch 313/400

702/702 [==============================] - 5s 7ms/step - loss: 1.3479 - accuracy: 0.7238 - val_loss: 0.5901 - val_accuracy: 0.6973

Epoch 00313: val_loss did not improve from 0.58120

Epoch 314/400

702/702 [==============================] - 5s 7ms/step - loss: 1.3410 - accuracy: 0.7250 - val_loss: 0.5966 - val_accuracy: 0.6919

Epoch 00314: val_loss did not improve from 0.58120

Epoch 315/400

702/702 [==============================] - 5s 7ms/step - loss: 1.3509 - accuracy: 0.7221 - val_loss: 0.5904 - val_accuracy: 0.6938

Epoch 00315: val_loss did not improve from 0.58120

Epoch 316/400

702/702 [==============================] - 5s 7ms/step - loss: 1.3501 - accuracy: 0.7225 - val_loss: 0.5979 - val_accuracy: 0.6871

Epoch 00316: val_loss did not improve from 0.58120

Epoch 317/400

702/702 [==============================] - 5s 7ms/step - loss: 1.3534 - accuracy: 0.7193 - val_loss: 0.5893 - val_accuracy: 0.6973

Epoch 00317: val_loss did not improve from 0.58120

Epoch 318/400

702/702 [==============================] - 5s 7ms/step - loss: 1.3458 - accuracy: 0.7248 - val_loss: 0.5875 - val_accuracy: 0.6992

Epoch 00318: val_loss did not improve from 0.58120

Epoch 319/400

702/702 [==============================] - 5s 7ms/step - loss: 1.3496 - accuracy: 0.7230 - val_loss: 0.6080 - val_accuracy: 0.6784

Epoch 00319: val_loss did not improve from 0.58120

Epoch 320/400

702/702 [==============================] - 5s 7ms/step - loss: 1.3414 - accuracy: 0.7263 - val_loss: 0.5982 - val_accuracy: 0.6876

Epoch 00320: val_loss did not improve from 0.58120

Epoch 321/400

702/702 [==============================] - 5s 7ms/step - loss: 1.3512 - accuracy: 0.7230 - val_loss: 0.5946 - val_accuracy: 0.6890

Epoch 00321: val_loss did not improve from 0.58120

Epoch 322/400

702/702 [==============================] - 5s 8ms/step - loss: 1.3397 - accuracy: 0.7257 - val_loss: 0.5873 - val_accuracy: 0.6952

Epoch 00322: val_loss did not improve from 0.58120

Epoch 323/400

702/702 [==============================] - 5s 7ms/step - loss: 1.3439 - accuracy: 0.7235 - val_loss: 0.5947 - val_accuracy: 0.6922

Epoch 00323: val_loss did not improve from 0.58120

Epoch 324/400

702/702 [==============================] - 5s 7ms/step - loss: 1.3470 - accuracy: 0.7235 - val_loss: 0.5802 - val_accuracy: 0.7022

Epoch 00324: val_loss improved from 0.58120 to 0.58024, saving model to path_to_save_best_model.h5

Epoch 325/400

702/702 [==============================] - 5s 7ms/step - loss: 1.3398 - accuracy: 0.7269 - val_loss: 0.5919 - val_accuracy: 0.6914

Epoch 00325: val_loss did not improve from 0.58024

Epoch 326/400

702/702 [==============================] - 5s 8ms/step - loss: 1.3480 - accuracy: 0.7245 - val_loss: 0.5998 - val_accuracy: 0.6833

Epoch 00326: val_loss did not improve from 0.58024

Epoch 327/400

702/702 [==============================] - 5s 7ms/step - loss: 1.3392 - accuracy: 0.7251 - val_loss: 0.5818 - val_accuracy: 0.6984

Epoch 00327: val_loss did not improve from 0.58024

Epoch 328/400

702/702 [==============================] - 5s 7ms/step - loss: 1.3492 - accuracy: 0.7263 - val_loss: 0.5830 - val_accuracy: 0.7011

Epoch 00328: val_loss did not improve from 0.58024

Epoch 329/400

702/702 [==============================] - 5s 7ms/step - loss: 1.3422 - accuracy: 0.7269 - val_loss: 0.5934 - val_accuracy: 0.6941

Epoch 00329: val_loss did not improve from 0.58024

Epoch 330/400

702/702 [==============================] - 5s 7ms/step - loss: 1.3450 - accuracy: 0.7241 - val_loss: 0.6002 - val_accuracy: 0.6895

Epoch 00330: val_loss did not improve from 0.58024

Epoch 331/400

702/702 [==============================] - 5s 7ms/step - loss: 1.3445 - accuracy: 0.7257 - val_loss: 0.5844 - val_accuracy: 0.7003

Epoch 00331: val_loss did not improve from 0.58024

Epoch 332/400

702/702 [==============================] - 5s 7ms/step - loss: 1.3399 - accuracy: 0.7251 - val_loss: 0.5848 - val_accuracy: 0.7014

Epoch 00332: val_loss did not improve from 0.58024

Epoch 333/400

702/702 [==============================] - 5s 7ms/step - loss: 1.3468 - accuracy: 0.7233 - val_loss: 0.6004 - val_accuracy: 0.6873

Epoch 00333: val_loss did not improve from 0.58024

Epoch 334/400

702/702 [==============================] - 5s 7ms/step - loss: 1.3461 - accuracy: 0.7210 - val_loss: 0.5903 - val_accuracy: 0.6984

Epoch 00334: val_loss did not improve from 0.58024

Epoch 335/400

702/702 [==============================] - 5s 7ms/step - loss: 1.3419 - accuracy: 0.7253 - val_loss: 0.5797 - val_accuracy: 0.7060

Epoch 00335: val_loss improved from 0.58024 to 0.57970, saving model to path_to_save_best_model.h5

Epoch 336/400

702/702 [==============================] - 5s 7ms/step - loss: 1.3402 - accuracy: 0.7274 - val_loss: 0.5777 - val_accuracy: 0.7068

Epoch 00336: val_loss improved from 0.57970 to 0.57766, saving model to path_to_save_best_model.h5

Epoch 337/400

702/702 [==============================] - 5s 7ms/step - loss: 1.3434 - accuracy: 0.7253 - val_loss: 0.5834 - val_accuracy: 0.7019

Epoch 00337: val_loss did not improve from 0.57766

Epoch 338/400

702/702 [==============================] - 5s 7ms/step - loss: 1.3423 - accuracy: 0.7252 - val_loss: 0.5878 - val_accuracy: 0.6971

Epoch 00338: val_loss did not improve from 0.57766

Epoch 339/400

702/702 [==============================] - 5s 7ms/step - loss: 1.3468 - accuracy: 0.7229 - val_loss: 0.5900 - val_accuracy: 0.6968

Epoch 00339: val_loss did not improve from 0.57766

Epoch 340/400

702/702 [==============================] - 5s 7ms/step - loss: 1.3412 - accuracy: 0.7240 - val_loss: 0.5733 - val_accuracy: 0.7109

Epoch 00340: val_loss improved from 0.57766 to 0.57325, saving model to path_to_save_best_model.h5

Epoch 341/400

702/702 [==============================] - 5s 7ms/step - loss: 1.3418 - accuracy: 0.7266 - val_loss: 0.5837 - val_accuracy: 0.7011

Epoch 00341: val_loss did not improve from 0.57325

Epoch 342/400

702/702 [==============================] - 5s 7ms/step - loss: 1.3433 - accuracy: 0.7258 - val_loss: 0.5773 - val_accuracy: 0.7049

Epoch 00342: val_loss did not improve from 0.57325

Epoch 343/400

702/702 [==============================] - 5s 8ms/step - loss: 1.3406 - accuracy: 0.7249 - val_loss: 0.5982 - val_accuracy: 0.6871

Epoch 00343: val_loss did not improve from 0.57325

Epoch 344/400

702/702 [==============================] - 5s 7ms/step - loss: 1.3381 - accuracy: 0.7255 - val_loss: 0.5723 - val_accuracy: 0.7122

Epoch 00344: val_loss improved from 0.57325 to 0.57228, saving model to path_to_save_best_model.h5

Epoch 345/400

702/702 [==============================] - 5s 7ms/step - loss: 1.3406 - accuracy: 0.7262 - val_loss: 0.5856 - val_accuracy: 0.6987

Epoch 00345: val_loss did not improve from 0.57228

Epoch 346/400

702/702 [==============================] - 5s 7ms/step - loss: 1.3413 - accuracy: 0.7249 - val_loss: 0.5847 - val_accuracy: 0.6998

Epoch 00346: val_loss did not improve from 0.57228

Epoch 347/400

702/702 [==============================] - 5s 7ms/step - loss: 1.3357 - accuracy: 0.7279 - val_loss: 0.5870 - val_accuracy: 0.7014

Epoch 00347: val_loss did not improve from 0.57228

Epoch 348/400

702/702 [==============================] - 5s 7ms/step - loss: 1.3406 - accuracy: 0.7256 - val_loss: 0.5853 - val_accuracy: 0.6976

Epoch 00348: val_loss did not improve from 0.57228

Epoch 349/400

702/702 [==============================] - 5s 7ms/step - loss: 1.3329 - accuracy: 0.7257 - val_loss: 0.5813 - val_accuracy: 0.7025

Epoch 00349: val_loss did not improve from 0.57228

Epoch 350/400

702/702 [==============================] - 5s 7ms/step - loss: 1.3406 - accuracy: 0.7258 - val_loss: 0.5904 - val_accuracy: 0.6927

Epoch 00350: val_loss did not improve from 0.57228

Epoch 351/400

702/702 [==============================] - 5s 7ms/step - loss: 1.3460 - accuracy: 0.7207 - val_loss: 0.5890 - val_accuracy: 0.6957

Epoch 00351: val_loss did not improve from 0.57228

Epoch 352/400

702/702 [==============================] - 5s 7ms/step - loss: 1.3373 - accuracy: 0.7258 - val_loss: 0.5934 - val_accuracy: 0.6930

Epoch 00352: val_loss did not improve from 0.57228

Epoch 353/400

702/702 [==============================] - 5s 7ms/step - loss: 1.3402 - accuracy: 0.7239 - val_loss: 0.5918 - val_accuracy: 0.6944

Epoch 00353: val_loss did not improve from 0.57228

Epoch 354/400

702/702 [==============================] - 5s 7ms/step - loss: 1.3390 - accuracy: 0.7253 - val_loss: 0.5914 - val_accuracy: 0.6938

Epoch 00354: val_loss did not improve from 0.57228

Epoch 355/400

702/702 [==============================] - 5s 7ms/step - loss: 1.3441 - accuracy: 0.7220 - val_loss: 0.5826 - val_accuracy: 0.7019

Epoch 00355: val_loss did not improve from 0.57228

Epoch 356/400

702/702 [==============================] - 5s 7ms/step - loss: 1.3449 - accuracy: 0.7217 - val_loss: 0.5900 - val_accuracy: 0.6903

Epoch 00356: val_loss did not improve from 0.57228

Epoch 357/400

702/702 [==============================] - 5s 8ms/step - loss: 1.3443 - accuracy: 0.7235 - val_loss: 0.5980 - val_accuracy: 0.6881

Epoch 00357: val_loss did not improve from 0.57228

Epoch 358/400

702/702 [==============================] - 5s 7ms/step - loss: 1.3422 - accuracy: 0.7249 - val_loss: 0.5912 - val_accuracy: 0.6965

Epoch 00358: val_loss did not improve from 0.57228

Epoch 359/400

702/702 [==============================] - 5s 7ms/step - loss: 1.3313 - accuracy: 0.7266 - val_loss: 0.5916 - val_accuracy: 0.6949

Epoch 00359: val_loss did not improve from 0.57228

Epoch 360/400

702/702 [==============================] - 5s 7ms/step - loss: 1.3328 - accuracy: 0.7292 - val_loss: 0.5899 - val_accuracy: 0.6963

Epoch 00360: val_loss did not improve from 0.57228

Epoch 361/400

702/702 [==============================] - 5s 7ms/step - loss: 1.3325 - accuracy: 0.7255 - val_loss: 0.5902 - val_accuracy: 0.6914

Epoch 00361: val_loss did not improve from 0.57228

Epoch 362/400

702/702 [==============================] - 5s 7ms/step - loss: 1.3374 - accuracy: 0.7241 - val_loss: 0.5877 - val_accuracy: 0.6968

Epoch 00362: val_loss did not improve from 0.57228

Epoch 363/400

702/702 [==============================] - 5s 7ms/step - loss: 1.3371 - accuracy: 0.7259 - val_loss: 0.5871 - val_accuracy: 0.6971

Epoch 00363: val_loss did not improve from 0.57228

Epoch 364/400

702/702 [==============================] - 5s 8ms/step - loss: 1.3369 - accuracy: 0.7265 - val_loss: 0.5883 - val_accuracy: 0.6979

Epoch 00364: val_loss did not improve from 0.57228

Epoch 365/400

702/702 [==============================] - 5s 7ms/step - loss: 1.3327 - accuracy: 0.7286 - val_loss: 0.5999 - val_accuracy: 0.6846

Epoch 00365: val_loss did not improve from 0.57228

Epoch 366/400

702/702 [==============================] - 5s 7ms/step - loss: 1.3336 - accuracy: 0.7282 - val_loss: 0.5778 - val_accuracy: 0.7033

Epoch 00366: val_loss did not improve from 0.57228

Epoch 367/400

702/702 [==============================] - 5s 7ms/step - loss: 1.3346 - accuracy: 0.7295 - val_loss: 0.5968 - val_accuracy: 0.6892

Epoch 00367: val_loss did not improve from 0.57228

Epoch 368/400

702/702 [==============================] - 5s 7ms/step - loss: 1.3306 - accuracy: 0.7272 - val_loss: 0.5839 - val_accuracy: 0.7001

Epoch 00368: val_loss did not improve from 0.57228

Epoch 369/400

702/702 [==============================] - 5s 7ms/step - loss: 1.3236 - accuracy: 0.7278 - val_loss: 0.5981 - val_accuracy: 0.6890

Epoch 00369: val_loss did not improve from 0.57228

Epoch 370/400

702/702 [==============================] - 5s 7ms/step - loss: 1.3360 - accuracy: 0.7249 - val_loss: 0.5749 - val_accuracy: 0.7117

Epoch 00370: val_loss did not improve from 0.57228

Epoch 371/400

702/702 [==============================] - 5s 7ms/step - loss: 1.3312 - accuracy: 0.7279 - val_loss: 0.5829 - val_accuracy: 0.6998

Epoch 00371: val_loss did not improve from 0.57228

Epoch 372/400

702/702 [==============================] - 5s 7ms/step - loss: 1.3448 - accuracy: 0.7249 - val_loss: 0.5915 - val_accuracy: 0.6925

Epoch 00372: val_loss did not improve from 0.57228

Epoch 373/400

702/702 [==============================] - 5s 7ms/step - loss: 1.3275 - accuracy: 0.7291 - val_loss: 0.5820 - val_accuracy: 0.7017

Epoch 00373: val_loss did not improve from 0.57228

Epoch 374/400

702/702 [==============================] - 5s 7ms/step - loss: 1.3339 - accuracy: 0.7297 - val_loss: 0.5910 - val_accuracy: 0.6965

Epoch 00374: val_loss did not improve from 0.57228

Epoch 375/400

702/702 [==============================] - 5s 7ms/step - loss: 1.3415 - accuracy: 0.7242 - val_loss: 0.5812 - val_accuracy: 0.6998

Epoch 00375: val_loss did not improve from 0.57228

Epoch 376/400

702/702 [==============================] - 5s 7ms/step - loss: 1.3329 - accuracy: 0.7276 - val_loss: 0.5787 - val_accuracy: 0.7036

Epoch 00376: val_loss did not improve from 0.57228

Epoch 377/400

702/702 [==============================] - 5s 7ms/step - loss: 1.3410 - accuracy: 0.7252 - val_loss: 0.5823 - val_accuracy: 0.6990

Epoch 00377: val_loss did not improve from 0.57228

Epoch 378/400

702/702 [==============================] - 5s 7ms/step - loss: 1.3338 - accuracy: 0.7249 - val_loss: 0.5804 - val_accuracy: 0.7014

Epoch 00378: val_loss did not improve from 0.57228

Epoch 379/400

702/702 [==============================] - 5s 7ms/step - loss: 1.3311 - accuracy: 0.7294 - val_loss: 0.5931 - val_accuracy: 0.6914

Epoch 00379: val_loss did not improve from 0.57228

Epoch 380/400

702/702 [==============================] - 5s 8ms/step - loss: 1.3267 - accuracy: 0.7264 - val_loss: 0.5739 - val_accuracy: 0.7060

Epoch 00380: val_loss did not improve from 0.57228

Epoch 381/400

702/702 [==============================] - 5s 8ms/step - loss: 1.3288 - accuracy: 0.7291 - val_loss: 0.5896 - val_accuracy: 0.6960

Epoch 00381: val_loss did not improve from 0.57228

Epoch 382/400

702/702 [==============================] - 6s 8ms/step - loss: 1.3236 - accuracy: 0.7283 - val_loss: 0.5893 - val_accuracy: 0.6965

Epoch 00382: val_loss did not improve from 0.57228

Epoch 383/400

702/702 [==============================] - 6s 8ms/step - loss: 1.3291 - accuracy: 0.7268 - val_loss: 0.5860 - val_accuracy: 0.6960

Epoch 00383: val_loss did not improve from 0.57228

Epoch 384/400

702/702 [==============================] - 6s 8ms/step - loss: 1.3247 - accuracy: 0.7329 - val_loss: 0.5910 - val_accuracy: 0.6941

Epoch 00384: val_loss did not improve from 0.57228

Epoch 385/400

702/702 [==============================] - 5s 7ms/step - loss: 1.3281 - accuracy: 0.7273 - val_loss: 0.5825 - val_accuracy: 0.6998

Epoch 00385: val_loss did not improve from 0.57228

Epoch 386/400

702/702 [==============================] - 5s 7ms/step - loss: 1.3319 - accuracy: 0.7278 - val_loss: 0.5905 - val_accuracy: 0.6919

Epoch 00386: val_loss did not improve from 0.57228

Epoch 387/400

702/702 [==============================] - 5s 7ms/step - loss: 1.3329 - accuracy: 0.7264 - val_loss: 0.5796 - val_accuracy: 0.7006

Epoch 00387: val_loss did not improve from 0.57228

Epoch 388/400

702/702 [==============================] - 5s 7ms/step - loss: 1.3322 - accuracy: 0.7296 - val_loss: 0.5829 - val_accuracy: 0.6992

Epoch 00388: val_loss did not improve from 0.57228

Epoch 389/400

702/702 [==============================] - 5s 7ms/step - loss: 1.3313 - accuracy: 0.7291 - val_loss: 0.5942 - val_accuracy: 0.6925

Epoch 00389: val_loss did not improve from 0.57228

Epoch 390/400

702/702 [==============================] - 5s 7ms/step - loss: 1.3324 - accuracy: 0.7268 - val_loss: 0.5861 - val_accuracy: 0.6982

Epoch 00390: val_loss did not improve from 0.57228

Epoch 391/400

702/702 [==============================] - 5s 7ms/step - loss: 1.3260 - accuracy: 0.7296 - val_loss: 0.5881 - val_accuracy: 0.6955

Epoch 00391: val_loss did not improve from 0.57228

Epoch 392/400

702/702 [==============================] - 5s 7ms/step - loss: 1.3274 - accuracy: 0.7266 - val_loss: 0.5806 - val_accuracy: 0.6976

Epoch 00392: val_loss did not improve from 0.57228

Epoch 393/400

702/702 [==============================] - 5s 7ms/step - loss: 1.3220 - accuracy: 0.7292 - val_loss: 0.5904 - val_accuracy: 0.6976

Epoch 00393: val_loss did not improve from 0.57228

Epoch 394/400

702/702 [==============================] - 5s 7ms/step - loss: 1.3330 - accuracy: 0.7282 - val_loss: 0.5906 - val_accuracy: 0.6968

Epoch 00394: val_loss did not improve from 0.57228

Epoch 395/400

702/702 [==============================] - 5s 7ms/step - loss: 1.3220 - accuracy: 0.7312 - val_loss: 0.5810 - val_accuracy: 0.6987

Epoch 00395: val_loss did not improve from 0.57228

Epoch 396/400

702/702 [==============================] - 5s 7ms/step - loss: 1.3336 - accuracy: 0.7270 - val_loss: 0.5824 - val_accuracy: 0.6987

Epoch 00396: val_loss did not improve from 0.57228

Epoch 397/400

702/702 [==============================] - 5s 7ms/step - loss: 1.3222 - accuracy: 0.7311 - val_loss: 0.5784 - val_accuracy: 0.7028

Epoch 00397: val_loss did not improve from 0.57228

Epoch 398/400

702/702 [==============================] - 5s 7ms/step - loss: 1.3255 - accuracy: 0.7292 - val_loss: 0.5722 - val_accuracy: 0.7090

Epoch 00398: val_loss improved from 0.57228 to 0.57219, saving model to path_to_save_best_model.h5

Epoch 399/400

702/702 [==============================] - 5s 7ms/step - loss: 1.3212 - accuracy: 0.7326 - val_loss: 0.5809 - val_accuracy: 0.7003

Epoch 00399: val_loss did not improve from 0.57219

Epoch 400/400

702/702 [==============================] - 5s 7ms/step - loss: 1.3194 - accuracy: 0.7315 - val_loss: 0.5696 - val_accuracy: 0.7128

Epoch 00400: val_loss improved from 0.57219 to 0.56960, saving model to path_to_save_best_model.h5

292/292 [==============================] - 1s 2ms/step - loss: 0.6652 - accuracy: 0.6484
