# Supplementary material for: Deep learning using electroencephalogram (EEG) data for diagnosing and predicting SSRI response in major depressive disorder
Source: Commun Med (Lond). 2026 Mar 23;6:159. doi: 10.1038/s43856-026-01394-z (PMC13009148; doi:10.1038/s43856-026-01394-z)
Supplement: Supplementary file 5 — Supplementary Data 2 [file 43856_2026_1394_MOESM5_ESM.docx]

HC versus MDD

!pip install mne

import numpy as np

import mne

import matplotlib.pyplot as plt

# Define channel names and their importance values

channel_names = ['F7', 'F4', 'P3', 'O1', 'F3', 'C4', 'F8', 'O2', 'P4', 'C3']

importance_values = np.array([1.0,0.83409677, 0.56269719, 0.29139646, 0.28436652, 0.49349136, 0.40583912, 0.34559426, 0.39971955, 0.33263613])

# Load the standard_1020 montage

montage = mne.channels.make_standard_montage('standard_1020')

# Create info structure for raw data

info = mne.create_info(ch_names=channel_names, sfreq=1, ch_types='eeg')

info.set_montage(montage)

# Create the Raw data object

data = importance_values.reshape(-1, 1)  # Shape (n_channels, n_times)

raw = mne.io.RawArray(data, info)

# Plot the importance as a topomap

fig, ax = plt.subplots()

topo_plot = mne.viz.plot_topomap(data.flatten(), raw.info, axes=ax, cmap='bwr', show=False, sensors='ko', sphere=0.1)

# Get the position for the F7 electrode from the layout

pos = mne.channels.layout.find_layout(raw.info).pos[raw.ch_names.index('F7')]

# Annotate the F7 channel name

ax.annotate('F7', xy=(pos[0], pos[1]), textcoords='offset points',

            xytext=(0,5), ha='center', va='bottom', color='blue', fontsize=12)

ax.set_title('Channel Importance for Classification')

plt.colorbar(topo_plot[0], ax=ax, format='%.2f')

plt.show()

R versus NR

!pip install mne

import numpy as np

import mne

import matplotlib.pyplot as plt

# Define channel names and their importance values

channel_names = ['F7', 'F4', 'P3', 'O1', 'F3', 'C4', 'F8', 'O2', 'P4', 'C3']

importance_values = np.array([100,77.2875,43.7846,0,0.293885,27.4472,19.6776,12.2711,17.5653,15.21])

# Load the standard_1020 montage

montage = mne.channels.make_standard_montage('standard_1020')

# Create info structure for raw data

info = mne.create_info(ch_names=channel_names, sfreq=1, ch_types='eeg')

info.set_montage(montage)

# Create the Raw data object

data = importance_values.reshape(-1, 1)  # Shape (n_channels, n_times)

raw = mne.io.RawArray(data, info)

# Plot the importance as a topomap

fig, ax = plt.subplots()

topo_plot = mne.viz.plot_topomap(data.flatten(), raw.info, axes=ax, cmap='bwr', show=False, sensors='ko', sphere=0.1)

# Get the position for the F7 electrode from the layout

pos = mne.channels.layout.find_layout(raw.info).pos[raw.ch_names.index('F7')]

# Annotate the F7 channel name

ax.annotate('F7', xy=(pos[0], pos[1]), textcoords='offset points',

            xytext=(0,5), ha='center', va='bottom', color='blue', fontsize=12)

ax.set_title('Channel Importance for Classification')

plt.colorbar(topo_plot[0], ax=ax, format='%.2f')

plt.show()
