# Supplementary material for: Deep learning using electroencephalogram (EEG) data for diagnosing and predicting SSRI response in major depressive disorder
Source: Commun Med (Lond). 2026 Mar 23;6:159. doi: 10.1038/s43856-026-01394-z (PMC13009148; doi:10.1038/s43856-026-01394-z)
Supplement: Supplementary file 6 — Supplementary Data 3 [file 43856_2026_1394_MOESM6_ESM.docx]

R versus NR

Total params: 4,576,402

Trainable params: 4,576,402

Non-trainable params: 0

_________________________________________________________________

Epoch 1/900

355/355 [==============================] - 14s 21ms/step - loss: 12.7494 - accuracy: 0.4927 - val_loss: 0.6932 - val_accuracy: 0.4536

Epoch 2/900

355/355 [==============================] - 6s 18ms/step - loss: 12.7337 - accuracy: 0.4752 - val_loss: 0.6916 - val_accuracy: 0.4949

Epoch 3/900

355/355 [==============================] - 6s 18ms/step - loss: 12.7248 - accuracy: 0.4847 - val_loss: 0.6896 - val_accuracy: 0.5437

Epoch 4/900

355/355 [==============================] - 6s 18ms/step - loss: 12.7043 - accuracy: 0.4864 - val_loss: 0.6867 - val_accuracy: 0.5587

Epoch 5/900

355/355 [==============================] - 6s 18ms/step - loss: 12.6839 - accuracy: 0.5068 - val_loss: 0.6850 - val_accuracy: 0.5512

Epoch 6/900

355/355 [==============================] - 6s 18ms/step - loss: 12.6636 - accuracy: 0.5089 - val_loss: 0.6829 - val_accuracy: 0.5550

Epoch 7/900

355/355 [==============================] - 6s 18ms/step - loss: 12.6429 - accuracy: 0.5230 - val_loss: 0.6829 - val_accuracy: 0.5383

Epoch 8/900

355/355 [==============================] - 6s 18ms/step - loss: 12.6438 - accuracy: 0.5099 - val_loss: 0.6816 - val_accuracy: 0.5475

Epoch 9/900

355/355 [==============================] - 6s 18ms/step - loss: 12.6225 - accuracy: 0.5137 - val_loss: 0.6807 - val_accuracy: 0.5442

Epoch 10/900

355/355 [==============================] - 6s 18ms/step - loss: 12.6206 - accuracy: 0.5233 - val_loss: 0.6820 - val_accuracy: 0.5367

Epoch 11/900

355/355 [==============================] - 6s 18ms/step - loss: 12.6239 - accuracy: 0.5123 - val_loss: 0.6806 - val_accuracy: 0.5416

Epoch 12/900

355/355 [==============================] - 6s 18ms/step - loss: 12.6153 - accuracy: 0.5159 - val_loss: 0.6807 - val_accuracy: 0.5405

Epoch 13/900

355/355 [==============================] - 6s 18ms/step - loss: 12.6307 - accuracy: 0.5192 - val_loss: 0.6807 - val_accuracy: 0.5362

Epoch 14/900

355/355 [==============================] - 6s 18ms/step - loss: 12.6063 - accuracy: 0.5149 - val_loss: 0.6804 - val_accuracy: 0.5346

Epoch 15/900

355/355 [==============================] - 6s 18ms/step - loss: 12.5994 - accuracy: 0.5164 - val_loss: 0.6789 - val_accuracy: 0.5426

Epoch 16/900

355/355 [==============================] - 6s 18ms/step - loss: 12.5847 - accuracy: 0.5177 - val_loss: 0.6794 - val_accuracy: 0.5378

Epoch 17/900

355/355 [==============================] - 6s 18ms/step - loss: 12.5969 - accuracy: 0.5183 - val_loss: 0.6795 - val_accuracy: 0.5282

Epoch 18/900

355/355 [==============================] - 6s 18ms/step - loss: 12.5765 - accuracy: 0.5229 - val_loss: 0.6784 - val_accuracy: 0.5378

Epoch 19/900

355/355 [==============================] - 6s 18ms/step - loss: 12.5700 - accuracy: 0.5176 - val_loss: 0.6798 - val_accuracy: 0.5217

Epoch 20/900

355/355 [==============================] - 6s 18ms/step - loss: 12.5751 - accuracy: 0.5231 - val_loss: 0.6784 - val_accuracy: 0.5319

Epoch 21/900

355/355 [==============================] - 7s 18ms/step - loss: 12.5897 - accuracy: 0.5219 - val_loss: 0.6770 - val_accuracy: 0.5389

Epoch 22/900

355/355 [==============================] - 7s 18ms/step - loss: 12.5636 - accuracy: 0.5256 - val_loss: 0.6771 - val_accuracy: 0.5340

Epoch 23/900

355/355 [==============================] - 7s 18ms/step - loss: 12.5758 - accuracy: 0.5248 - val_loss: 0.6773 - val_accuracy: 0.5319

Epoch 24/900

355/355 [==============================] - 7s 18ms/step - loss: 12.5718 - accuracy: 0.5222 - val_loss: 0.6762 - val_accuracy: 0.5378

Epoch 25/900

355/355 [==============================] - 7s 18ms/step - loss: 12.5580 - accuracy: 0.5306 - val_loss: 0.6776 - val_accuracy: 0.5292

Epoch 26/900

355/355 [==============================] - 7s 18ms/step - loss: 12.5605 - accuracy: 0.5223 - val_loss: 0.6774 - val_accuracy: 0.5287

Epoch 27/900

355/355 [==============================] - 7s 18ms/step - loss: 12.5604 - accuracy: 0.5232 - val_loss: 0.6764 - val_accuracy: 0.5308

Epoch 28/900

355/355 [==============================] - 7s 18ms/step - loss: 12.5317 - accuracy: 0.5324 - val_loss: 0.6754 - val_accuracy: 0.5378

Epoch 29/900

355/355 [==============================] - 7s 18ms/step - loss: 12.5460 - accuracy: 0.5299 - val_loss: 0.6767 - val_accuracy: 0.5335

Epoch 30/900

355/355 [==============================] - 7s 18ms/step - loss: 12.5153 - accuracy: 0.5290 - val_loss: 0.6758 - val_accuracy: 0.5351

Epoch 31/900

355/355 [==============================] - 7s 18ms/step - loss: 12.5534 - accuracy: 0.5343 - val_loss: 0.6749 - val_accuracy: 0.5448

Epoch 32/900

355/355 [==============================] - 7s 18ms/step - loss: 12.4927 - accuracy: 0.5380 - val_loss: 0.6732 - val_accuracy: 0.5517

Epoch 33/900

355/355 [==============================] - 7s 18ms/step - loss: 12.5431 - accuracy: 0.5370 - val_loss: 0.6732 - val_accuracy: 0.5560

Epoch 34/900

355/355 [==============================] - 7s 19ms/step - loss: 12.5242 - accuracy: 0.5367 - val_loss: 0.6716 - val_accuracy: 0.5625

Epoch 35/900

355/355 [==============================] - 7s 19ms/step - loss: 12.5017 - accuracy: 0.5391 - val_loss: 0.6721 - val_accuracy: 0.5592

Epoch 36/900

355/355 [==============================] - 7s 18ms/step - loss: 12.4860 - accuracy: 0.5449 - val_loss: 0.6744 - val_accuracy: 0.5480

Epoch 37/900

355/355 [==============================] - 7s 18ms/step - loss: 12.4959 - accuracy: 0.5336 - val_loss: 0.6724 - val_accuracy: 0.5625

Epoch 38/900

355/355 [==============================] - 7s 18ms/step - loss: 12.4864 - accuracy: 0.5405 - val_loss: 0.6745 - val_accuracy: 0.5475

Epoch 39/900

355/355 [==============================] - 7s 18ms/step - loss: 12.5124 - accuracy: 0.5447 - val_loss: 0.6735 - val_accuracy: 0.5539

Epoch 40/900

355/355 [==============================] - 7s 19ms/step - loss: 12.4998 - accuracy: 0.5417 - val_loss: 0.6716 - val_accuracy: 0.5705

Epoch 41/900

355/355 [==============================] - 7s 19ms/step - loss: 12.4845 - accuracy: 0.5384 - val_loss: 0.6723 - val_accuracy: 0.5646

Epoch 42/900

355/355 [==============================] - 7s 19ms/step - loss: 12.5227 - accuracy: 0.5461 - val_loss: 0.6707 - val_accuracy: 0.5689

Epoch 43/900

355/355 [==============================] - 7s 18ms/step - loss: 12.4871 - accuracy: 0.5425 - val_loss: 0.6737 - val_accuracy: 0.5517

Epoch 44/900

355/355 [==============================] - 7s 19ms/step - loss: 12.4769 - accuracy: 0.5452 - val_loss: 0.6704 - val_accuracy: 0.5721

Epoch 45/900

355/355 [==============================] - 7s 19ms/step - loss: 12.4303 - accuracy: 0.5504 - val_loss: 0.6707 - val_accuracy: 0.5700

Epoch 46/900

355/355 [==============================] - 7s 19ms/step - loss: 12.4997 - accuracy: 0.5455 - val_loss: 0.6696 - val_accuracy: 0.5748

Epoch 47/900

355/355 [==============================] - 7s 18ms/step - loss: 12.4345 - accuracy: 0.5473 - val_loss: 0.6712 - val_accuracy: 0.5700

Epoch 48/900

355/355 [==============================] - 7s 19ms/step - loss: 12.4680 - accuracy: 0.5456 - val_loss: 0.6703 - val_accuracy: 0.5743

Epoch 49/900

355/355 [==============================] - 7s 19ms/step - loss: 12.4539 - accuracy: 0.5447 - val_loss: 0.6699 - val_accuracy: 0.5796

Epoch 50/900

355/355 [==============================] - 7s 19ms/step - loss: 12.4712 - accuracy: 0.5492 - val_loss: 0.6716 - val_accuracy: 0.5625

Epoch 51/900

355/355 [==============================] - 7s 19ms/step - loss: 12.4592 - accuracy: 0.5429 - val_loss: 0.6681 - val_accuracy: 0.5818

Epoch 52/900

355/355 [==============================] - 7s 19ms/step - loss: 12.4179 - accuracy: 0.5499 - val_loss: 0.6684 - val_accuracy: 0.5834

Epoch 53/900

355/355 [==============================] - 7s 19ms/step - loss: 12.4087 - accuracy: 0.5491 - val_loss: 0.6691 - val_accuracy: 0.5759

Epoch 54/900

355/355 [==============================] - 7s 19ms/step - loss: 12.3856 - accuracy: 0.5519 - val_loss: 0.6670 - val_accuracy: 0.5818

Epoch 55/900

355/355 [==============================] - 7s 19ms/step - loss: 12.3983 - accuracy: 0.5613 - val_loss: 0.6672 - val_accuracy: 0.5818

Epoch 56/900

355/355 [==============================] - 7s 19ms/step - loss: 12.4392 - accuracy: 0.5597 - val_loss: 0.6658 - val_accuracy: 0.5877

Epoch 57/900

355/355 [==============================] - 7s 19ms/step - loss: 12.3998 - accuracy: 0.5566 - val_loss: 0.6673 - val_accuracy: 0.5796

Epoch 58/900

355/355 [==============================] - 7s 19ms/step - loss: 12.4041 - accuracy: 0.5577 - val_loss: 0.6663 - val_accuracy: 0.5748

Epoch 59/900

355/355 [==============================] - 7s 19ms/step - loss: 12.4236 - accuracy: 0.5577 - val_loss: 0.6652 - val_accuracy: 0.5861

Epoch 60/900

355/355 [==============================] - 7s 19ms/step - loss: 12.4024 - accuracy: 0.5590 - val_loss: 0.6656 - val_accuracy: 0.5802

Epoch 61/900

355/355 [==============================] - 7s 19ms/step - loss: 12.3727 - accuracy: 0.5591 - val_loss: 0.6662 - val_accuracy: 0.5823

Epoch 62/900

355/355 [==============================] - 7s 19ms/step - loss: 12.3742 - accuracy: 0.5637 - val_loss: 0.6654 - val_accuracy: 0.5834

Epoch 63/900

355/355 [==============================] - 7s 19ms/step - loss: 12.3678 - accuracy: 0.5647 - val_loss: 0.6635 - val_accuracy: 0.5936

Epoch 64/900

355/355 [==============================] - 7s 19ms/step - loss: 12.3455 - accuracy: 0.5725 - val_loss: 0.6647 - val_accuracy: 0.5839

Epoch 65/900

355/355 [==============================] - 7s 19ms/step - loss: 12.3724 - accuracy: 0.5698 - val_loss: 0.6662 - val_accuracy: 0.5786

Epoch 66/900

355/355 [==============================] - 7s 19ms/step - loss: 12.3374 - accuracy: 0.5691 - val_loss: 0.6627 - val_accuracy: 0.5930

Epoch 67/900

355/355 [==============================] - 7s 19ms/step - loss: 12.4055 - accuracy: 0.5695 - val_loss: 0.6630 - val_accuracy: 0.5909

Epoch 68/900

355/355 [==============================] - 7s 19ms/step - loss: 12.3702 - accuracy: 0.5690 - val_loss: 0.6640 - val_accuracy: 0.5807

Epoch 69/900

355/355 [==============================] - 7s 19ms/step - loss: 12.2883 - accuracy: 0.5729 - val_loss: 0.6626 - val_accuracy: 0.5871

Epoch 70/900

355/355 [==============================] - 7s 19ms/step - loss: 12.3394 - accuracy: 0.5737 - val_loss: 0.6616 - val_accuracy: 0.5914

Epoch 71/900

355/355 [==============================] - 7s 19ms/step - loss: 12.3224 - accuracy: 0.5856 - val_loss: 0.6619 - val_accuracy: 0.5893

Epoch 72/900

355/355 [==============================] - 7s 19ms/step - loss: 12.2844 - accuracy: 0.5816 - val_loss: 0.6627 - val_accuracy: 0.5818

Epoch 73/900

355/355 [==============================] - 7s 19ms/step - loss: 12.2752 - accuracy: 0.5783 - val_loss: 0.6604 - val_accuracy: 0.5946

Epoch 74/900

355/355 [==============================] - 7s 19ms/step - loss: 12.2866 - accuracy: 0.5826 - val_loss: 0.6603 - val_accuracy: 0.5936

Epoch 75/900

355/355 [==============================] - 7s 19ms/step - loss: 12.2988 - accuracy: 0.5783 - val_loss: 0.6602 - val_accuracy: 0.5877

Epoch 76/900

355/355 [==============================] - 7s 19ms/step - loss: 12.2256 - accuracy: 0.5833 - val_loss: 0.6609 - val_accuracy: 0.5834

Epoch 77/900

355/355 [==============================] - 7s 19ms/step - loss: 12.2736 - accuracy: 0.5758 - val_loss: 0.6602 - val_accuracy: 0.5893

Epoch 78/900

355/355 [==============================] - 7s 19ms/step - loss: 12.2856 - accuracy: 0.5837 - val_loss: 0.6601 - val_accuracy: 0.5834

Epoch 79/900

355/355 [==============================] - 7s 19ms/step - loss: 12.2528 - accuracy: 0.5810 - val_loss: 0.6594 - val_accuracy: 0.5887

Epoch 80/900

355/355 [==============================] - 7s 19ms/step - loss: 12.2304 - accuracy: 0.5858 - val_loss: 0.6602 - val_accuracy: 0.5893

Epoch 81/900

355/355 [==============================] - 7s 19ms/step - loss: 12.2764 - accuracy: 0.5799 - val_loss: 0.6587 - val_accuracy: 0.5903

Epoch 82/900

355/355 [==============================] - 7s 19ms/step - loss: 12.2250 - accuracy: 0.5905 - val_loss: 0.6578 - val_accuracy: 0.5914

Epoch 83/900

355/355 [==============================] - 7s 19ms/step - loss: 12.1905 - accuracy: 0.5898 - val_loss: 0.6572 - val_accuracy: 0.5925

Epoch 84/900

355/355 [==============================] - 7s 19ms/step - loss: 12.2383 - accuracy: 0.5891 - val_loss: 0.6580 - val_accuracy: 0.5920

Epoch 85/900

355/355 [==============================] - 7s 19ms/step - loss: 12.1961 - accuracy: 0.5879 - val_loss: 0.6571 - val_accuracy: 0.5914

Epoch 86/900

355/355 [==============================] - 7s 19ms/step - loss: 12.2001 - accuracy: 0.5855 - val_loss: 0.6589 - val_accuracy: 0.5930

Epoch 87/900

355/355 [==============================] - 7s 19ms/step - loss: 12.2252 - accuracy: 0.5879 - val_loss: 0.6570 - val_accuracy: 0.5936

Epoch 88/900

355/355 [==============================] - 7s 19ms/step - loss: 12.1408 - accuracy: 0.5939 - val_loss: 0.6562 - val_accuracy: 0.5973

Epoch 89/900

355/355 [==============================] - 7s 19ms/step - loss: 12.1520 - accuracy: 0.5897 - val_loss: 0.6573 - val_accuracy: 0.5925

Epoch 90/900

355/355 [==============================] - 7s 19ms/step - loss: 12.1697 - accuracy: 0.5881 - val_loss: 0.6563 - val_accuracy: 0.5968

Epoch 91/900

355/355 [==============================] - 7s 19ms/step - loss: 12.1612 - accuracy: 0.5902 - val_loss: 0.6568 - val_accuracy: 0.5952

Epoch 92/900

355/355 [==============================] - 7s 19ms/step - loss: 12.1036 - accuracy: 0.5974 - val_loss: 0.6567 - val_accuracy: 0.5957

Epoch 93/900

355/355 [==============================] - 7s 19ms/step - loss: 12.1128 - accuracy: 0.5974 - val_loss: 0.6566 - val_accuracy: 0.5979

Epoch 94/900

355/355 [==============================] - 7s 19ms/step - loss: 12.1373 - accuracy: 0.5921 - val_loss: 0.6573 - val_accuracy: 0.5957

Epoch 95/900

355/355 [==============================] - 7s 19ms/step - loss: 12.1169 - accuracy: 0.6002 - val_loss: 0.6557 - val_accuracy: 0.6027

Epoch 96/900

355/355 [==============================] - 7s 19ms/step - loss: 12.0787 - accuracy: 0.5980 - val_loss: 0.6570 - val_accuracy: 0.6016

Epoch 97/900

355/355 [==============================] - 7s 19ms/step - loss: 12.0949 - accuracy: 0.5956 - val_loss: 0.6561 - val_accuracy: 0.5984

Epoch 98/900

355/355 [==============================] - 7s 19ms/step - loss: 12.1881 - accuracy: 0.5918 - val_loss: 0.6552 - val_accuracy: 0.6027

Epoch 99/900

355/355 [==============================] - 7s 19ms/step - loss: 12.0806 - accuracy: 0.5955 - val_loss: 0.6568 - val_accuracy: 0.6021

Epoch 100/900

355/355 [==============================] - 7s 19ms/step - loss: 12.1233 - accuracy: 0.5944 - val_loss: 0.6563 - val_accuracy: 0.5946

Epoch 101/900

355/355 [==============================] - 7s 19ms/step - loss: 12.0763 - accuracy: 0.5968 - val_loss: 0.6548 - val_accuracy: 0.6005

Epoch 102/900

355/355 [==============================] - 7s 19ms/step - loss: 12.0658 - accuracy: 0.5967 - val_loss: 0.6557 - val_accuracy: 0.6032

Epoch 103/900

355/355 [==============================] - 7s 19ms/step - loss: 11.9966 - accuracy: 0.6053 - val_loss: 0.6562 - val_accuracy: 0.6086

Epoch 104/900

355/355 [==============================] - 7s 19ms/step - loss: 12.0338 - accuracy: 0.6023 - val_loss: 0.6553 - val_accuracy: 0.6097

Epoch 105/900

355/355 [==============================] - 7s 19ms/step - loss: 12.0017 - accuracy: 0.6035 - val_loss: 0.6544 - val_accuracy: 0.6048

Epoch 106/900

355/355 [==============================] - 7s 19ms/step - loss: 12.0690 - accuracy: 0.5987 - val_loss: 0.6534 - val_accuracy: 0.6091

Epoch 107/900

355/355 [==============================] - 7s 19ms/step - loss: 12.0204 - accuracy: 0.6037 - val_loss: 0.6529 - val_accuracy: 0.6091

Epoch 108/900

355/355 [==============================] - 7s 19ms/step - loss: 12.0473 - accuracy: 0.6002 - val_loss: 0.6534 - val_accuracy: 0.6102

Epoch 109/900

355/355 [==============================] - 7s 19ms/step - loss: 12.0526 - accuracy: 0.6007 - val_loss: 0.6547 - val_accuracy: 0.6097

Epoch 110/900

355/355 [==============================] - 7s 19ms/step - loss: 12.0821 - accuracy: 0.6001 - val_loss: 0.6536 - val_accuracy: 0.6091

Epoch 111/900

355/355 [==============================] - 7s 19ms/step - loss: 12.0131 - accuracy: 0.6054 - val_loss: 0.6525 - val_accuracy: 0.6145

Epoch 112/900

355/355 [==============================] - 7s 19ms/step - loss: 12.0046 - accuracy: 0.6057 - val_loss: 0.6583 - val_accuracy: 0.6097

Epoch 113/900

355/355 [==============================] - 7s 19ms/step - loss: 12.0244 - accuracy: 0.6063 - val_loss: 0.6529 - val_accuracy: 0.6097

Epoch 114/900

355/355 [==============================] - 7s 19ms/step - loss: 12.0294 - accuracy: 0.6018 - val_loss: 0.6513 - val_accuracy: 0.6134

Epoch 115/900

355/355 [==============================] - 7s 19ms/step - loss: 12.0083 - accuracy: 0.6046 - val_loss: 0.6565 - val_accuracy: 0.6102

Epoch 116/900

355/355 [==============================] - 7s 19ms/step - loss: 12.0219 - accuracy: 0.6060 - val_loss: 0.6566 - val_accuracy: 0.6097

Epoch 117/900

355/355 [==============================] - 7s 19ms/step - loss: 11.9554 - accuracy: 0.6047 - val_loss: 0.6543 - val_accuracy: 0.6091

Epoch 118/900

355/355 [==============================] - 7s 19ms/step - loss: 11.9544 - accuracy: 0.6063 - val_loss: 0.6543 - val_accuracy: 0.6129

Epoch 119/900

355/355 [==============================] - 7s 19ms/step - loss: 11.9723 - accuracy: 0.6022 - val_loss: 0.6584 - val_accuracy: 0.6075

Epoch 120/900

355/355 [==============================] - 7s 19ms/step - loss: 12.0099 - accuracy: 0.6005 - val_loss: 0.6555 - val_accuracy: 0.6113

Epoch 121/900

355/355 [==============================] - 7s 19ms/step - loss: 11.9829 - accuracy: 0.6099 - val_loss: 0.6585 - val_accuracy: 0.6075

Epoch 122/900

355/355 [==============================] - 7s 19ms/step - loss: 12.0087 - accuracy: 0.6046 - val_loss: 0.6523 - val_accuracy: 0.6166

Epoch 123/900

355/355 [==============================] - 7s 19ms/step - loss: 12.0157 - accuracy: 0.6074 - val_loss: 0.6521 - val_accuracy: 0.6166

Epoch 124/900

355/355 [==============================] - 7s 19ms/step - loss: 11.9641 - accuracy: 0.6058 - val_loss: 0.6610 - val_accuracy: 0.6027

Epoch 125/900

355/355 [==============================] - 7s 19ms/step - loss: 11.9565 - accuracy: 0.6029 - val_loss: 0.6575 - val_accuracy: 0.6054

Epoch 126/900

355/355 [==============================] - 7s 19ms/step - loss: 11.9441 - accuracy: 0.6066 - val_loss: 0.6540 - val_accuracy: 0.6139

Epoch 127/900

355/355 [==============================] - 7s 19ms/step - loss: 11.9695 - accuracy: 0.6089 - val_loss: 0.6539 - val_accuracy: 0.6113

Epoch 128/900

355/355 [==============================] - 7s 19ms/step - loss: 11.9707 - accuracy: 0.6052 - val_loss: 0.6562 - val_accuracy: 0.6134

Epoch 129/900

355/355 [==============================] - 7s 19ms/step - loss: 11.9040 - accuracy: 0.6142 - val_loss: 0.6541 - val_accuracy: 0.6139

Epoch 130/900

355/355 [==============================] - 7s 19ms/step - loss: 11.9127 - accuracy: 0.6112 - val_loss: 0.6548 - val_accuracy: 0.6134

Epoch 131/900

355/355 [==============================] - 7s 19ms/step - loss: 11.8418 - accuracy: 0.6172 - val_loss: 0.6576 - val_accuracy: 0.6086

Epoch 132/900

355/355 [==============================] - 7s 19ms/step - loss: 11.9357 - accuracy: 0.6065 - val_loss: 0.6516 - val_accuracy: 0.6139

Epoch 133/900

355/355 [==============================] - 7s 19ms/step - loss: 11.9109 - accuracy: 0.6125 - val_loss: 0.6538 - val_accuracy: 0.6139

Epoch 134/900

355/355 [==============================] - 7s 19ms/step - loss: 11.8828 - accuracy: 0.6106 - val_loss: 0.6550 - val_accuracy: 0.6134

Epoch 135/900

355/355 [==============================] - 7s 19ms/step - loss: 11.8794 - accuracy: 0.6134 - val_loss: 0.6511 - val_accuracy: 0.6129

Epoch 136/900

355/355 [==============================] - 7s 19ms/step - loss: 11.9427 - accuracy: 0.6096 - val_loss: 0.6581 - val_accuracy: 0.6091

Epoch 137/900

355/355 [==============================] - 7s 19ms/step - loss: 11.9270 - accuracy: 0.6110 - val_loss: 0.6570 - val_accuracy: 0.6054

Epoch 138/900

355/355 [==============================] - 7s 19ms/step - loss: 11.8710 - accuracy: 0.6094 - val_loss: 0.6497 - val_accuracy: 0.6177

Epoch 139/900

355/355 [==============================] - 7s 19ms/step - loss: 11.8723 - accuracy: 0.6132 - val_loss: 0.6532 - val_accuracy: 0.6166

Epoch 140/900

355/355 [==============================] - 7s 19ms/step - loss: 11.9087 - accuracy: 0.6081 - val_loss: 0.6555 - val_accuracy: 0.6102

Epoch 141/900

355/355 [==============================] - 7s 19ms/step - loss: 11.9365 - accuracy: 0.6121 - val_loss: 0.6545 - val_accuracy: 0.6134

Epoch 142/900

355/355 [==============================] - 7s 19ms/step - loss: 11.9061 - accuracy: 0.6096 - val_loss: 0.6557 - val_accuracy: 0.6102

Epoch 143/900

355/355 [==============================] - 7s 19ms/step - loss: 11.9250 - accuracy: 0.6090 - val_loss: 0.6526 - val_accuracy: 0.6161

Epoch 144/900

355/355 [==============================] - 7s 19ms/step - loss: 11.8732 - accuracy: 0.6089 - val_loss: 0.6577 - val_accuracy: 0.6075

Epoch 145/900

355/355 [==============================] - 7s 19ms/step - loss: 11.8620 - accuracy: 0.6139 - val_loss: 0.6588 - val_accuracy: 0.6043

Epoch 146/900

355/355 [==============================] - 7s 19ms/step - loss: 11.8953 - accuracy: 0.6127 - val_loss: 0.6573 - val_accuracy: 0.6091

Epoch 147/900

355/355 [==============================] - 7s 19ms/step - loss: 11.8384 - accuracy: 0.6143 - val_loss: 0.6589 - val_accuracy: 0.6064

Epoch 148/900

355/355 [==============================] - 7s 19ms/step - loss: 11.8121 - accuracy: 0.6198 - val_loss: 0.6515 - val_accuracy: 0.6113

Epoch 149/900

355/355 [==============================] - 7s 19ms/step - loss: 11.8634 - accuracy: 0.6097 - val_loss: 0.6546 - val_accuracy: 0.6118

Epoch 150/900

355/355 [==============================] - 7s 19ms/step - loss: 11.8584 - accuracy: 0.6176 - val_loss: 0.6550 - val_accuracy: 0.6107

Epoch 151/900

355/355 [==============================] - 7s 19ms/step - loss: 11.8432 - accuracy: 0.6123 - val_loss: 0.6541 - val_accuracy: 0.6097

Epoch 152/900

355/355 [==============================] - 7s 19ms/step - loss: 11.8695 - accuracy: 0.6146 - val_loss: 0.6573 - val_accuracy: 0.6043

Epoch 153/900

355/355 [==============================] - 7s 19ms/step - loss: 11.8568 - accuracy: 0.6127 - val_loss: 0.6523 - val_accuracy: 0.6118

Epoch 154/900

355/355 [==============================] - 7s 19ms/step - loss: 11.8318 - accuracy: 0.6142 - val_loss: 0.6508 - val_accuracy: 0.6139

Epoch 155/900

355/355 [==============================] - 7s 19ms/step - loss: 11.8198 - accuracy: 0.6170 - val_loss: 0.6536 - val_accuracy: 0.6097

Epoch 156/900

355/355 [==============================] - 7s 19ms/step - loss: 11.8488 - accuracy: 0.6160 - val_loss: 0.6517 - val_accuracy: 0.6155

Epoch 157/900

355/355 [==============================] - 7s 19ms/step - loss: 11.9084 - accuracy: 0.6112 - val_loss: 0.6569 - val_accuracy: 0.6048

Epoch 158/900

355/355 [==============================] - 7s 19ms/step - loss: 11.7801 - accuracy: 0.6189 - val_loss: 0.6511 - val_accuracy: 0.6107

Epoch 159/900

355/355 [==============================] - 7s 19ms/step - loss: 11.8052 - accuracy: 0.6154 - val_loss: 0.6466 - val_accuracy: 0.6155

Epoch 160/900

355/355 [==============================] - 7s 19ms/step - loss: 11.8635 - accuracy: 0.6132 - val_loss: 0.6534 - val_accuracy: 0.6123

Epoch 161/900

355/355 [==============================] - 7s 19ms/step - loss: 11.7930 - accuracy: 0.6137 - val_loss: 0.6457 - val_accuracy: 0.6193

Epoch 162/900

355/355 [==============================] - 7s 19ms/step - loss: 11.7534 - accuracy: 0.6199 - val_loss: 0.6545 - val_accuracy: 0.6145

Epoch 163/900

355/355 [==============================] - 7s 19ms/step - loss: 11.8520 - accuracy: 0.6100 - val_loss: 0.6523 - val_accuracy: 0.6161

Epoch 164/900

355/355 [==============================] - 7s 19ms/step - loss: 11.7738 - accuracy: 0.6200 - val_loss: 0.6482 - val_accuracy: 0.6172

Epoch 165/900

355/355 [==============================] - 7s 19ms/step - loss: 11.8262 - accuracy: 0.6159 - val_loss: 0.6616 - val_accuracy: 0.6043

Epoch 166/900

355/355 [==============================] - 7s 19ms/step - loss: 11.8110 - accuracy: 0.6217 - val_loss: 0.6522 - val_accuracy: 0.6123

Epoch 167/900

355/355 [==============================] - 7s 19ms/step - loss: 11.8200 - accuracy: 0.6177 - val_loss: 0.6518 - val_accuracy: 0.6134

Epoch 168/900

355/355 [==============================] - 7s 19ms/step - loss: 11.7605 - accuracy: 0.6210 - val_loss: 0.6501 - val_accuracy: 0.6118

Epoch 169/900

355/355 [==============================] - 7s 19ms/step - loss: 11.7875 - accuracy: 0.6191 - val_loss: 0.6548 - val_accuracy: 0.6123

Epoch 170/900

355/355 [==============================] - 7s 19ms/step - loss: 11.7346 - accuracy: 0.6215 - val_loss: 0.6558 - val_accuracy: 0.6059

Epoch 171/900

355/355 [==============================] - 7s 19ms/step - loss: 11.7632 - accuracy: 0.6224 - val_loss: 0.6532 - val_accuracy: 0.6123

Epoch 172/900

355/355 [==============================] - 7s 19ms/step - loss: 11.8000 - accuracy: 0.6159 - val_loss: 0.6645 - val_accuracy: 0.5995

Epoch 173/900

355/355 [==============================] - 7s 19ms/step - loss: 11.8135 - accuracy: 0.6143 - val_loss: 0.6475 - val_accuracy: 0.6150

Epoch 174/900

355/355 [==============================] - 7s 19ms/step - loss: 11.8009 - accuracy: 0.6159 - val_loss: 0.6500 - val_accuracy: 0.6139

Epoch 175/900

355/355 [==============================] - 7s 19ms/step - loss: 11.7688 - accuracy: 0.6167 - val_loss: 0.6586 - val_accuracy: 0.6038

Epoch 176/900

355/355 [==============================] - 7s 19ms/step - loss: 11.7680 - accuracy: 0.6206 - val_loss: 0.6560 - val_accuracy: 0.6075

Epoch 177/900

355/355 [==============================] - 7s 19ms/step - loss: 11.7119 - accuracy: 0.6221 - val_loss: 0.6502 - val_accuracy: 0.6086

Epoch 178/900

355/355 [==============================] - 7s 19ms/step - loss: 11.8054 - accuracy: 0.6156 - val_loss: 0.6580 - val_accuracy: 0.6043

Epoch 179/900

355/355 [==============================] - 7s 19ms/step - loss: 11.7714 - accuracy: 0.6160 - val_loss: 0.6635 - val_accuracy: 0.6000

Epoch 180/900

355/355 [==============================] - 7s 19ms/step - loss: 11.7818 - accuracy: 0.6199 - val_loss: 0.6535 - val_accuracy: 0.6102

Epoch 181/900

355/355 [==============================] - 7s 19ms/step - loss: 11.8025 - accuracy: 0.6143 - val_loss: 0.6450 - val_accuracy: 0.6161

Epoch 182/900

355/355 [==============================] - 7s 19ms/step - loss: 11.6851 - accuracy: 0.6244 - val_loss: 0.6514 - val_accuracy: 0.6097

Epoch 183/900

355/355 [==============================] - 7s 19ms/step - loss: 11.7859 - accuracy: 0.6142 - val_loss: 0.6492 - val_accuracy: 0.6107

Epoch 184/900

355/355 [==============================] - 7s 19ms/step - loss: 11.7599 - accuracy: 0.6191 - val_loss: 0.6511 - val_accuracy: 0.6107

Epoch 185/900

355/355 [==============================] - 7s 19ms/step - loss: 11.7370 - accuracy: 0.6206 - val_loss: 0.6508 - val_accuracy: 0.6064

Epoch 186/900

355/355 [==============================] - 7s 19ms/step - loss: 11.7287 - accuracy: 0.6250 - val_loss: 0.6555 - val_accuracy: 0.6027

Epoch 187/900

355/355 [==============================] - 7s 19ms/step - loss: 11.6935 - accuracy: 0.6225 - val_loss: 0.6425 - val_accuracy: 0.6214

Epoch 188/900

355/355 [==============================] - 7s 19ms/step - loss: 11.6742 - accuracy: 0.6256 - val_loss: 0.6485 - val_accuracy: 0.6155

Epoch 189/900

355/355 [==============================] - 7s 19ms/step - loss: 11.7191 - accuracy: 0.6201 - val_loss: 0.6453 - val_accuracy: 0.6177

Epoch 190/900

355/355 [==============================] - 7s 19ms/step - loss: 11.7345 - accuracy: 0.6251 - val_loss: 0.6439 - val_accuracy: 0.6252

Epoch 191/900

355/355 [==============================] - 7s 19ms/step - loss: 11.7574 - accuracy: 0.6220 - val_loss: 0.6473 - val_accuracy: 0.6134

Epoch 192/900

355/355 [==============================] - 7s 19ms/step - loss: 11.6989 - accuracy: 0.6232 - val_loss: 0.6429 - val_accuracy: 0.6220

Epoch 193/900

355/355 [==============================] - 7s 19ms/step - loss: 11.6607 - accuracy: 0.6258 - val_loss: 0.6436 - val_accuracy: 0.6193

Epoch 194/900

355/355 [==============================] - 7s 19ms/step - loss: 11.7344 - accuracy: 0.6195 - val_loss: 0.6490 - val_accuracy: 0.6113

Epoch 195/900

355/355 [==============================] - 7s 19ms/step - loss: 11.6507 - accuracy: 0.6245 - val_loss: 0.6412 - val_accuracy: 0.6204

Epoch 196/900

355/355 [==============================] - 7s 19ms/step - loss: 11.7524 - accuracy: 0.6192 - val_loss: 0.6490 - val_accuracy: 0.6086

Epoch 197/900

355/355 [==============================] - 7s 19ms/step - loss: 11.6457 - accuracy: 0.6298 - val_loss: 0.6413 - val_accuracy: 0.6214

Epoch 198/900

355/355 [==============================] - 7s 19ms/step - loss: 11.6637 - accuracy: 0.6240 - val_loss: 0.6489 - val_accuracy: 0.6118

Epoch 199/900

355/355 [==============================] - 7s 19ms/step - loss: 11.6695 - accuracy: 0.6215 - val_loss: 0.6445 - val_accuracy: 0.6188

Epoch 200/900

355/355 [==============================] - 7s 19ms/step - loss: 11.6476 - accuracy: 0.6256 - val_loss: 0.6459 - val_accuracy: 0.6150

Epoch 201/900

355/355 [==============================] - 7s 19ms/step - loss: 11.6225 - accuracy: 0.6306 - val_loss: 0.6475 - val_accuracy: 0.6129

Epoch 202/900

355/355 [==============================] - 7s 19ms/step - loss: 11.6691 - accuracy: 0.6242 - val_loss: 0.6500 - val_accuracy: 0.6113

Epoch 203/900

355/355 [==============================] - 7s 19ms/step - loss: 11.6839 - accuracy: 0.6234 - val_loss: 0.6474 - val_accuracy: 0.6139

Epoch 204/900

355/355 [==============================] - 7s 19ms/step - loss: 11.6473 - accuracy: 0.6271 - val_loss: 0.6490 - val_accuracy: 0.6097

Epoch 205/900

355/355 [==============================] - 7s 19ms/step - loss: 11.6789 - accuracy: 0.6230 - val_loss: 0.6398 - val_accuracy: 0.6225

Epoch 206/900

355/355 [==============================] - 7s 19ms/step - loss: 11.6837 - accuracy: 0.6198 - val_loss: 0.6483 - val_accuracy: 0.6150

Epoch 207/900

355/355 [==============================] - 7s 19ms/step - loss: 11.6542 - accuracy: 0.6297 - val_loss: 0.6481 - val_accuracy: 0.6139

Epoch 208/900

355/355 [==============================] - 7s 19ms/step - loss: 11.6342 - accuracy: 0.6260 - val_loss: 0.6516 - val_accuracy: 0.6059

Epoch 209/900

355/355 [==============================] - 7s 19ms/step - loss: 11.6388 - accuracy: 0.6256 - val_loss: 0.6416 - val_accuracy: 0.6204

Epoch 210/900

355/355 [==============================] - 7s 19ms/step - loss: 11.6204 - accuracy: 0.6242 - val_loss: 0.6396 - val_accuracy: 0.6306

Epoch 211/900

355/355 [==============================] - 7s 19ms/step - loss: 11.6504 - accuracy: 0.6230 - val_loss: 0.6434 - val_accuracy: 0.6241

Epoch 212/900

355/355 [==============================] - 7s 19ms/step - loss: 11.6805 - accuracy: 0.6204 - val_loss: 0.6532 - val_accuracy: 0.6139

Epoch 213/900

355/355 [==============================] - 7s 19ms/step - loss: 11.6528 - accuracy: 0.6262 - val_loss: 0.6444 - val_accuracy: 0.6209

Epoch 214/900

355/355 [==============================] - 7s 19ms/step - loss: 11.6497 - accuracy: 0.6282 - val_loss: 0.6410 - val_accuracy: 0.6273

Epoch 215/900

355/355 [==============================] - 7s 19ms/step - loss: 11.6378 - accuracy: 0.6261 - val_loss: 0.6469 - val_accuracy: 0.6155

Epoch 216/900

355/355 [==============================] - 7s 19ms/step - loss: 11.6165 - accuracy: 0.6298 - val_loss: 0.6492 - val_accuracy: 0.6134

Epoch 217/900

355/355 [==============================] - 7s 19ms/step - loss: 11.5876 - accuracy: 0.6331 - val_loss: 0.6481 - val_accuracy: 0.6129

Epoch 218/900

355/355 [==============================] - 7s 19ms/step - loss: 11.6432 - accuracy: 0.6284 - val_loss: 0.6447 - val_accuracy: 0.6198

Epoch 219/900

355/355 [==============================] - 7s 19ms/step - loss: 11.6029 - accuracy: 0.6285 - val_loss: 0.6490 - val_accuracy: 0.6150

Epoch 220/900

355/355 [==============================] - 7s 19ms/step - loss: 11.6245 - accuracy: 0.6269 - val_loss: 0.6414 - val_accuracy: 0.6263

Epoch 221/900

355/355 [==============================] - 7s 19ms/step - loss: 11.5666 - accuracy: 0.6298 - val_loss: 0.6445 - val_accuracy: 0.6225

Epoch 222/900

355/355 [==============================] - 7s 19ms/step - loss: 11.6014 - accuracy: 0.6311 - val_loss: 0.6436 - val_accuracy: 0.6247

Epoch 223/900

355/355 [==============================] - 7s 19ms/step - loss: 11.6121 - accuracy: 0.6254 - val_loss: 0.6447 - val_accuracy: 0.6188

Epoch 224/900

355/355 [==============================] - 7s 19ms/step - loss: 11.5895 - accuracy: 0.6298 - val_loss: 0.6507 - val_accuracy: 0.6177

Epoch 225/900

355/355 [==============================] - 7s 19ms/step - loss: 11.5980 - accuracy: 0.6262 - val_loss: 0.6470 - val_accuracy: 0.6198

Epoch 226/900

355/355 [==============================] - 7s 19ms/step - loss: 11.6103 - accuracy: 0.6283 - val_loss: 0.6416 - val_accuracy: 0.6231

Epoch 227/900

355/355 [==============================] - 7s 19ms/step - loss: 11.5928 - accuracy: 0.6262 - val_loss: 0.6465 - val_accuracy: 0.6166

Epoch 228/900

355/355 [==============================] - 7s 19ms/step - loss: 11.6017 - accuracy: 0.6285 - val_loss: 0.6404 - val_accuracy: 0.6306

Epoch 229/900

355/355 [==============================] - 7s 19ms/step - loss: 11.5032 - accuracy: 0.6344 - val_loss: 0.6407 - val_accuracy: 0.6300

Epoch 230/900

355/355 [==============================] - 7s 19ms/step - loss: 11.5801 - accuracy: 0.6302 - val_loss: 0.6541 - val_accuracy: 0.6113

Epoch 231/900

355/355 [==============================] - 7s 19ms/step - loss: 11.5617 - accuracy: 0.6327 - val_loss: 0.6422 - val_accuracy: 0.6209

Epoch 232/900

355/355 [==============================] - 7s 19ms/step - loss: 11.6484 - accuracy: 0.6236 - val_loss: 0.6432 - val_accuracy: 0.6252

Epoch 233/900

355/355 [==============================] - 7s 19ms/step - loss: 11.5875 - accuracy: 0.6265 - val_loss: 0.6425 - val_accuracy: 0.6225

Epoch 234/900

355/355 [==============================] - 7s 19ms/step - loss: 11.5469 - accuracy: 0.6325 - val_loss: 0.6465 - val_accuracy: 0.6182

Epoch 235/900

355/355 [==============================] - 7s 19ms/step - loss: 11.5487 - accuracy: 0.6337 - val_loss: 0.6403 - val_accuracy: 0.6257

Epoch 236/900

355/355 [==============================] - 7s 19ms/step - loss: 11.5415 - accuracy: 0.6319 - val_loss: 0.6399 - val_accuracy: 0.6295

Epoch 237/900

355/355 [==============================] - 7s 19ms/step - loss: 11.6031 - accuracy: 0.6297 - val_loss: 0.6401 - val_accuracy: 0.6247

Epoch 238/900

355/355 [==============================] - 7s 19ms/step - loss: 11.5222 - accuracy: 0.6306 - val_loss: 0.6447 - val_accuracy: 0.6188

Epoch 239/900

355/355 [==============================] - 7s 19ms/step - loss: 11.5171 - accuracy: 0.6336 - val_loss: 0.6405 - val_accuracy: 0.6198

Epoch 240/900

355/355 [==============================] - 7s 19ms/step - loss: 11.4918 - accuracy: 0.6342 - val_loss: 0.6528 - val_accuracy: 0.6107

Epoch 241/900

355/355 [==============================] - 7s 19ms/step - loss: 11.5186 - accuracy: 0.6343 - val_loss: 0.6405 - val_accuracy: 0.6252

Epoch 242/900

355/355 [==============================] - 7s 19ms/step - loss: 11.4975 - accuracy: 0.6324 - val_loss: 0.6358 - val_accuracy: 0.6273

Epoch 243/900

355/355 [==============================] - 7s 19ms/step - loss: 11.5482 - accuracy: 0.6264 - val_loss: 0.6475 - val_accuracy: 0.6177

Epoch 244/900

355/355 [==============================] - 7s 19ms/step - loss: 11.5132 - accuracy: 0.6364 - val_loss: 0.6552 - val_accuracy: 0.6086

Epoch 245/900

355/355 [==============================] - 7s 19ms/step - loss: 11.5039 - accuracy: 0.6340 - val_loss: 0.6471 - val_accuracy: 0.6188

Epoch 246/900

355/355 [==============================] - 7s 19ms/step - loss: 11.5689 - accuracy: 0.6258 - val_loss: 0.6405 - val_accuracy: 0.6241

Epoch 247/900

355/355 [==============================] - 7s 19ms/step - loss: 11.4943 - accuracy: 0.6378 - val_loss: 0.6356 - val_accuracy: 0.6311

Epoch 248/900

355/355 [==============================] - 7s 19ms/step - loss: 11.5547 - accuracy: 0.6306 - val_loss: 0.6420 - val_accuracy: 0.6214

Epoch 249/900

355/355 [==============================] - 7s 19ms/step - loss: 11.5067 - accuracy: 0.6308 - val_loss: 0.6399 - val_accuracy: 0.6209

Epoch 250/900

355/355 [==============================] - 7s 19ms/step - loss: 11.5534 - accuracy: 0.6336 - val_loss: 0.6381 - val_accuracy: 0.6252

Epoch 251/900

355/355 [==============================] - 7s 19ms/step - loss: 11.5197 - accuracy: 0.6329 - val_loss: 0.6461 - val_accuracy: 0.6198

Epoch 252/900

355/355 [==============================] - 7s 19ms/step - loss: 11.5166 - accuracy: 0.6328 - val_loss: 0.6425 - val_accuracy: 0.6220

Epoch 253/900

355/355 [==============================] - 7s 19ms/step - loss: 11.5424 - accuracy: 0.6283 - val_loss: 0.6464 - val_accuracy: 0.6214

Epoch 254/900

355/355 [==============================] - 7s 19ms/step - loss: 11.4649 - accuracy: 0.6357 - val_loss: 0.6391 - val_accuracy: 0.6214

Epoch 255/900

355/355 [==============================] - 7s 19ms/step - loss: 11.5320 - accuracy: 0.6343 - val_loss: 0.6374 - val_accuracy: 0.6252

Epoch 256/900

355/355 [==============================] - 7s 19ms/step - loss: 11.4889 - accuracy: 0.6370 - val_loss: 0.6368 - val_accuracy: 0.6273

Epoch 257/900

355/355 [==============================] - 7s 19ms/step - loss: 11.5498 - accuracy: 0.6282 - val_loss: 0.6357 - val_accuracy: 0.6268

Epoch 258/900

355/355 [==============================] - 7s 19ms/step - loss: 11.4600 - accuracy: 0.6343 - val_loss: 0.6396 - val_accuracy: 0.6236

Epoch 259/900

355/355 [==============================] - 7s 19ms/step - loss: 11.5138 - accuracy: 0.6394 - val_loss: 0.6400 - val_accuracy: 0.6225

Epoch 260/900

355/355 [==============================] - 7s 19ms/step - loss: 11.4912 - accuracy: 0.6335 - val_loss: 0.6414 - val_accuracy: 0.6263

Epoch 261/900

355/355 [==============================] - 7s 19ms/step - loss: 11.5063 - accuracy: 0.6338 - val_loss: 0.6462 - val_accuracy: 0.6220

Epoch 262/900

355/355 [==============================] - 7s 19ms/step - loss: 11.4319 - accuracy: 0.6385 - val_loss: 0.6412 - val_accuracy: 0.6268

Epoch 263/900

355/355 [==============================] - 7s 19ms/step - loss: 11.5073 - accuracy: 0.6367 - val_loss: 0.6375 - val_accuracy: 0.6279

Epoch 264/900

355/355 [==============================] - 7s 19ms/step - loss: 11.4493 - accuracy: 0.6399 - val_loss: 0.6392 - val_accuracy: 0.6284

Epoch 265/900

355/355 [==============================] - 7s 19ms/step - loss: 11.5399 - accuracy: 0.6297 - val_loss: 0.6449 - val_accuracy: 0.6182

Epoch 266/900

355/355 [==============================] - 7s 19ms/step - loss: 11.4497 - accuracy: 0.6365 - val_loss: 0.6445 - val_accuracy: 0.6225

Epoch 267/900

355/355 [==============================] - 7s 19ms/step - loss: 11.4765 - accuracy: 0.6370 - val_loss: 0.6348 - val_accuracy: 0.6284

Epoch 268/900

355/355 [==============================] - 7s 19ms/step - loss: 11.5236 - accuracy: 0.6375 - val_loss: 0.6410 - val_accuracy: 0.6241

Epoch 269/900

355/355 [==============================] - 7s 19ms/step - loss: 11.4376 - accuracy: 0.6358 - val_loss: 0.6397 - val_accuracy: 0.6214

Epoch 270/900

355/355 [==============================] - 7s 19ms/step - loss: 11.3965 - accuracy: 0.6425 - val_loss: 0.6339 - val_accuracy: 0.6252

Epoch 271/900

355/355 [==============================] - 7s 19ms/step - loss: 11.4492 - accuracy: 0.6392 - val_loss: 0.6318 - val_accuracy: 0.6354

Epoch 272/900

355/355 [==============================] - 7s 19ms/step - loss: 11.5210 - accuracy: 0.6317 - val_loss: 0.6386 - val_accuracy: 0.6214

Epoch 273/900

355/355 [==============================] - 7s 19ms/step - loss: 11.4945 - accuracy: 0.6362 - val_loss: 0.6362 - val_accuracy: 0.6273

Epoch 274/900

355/355 [==============================] - 7s 19ms/step - loss: 11.4293 - accuracy: 0.6352 - val_loss: 0.6419 - val_accuracy: 0.6220

Epoch 275/900

355/355 [==============================] - 7s 19ms/step - loss: 11.4729 - accuracy: 0.6381 - val_loss: 0.6403 - val_accuracy: 0.6220

Epoch 276/900

355/355 [==============================] - 7s 19ms/step - loss: 11.4696 - accuracy: 0.6314 - val_loss: 0.6448 - val_accuracy: 0.6188

Epoch 277/900

355/355 [==============================] - 7s 19ms/step - loss: 11.4420 - accuracy: 0.6398 - val_loss: 0.6467 - val_accuracy: 0.6225

Epoch 278/900

355/355 [==============================] - 7s 19ms/step - loss: 11.3619 - accuracy: 0.6396 - val_loss: 0.6346 - val_accuracy: 0.6257

Epoch 279/900

355/355 [==============================] - 7s 19ms/step - loss: 11.3954 - accuracy: 0.6414 - val_loss: 0.6406 - val_accuracy: 0.6257

Epoch 280/900

355/355 [==============================] - 7s 19ms/step - loss: 11.4074 - accuracy: 0.6379 - val_loss: 0.6430 - val_accuracy: 0.6198

Epoch 281/900

355/355 [==============================] - 7s 19ms/step - loss: 11.3988 - accuracy: 0.6428 - val_loss: 0.6344 - val_accuracy: 0.6295

Epoch 282/900

355/355 [==============================] - 7s 19ms/step - loss: 11.4199 - accuracy: 0.6388 - val_loss: 0.6330 - val_accuracy: 0.6338

Epoch 283/900

355/355 [==============================] - 7s 19ms/step - loss: 11.3920 - accuracy: 0.6408 - val_loss: 0.6370 - val_accuracy: 0.6284

Epoch 284/900

355/355 [==============================] - 7s 19ms/step - loss: 11.4178 - accuracy: 0.6425 - val_loss: 0.6404 - val_accuracy: 0.6268

Epoch 285/900

355/355 [==============================] - 7s 19ms/step - loss: 11.3903 - accuracy: 0.6436 - val_loss: 0.6351 - val_accuracy: 0.6295

Epoch 286/900

355/355 [==============================] - 7s 19ms/step - loss: 11.4129 - accuracy: 0.6401 - val_loss: 0.6406 - val_accuracy: 0.6273

Epoch 287/900

355/355 [==============================] - 7s 19ms/step - loss: 11.3309 - accuracy: 0.6436 - val_loss: 0.6364 - val_accuracy: 0.6252

Epoch 288/900

355/355 [==============================] - 7s 19ms/step - loss: 11.3518 - accuracy: 0.6430 - val_loss: 0.6380 - val_accuracy: 0.6241

Epoch 289/900

355/355 [==============================] - 7s 19ms/step - loss: 11.4501 - accuracy: 0.6358 - val_loss: 0.6413 - val_accuracy: 0.6241

Epoch 290/900

355/355 [==============================] - 7s 19ms/step - loss: 11.3744 - accuracy: 0.6412 - val_loss: 0.6374 - val_accuracy: 0.6241

Epoch 291/900

355/355 [==============================] - 7s 19ms/step - loss: 11.3078 - accuracy: 0.6465 - val_loss: 0.6427 - val_accuracy: 0.6150

Epoch 292/900

355/355 [==============================] - 7s 19ms/step - loss: 11.3721 - accuracy: 0.6375 - val_loss: 0.6371 - val_accuracy: 0.6247

Epoch 293/900

355/355 [==============================] - 7s 19ms/step - loss: 11.3774 - accuracy: 0.6419 - val_loss: 0.6559 - val_accuracy: 0.6204

Epoch 294/900

355/355 [==============================] - 7s 19ms/step - loss: 11.3297 - accuracy: 0.6439 - val_loss: 0.6368 - val_accuracy: 0.6225

Epoch 295/900

355/355 [==============================] - 7s 19ms/step - loss: 11.3942 - accuracy: 0.6385 - val_loss: 0.6376 - val_accuracy: 0.6241

Epoch 296/900

355/355 [==============================] - 7s 19ms/step - loss: 11.4139 - accuracy: 0.6385 - val_loss: 0.6388 - val_accuracy: 0.6236

Epoch 297/900

355/355 [==============================] - 7s 19ms/step - loss: 11.3834 - accuracy: 0.6388 - val_loss: 0.6285 - val_accuracy: 0.6375

Epoch 298/900

355/355 [==============================] - 7s 19ms/step - loss: 11.3832 - accuracy: 0.6377 - val_loss: 0.6345 - val_accuracy: 0.6311

Epoch 299/900

355/355 [==============================] - 7s 19ms/step - loss: 11.3043 - accuracy: 0.6428 - val_loss: 0.6348 - val_accuracy: 0.6338

Epoch 300/900

355/355 [==============================] - 7s 19ms/step - loss: 11.3171 - accuracy: 0.6447 - val_loss: 0.6384 - val_accuracy: 0.6257

Epoch 301/900

355/355 [==============================] - 7s 19ms/step - loss: 11.3018 - accuracy: 0.6445 - val_loss: 0.6341 - val_accuracy: 0.6359

Epoch 302/900

355/355 [==============================] - 7s 19ms/step - loss: 11.3360 - accuracy: 0.6409 - val_loss: 0.6358 - val_accuracy: 0.6311

Epoch 303/900

355/355 [==============================] - 7s 19ms/step - loss: 11.3083 - accuracy: 0.6430 - val_loss: 0.6324 - val_accuracy: 0.6365

Epoch 304/900

355/355 [==============================] - 7s 19ms/step - loss: 11.3663 - accuracy: 0.6387 - val_loss: 0.6387 - val_accuracy: 0.6300

Epoch 305/900

355/355 [==============================] - 7s 19ms/step - loss: 11.3885 - accuracy: 0.6379 - val_loss: 0.6262 - val_accuracy: 0.6391

Epoch 306/900

355/355 [==============================] - 7s 19ms/step - loss: 11.3340 - accuracy: 0.6434 - val_loss: 0.6403 - val_accuracy: 0.6263

Epoch 307/900

355/355 [==============================] - 7s 19ms/step - loss: 11.3292 - accuracy: 0.6450 - val_loss: 0.6382 - val_accuracy: 0.6290

Epoch 308/900

355/355 [==============================] - 7s 19ms/step - loss: 11.3415 - accuracy: 0.6431 - val_loss: 0.6308 - val_accuracy: 0.6316

Epoch 309/900

355/355 [==============================] - 7s 19ms/step - loss: 11.3428 - accuracy: 0.6403 - val_loss: 0.6311 - val_accuracy: 0.6284

Epoch 310/900

355/355 [==============================] - 7s 19ms/step - loss: 11.3637 - accuracy: 0.6385 - val_loss: 0.6313 - val_accuracy: 0.6338

Epoch 311/900

355/355 [==============================] - 7s 19ms/step - loss: 11.3013 - accuracy: 0.6477 - val_loss: 0.6348 - val_accuracy: 0.6332

Epoch 312/900

355/355 [==============================] - 7s 19ms/step - loss: 11.2685 - accuracy: 0.6434 - val_loss: 0.6366 - val_accuracy: 0.6322

Epoch 313/900

355/355 [==============================] - 7s 19ms/step - loss: 11.3085 - accuracy: 0.6420 - val_loss: 0.6353 - val_accuracy: 0.6338

Epoch 314/900

355/355 [==============================] - 7s 19ms/step - loss: 11.2668 - accuracy: 0.6464 - val_loss: 0.6355 - val_accuracy: 0.6311

Epoch 315/900

355/355 [==============================] - 7s 19ms/step - loss: 11.3220 - accuracy: 0.6432 - val_loss: 0.6349 - val_accuracy: 0.6370

Epoch 316/900

355/355 [==============================] - 7s 19ms/step - loss: 11.3335 - accuracy: 0.6407 - val_loss: 0.6356 - val_accuracy: 0.6343

Epoch 317/900

355/355 [==============================] - 7s 19ms/step - loss: 11.2062 - accuracy: 0.6506 - val_loss: 0.6315 - val_accuracy: 0.6365

Epoch 318/900

355/355 [==============================] - 7s 19ms/step - loss: 11.2708 - accuracy: 0.6432 - val_loss: 0.6352 - val_accuracy: 0.6375

Epoch 319/900

355/355 [==============================] - 7s 19ms/step - loss: 11.3306 - accuracy: 0.6457 - val_loss: 0.6349 - val_accuracy: 0.6381

Epoch 320/900

355/355 [==============================] - 7s 19ms/step - loss: 11.3150 - accuracy: 0.6449 - val_loss: 0.6373 - val_accuracy: 0.6343

Epoch 321/900

355/355 [==============================] - 7s 19ms/step - loss: 11.2900 - accuracy: 0.6459 - val_loss: 0.6366 - val_accuracy: 0.6365

Epoch 322/900

355/355 [==============================] - 7s 19ms/step - loss: 11.2368 - accuracy: 0.6489 - val_loss: 0.6315 - val_accuracy: 0.6386

Epoch 323/900

355/355 [==============================] - 7s 19ms/step - loss: 11.2935 - accuracy: 0.6449 - val_loss: 0.6333 - val_accuracy: 0.6391

Epoch 324/900

355/355 [==============================] - 7s 19ms/step - loss: 11.3089 - accuracy: 0.6441 - val_loss: 0.6401 - val_accuracy: 0.6284

Epoch 325/900

355/355 [==============================] - 7s 19ms/step - loss: 11.2909 - accuracy: 0.6479 - val_loss: 0.6322 - val_accuracy: 0.6397

Epoch 326/900

355/355 [==============================] - 7s 19ms/step - loss: 11.1864 - accuracy: 0.6494 - val_loss: 0.6380 - val_accuracy: 0.6375

Epoch 327/900

355/355 [==============================] - 7s 19ms/step - loss: 11.2216 - accuracy: 0.6523 - val_loss: 0.6332 - val_accuracy: 0.6370

Epoch 328/900

355/355 [==============================] - 7s 19ms/step - loss: 11.2708 - accuracy: 0.6452 - val_loss: 0.6385 - val_accuracy: 0.6316

Epoch 329/900

355/355 [==============================] - 7s 19ms/step - loss: 11.2467 - accuracy: 0.6492 - val_loss: 0.6332 - val_accuracy: 0.6365

Epoch 330/900

355/355 [==============================] - 7s 19ms/step - loss: 11.1752 - accuracy: 0.6525 - val_loss: 0.6349 - val_accuracy: 0.6354

Epoch 331/900

355/355 [==============================] - 7s 19ms/step - loss: 11.2891 - accuracy: 0.6441 - val_loss: 0.6261 - val_accuracy: 0.6343

Epoch 332/900

355/355 [==============================] - 7s 19ms/step - loss: 11.1434 - accuracy: 0.6556 - val_loss: 0.6347 - val_accuracy: 0.6327

Epoch 333/900

355/355 [==============================] - 7s 19ms/step - loss: 11.1710 - accuracy: 0.6527 - val_loss: 0.6353 - val_accuracy: 0.6316

Epoch 334/900

355/355 [==============================] - 7s 19ms/step - loss: 11.2171 - accuracy: 0.6499 - val_loss: 0.6378 - val_accuracy: 0.6338

Epoch 335/900

355/355 [==============================] - 7s 19ms/step - loss: 11.2700 - accuracy: 0.6501 - val_loss: 0.6298 - val_accuracy: 0.6450

Epoch 336/900

355/355 [==============================] - 7s 19ms/step - loss: 11.1779 - accuracy: 0.6504 - val_loss: 0.6346 - val_accuracy: 0.6375

Epoch 337/900

355/355 [==============================] - 7s 19ms/step - loss: 11.2097 - accuracy: 0.6543 - val_loss: 0.6394 - val_accuracy: 0.6311

Epoch 338/900

355/355 [==============================] - 7s 19ms/step - loss: 11.2996 - accuracy: 0.6455 - val_loss: 0.6313 - val_accuracy: 0.6397

Epoch 339/900

355/355 [==============================] - 7s 19ms/step - loss: 11.2501 - accuracy: 0.6514 - val_loss: 0.6298 - val_accuracy: 0.6370

Epoch 340/900

355/355 [==============================] - 7s 19ms/step - loss: 11.1873 - accuracy: 0.6509 - val_loss: 0.6287 - val_accuracy: 0.6413

Epoch 341/900

355/355 [==============================] - 7s 19ms/step - loss: 11.2374 - accuracy: 0.6498 - val_loss: 0.6256 - val_accuracy: 0.6381

Epoch 342/900

355/355 [==============================] - 7s 19ms/step - loss: 11.2086 - accuracy: 0.6526 - val_loss: 0.6289 - val_accuracy: 0.6386

Epoch 343/900

355/355 [==============================] - 7s 19ms/step - loss: 11.1609 - accuracy: 0.6494 - val_loss: 0.6317 - val_accuracy: 0.6381

Epoch 344/900

355/355 [==============================] - 7s 19ms/step - loss: 11.1940 - accuracy: 0.6530 - val_loss: 0.6312 - val_accuracy: 0.6418

Epoch 345/900

355/355 [==============================] - 7s 19ms/step - loss: 11.1615 - accuracy: 0.6518 - val_loss: 0.6339 - val_accuracy: 0.6402

Epoch 346/900

355/355 [==============================] - 7s 19ms/step - loss: 11.2170 - accuracy: 0.6504 - val_loss: 0.6317 - val_accuracy: 0.6418

Epoch 347/900

355/355 [==============================] - 7s 19ms/step - loss: 11.1865 - accuracy: 0.6499 - val_loss: 0.6286 - val_accuracy: 0.6418

Epoch 348/900

355/355 [==============================] - 7s 19ms/step - loss: 11.2296 - accuracy: 0.6488 - val_loss: 0.6267 - val_accuracy: 0.6397

Epoch 349/900

355/355 [==============================] - 7s 19ms/step - loss: 11.1366 - accuracy: 0.6538 - val_loss: 0.6284 - val_accuracy: 0.6445

Epoch 350/900

355/355 [==============================] - 7s 19ms/step - loss: 11.1204 - accuracy: 0.6520 - val_loss: 0.6254 - val_accuracy: 0.6466

Epoch 351/900

355/355 [==============================] - 7s 19ms/step - loss: 11.1068 - accuracy: 0.6544 - val_loss: 0.6342 - val_accuracy: 0.6349

Epoch 352/900

355/355 [==============================] - 7s 19ms/step - loss: 11.1325 - accuracy: 0.6548 - val_loss: 0.6254 - val_accuracy: 0.6359

Epoch 353/900

355/355 [==============================] - 7s 19ms/step - loss: 11.1506 - accuracy: 0.6530 - val_loss: 0.6344 - val_accuracy: 0.6365

Epoch 354/900

355/355 [==============================] - 7s 19ms/step - loss: 11.1048 - accuracy: 0.6524 - val_loss: 0.6276 - val_accuracy: 0.6440

Epoch 355/900

355/355 [==============================] - 7s 19ms/step - loss: 11.1499 - accuracy: 0.6538 - val_loss: 0.6245 - val_accuracy: 0.6429

Epoch 356/900

355/355 [==============================] - 7s 19ms/step - loss: 11.1381 - accuracy: 0.6545 - val_loss: 0.6336 - val_accuracy: 0.6402

Epoch 357/900

355/355 [==============================] - 7s 19ms/step - loss: 11.1147 - accuracy: 0.6568 - val_loss: 0.6314 - val_accuracy: 0.6386

Epoch 358/900

355/355 [==============================] - 7s 19ms/step - loss: 11.1368 - accuracy: 0.6567 - val_loss: 0.6272 - val_accuracy: 0.6450

Epoch 359/900

355/355 [==============================] - 7s 19ms/step - loss: 11.1770 - accuracy: 0.6487 - val_loss: 0.6248 - val_accuracy: 0.6413

Epoch 360/900

355/355 [==============================] - 7s 19ms/step - loss: 11.1491 - accuracy: 0.6515 - val_loss: 0.6319 - val_accuracy: 0.6408

Epoch 361/900

355/355 [==============================] - 7s 19ms/step - loss: 11.0704 - accuracy: 0.6581 - val_loss: 0.6242 - val_accuracy: 0.6450

Epoch 362/900

355/355 [==============================] - 7s 19ms/step - loss: 11.1013 - accuracy: 0.6565 - val_loss: 0.6274 - val_accuracy: 0.6466

Epoch 363/900

355/355 [==============================] - 7s 19ms/step - loss: 11.1144 - accuracy: 0.6540 - val_loss: 0.6310 - val_accuracy: 0.6440

Epoch 364/900

355/355 [==============================] - 7s 19ms/step - loss: 11.1108 - accuracy: 0.6559 - val_loss: 0.6251 - val_accuracy: 0.6413

Epoch 365/900

355/355 [==============================] - 7s 19ms/step - loss: 11.0630 - accuracy: 0.6588 - val_loss: 0.6237 - val_accuracy: 0.6391

Epoch 366/900

355/355 [==============================] - 7s 19ms/step - loss: 11.0934 - accuracy: 0.6569 - val_loss: 0.6215 - val_accuracy: 0.6483

Epoch 367/900

355/355 [==============================] - 7s 19ms/step - loss: 11.1534 - accuracy: 0.6538 - val_loss: 0.6286 - val_accuracy: 0.6391

Epoch 368/900

355/355 [==============================] - 7s 19ms/step - loss: 11.0031 - accuracy: 0.6635 - val_loss: 0.6322 - val_accuracy: 0.6365

Epoch 369/900

355/355 [==============================] - 7s 19ms/step - loss: 11.1000 - accuracy: 0.6552 - val_loss: 0.6377 - val_accuracy: 0.6295

Epoch 370/900

355/355 [==============================] - 7s 19ms/step - loss: 11.0989 - accuracy: 0.6588 - val_loss: 0.6253 - val_accuracy: 0.6450

Epoch 371/900

355/355 [==============================] - 7s 19ms/step - loss: 11.1046 - accuracy: 0.6528 - val_loss: 0.6231 - val_accuracy: 0.6466

Epoch 372/900

355/355 [==============================] - 7s 19ms/step - loss: 11.1416 - accuracy: 0.6549 - val_loss: 0.6232 - val_accuracy: 0.6440

Epoch 373/900

355/355 [==============================] - 7s 19ms/step - loss: 11.1240 - accuracy: 0.6556 - val_loss: 0.6214 - val_accuracy: 0.6466

Epoch 374/900

355/355 [==============================] - 7s 19ms/step - loss: 11.1258 - accuracy: 0.6527 - val_loss: 0.6224 - val_accuracy: 0.6466

Epoch 375/900

355/355 [==============================] - 7s 19ms/step - loss: 11.0482 - accuracy: 0.6601 - val_loss: 0.6216 - val_accuracy: 0.6434

Epoch 376/900

355/355 [==============================] - 7s 19ms/step - loss: 11.1232 - accuracy: 0.6581 - val_loss: 0.6258 - val_accuracy: 0.6418

Epoch 377/900

355/355 [==============================] - 7s 19ms/step - loss: 11.1207 - accuracy: 0.6525 - val_loss: 0.6274 - val_accuracy: 0.6402

Epoch 378/900

355/355 [==============================] - 7s 19ms/step - loss: 11.0148 - accuracy: 0.6584 - val_loss: 0.6248 - val_accuracy: 0.6424

Epoch 379/900

355/355 [==============================] - 7s 19ms/step - loss: 11.1263 - accuracy: 0.6552 - val_loss: 0.6255 - val_accuracy: 0.6445

Epoch 380/900

355/355 [==============================] - 7s 19ms/step - loss: 11.1425 - accuracy: 0.6545 - val_loss: 0.6294 - val_accuracy: 0.6408

Epoch 381/900

355/355 [==============================] - 7s 19ms/step - loss: 11.0047 - accuracy: 0.6605 - val_loss: 0.6259 - val_accuracy: 0.6445

Epoch 382/900

355/355 [==============================] - 7s 19ms/step - loss: 11.1184 - accuracy: 0.6584 - val_loss: 0.6228 - val_accuracy: 0.6456

Epoch 383/900

355/355 [==============================] - 7s 19ms/step - loss: 11.0294 - accuracy: 0.6629 - val_loss: 0.6213 - val_accuracy: 0.6472

Epoch 384/900

355/355 [==============================] - 7s 19ms/step - loss: 11.0682 - accuracy: 0.6603 - val_loss: 0.6371 - val_accuracy: 0.6311

Epoch 385/900

355/355 [==============================] - 7s 19ms/step - loss: 11.0548 - accuracy: 0.6557 - val_loss: 0.6228 - val_accuracy: 0.6440

Epoch 386/900

355/355 [==============================] - 7s 19ms/step - loss: 11.0974 - accuracy: 0.6561 - val_loss: 0.6236 - val_accuracy: 0.6391

Epoch 387/900

355/355 [==============================] - 7s 19ms/step - loss: 11.0755 - accuracy: 0.6569 - val_loss: 0.6189 - val_accuracy: 0.6493

Epoch 388/900

355/355 [==============================] - 7s 19ms/step - loss: 11.0234 - accuracy: 0.6599 - val_loss: 0.6330 - val_accuracy: 0.6397

Epoch 389/900

355/355 [==============================] - 7s 19ms/step - loss: 10.9989 - accuracy: 0.6648 - val_loss: 0.6205 - val_accuracy: 0.6461

Epoch 390/900

355/355 [==============================] - 7s 19ms/step - loss: 10.9513 - accuracy: 0.6618 - val_loss: 0.6239 - val_accuracy: 0.6440

Epoch 391/900

355/355 [==============================] - 7s 19ms/step - loss: 11.0500 - accuracy: 0.6585 - val_loss: 0.6206 - val_accuracy: 0.6466

Epoch 392/900

355/355 [==============================] - 7s 19ms/step - loss: 11.0279 - accuracy: 0.6593 - val_loss: 0.6237 - val_accuracy: 0.6445

Epoch 393/900

355/355 [==============================] - 7s 19ms/step - loss: 11.0299 - accuracy: 0.6592 - val_loss: 0.6189 - val_accuracy: 0.6504

Epoch 394/900

355/355 [==============================] - 7s 19ms/step - loss: 10.9712 - accuracy: 0.6607 - val_loss: 0.6219 - val_accuracy: 0.6450

Epoch 395/900

355/355 [==============================] - 7s 19ms/step - loss: 10.9767 - accuracy: 0.6644 - val_loss: 0.6234 - val_accuracy: 0.6466

Epoch 396/900

355/355 [==============================] - 7s 19ms/step - loss: 10.9231 - accuracy: 0.6646 - val_loss: 0.6234 - val_accuracy: 0.6472

Epoch 397/900

355/355 [==============================] - 7s 19ms/step - loss: 10.9488 - accuracy: 0.6651 - val_loss: 0.6217 - val_accuracy: 0.6493

Epoch 398/900

355/355 [==============================] - 7s 19ms/step - loss: 11.0127 - accuracy: 0.6603 - val_loss: 0.6238 - val_accuracy: 0.6456

Epoch 399/900

355/355 [==============================] - 7s 19ms/step - loss: 11.0826 - accuracy: 0.6566 - val_loss: 0.6229 - val_accuracy: 0.6499

Epoch 400/900

355/355 [==============================] - 7s 19ms/step - loss: 10.9753 - accuracy: 0.6646 - val_loss: 0.6211 - val_accuracy: 0.6477

Epoch 401/900

355/355 [==============================] - 7s 19ms/step - loss: 10.9260 - accuracy: 0.6659 - val_loss: 0.6213 - val_accuracy: 0.6472

Epoch 402/900

355/355 [==============================] - 7s 19ms/step - loss: 10.9656 - accuracy: 0.6632 - val_loss: 0.6291 - val_accuracy: 0.6391

Epoch 403/900

355/355 [==============================] - 7s 19ms/step - loss: 10.9684 - accuracy: 0.6635 - val_loss: 0.6241 - val_accuracy: 0.6413

Epoch 404/900

355/355 [==============================] - 7s 19ms/step - loss: 10.9441 - accuracy: 0.6658 - val_loss: 0.6188 - val_accuracy: 0.6483

Epoch 405/900

355/355 [==============================] - 7s 19ms/step - loss: 10.9993 - accuracy: 0.6647 - val_loss: 0.6296 - val_accuracy: 0.6450

Epoch 406/900

355/355 [==============================] - 7s 19ms/step - loss: 11.0182 - accuracy: 0.6603 - val_loss: 0.6272 - val_accuracy: 0.6418

Epoch 407/900

355/355 [==============================] - 7s 19ms/step - loss: 10.9867 - accuracy: 0.6614 - val_loss: 0.6199 - val_accuracy: 0.6509

Epoch 408/900

355/355 [==============================] - 7s 19ms/step - loss: 10.9031 - accuracy: 0.6672 - val_loss: 0.6161 - val_accuracy: 0.6515

Epoch 409/900

355/355 [==============================] - 7s 19ms/step - loss: 10.9777 - accuracy: 0.6640 - val_loss: 0.6225 - val_accuracy: 0.6483

Epoch 410/900

355/355 [==============================] - 7s 19ms/step - loss: 10.9250 - accuracy: 0.6664 - val_loss: 0.6213 - val_accuracy: 0.6477

Epoch 411/900

355/355 [==============================] - 7s 19ms/step - loss: 10.9850 - accuracy: 0.6661 - val_loss: 0.6289 - val_accuracy: 0.6461

Epoch 412/900

355/355 [==============================] - 7s 19ms/step - loss: 10.8976 - accuracy: 0.6652 - val_loss: 0.6336 - val_accuracy: 0.6413

Epoch 413/900

355/355 [==============================] - 7s 19ms/step - loss: 11.0105 - accuracy: 0.6590 - val_loss: 0.6212 - val_accuracy: 0.6509

Epoch 414/900

355/355 [==============================] - 7s 19ms/step - loss: 10.9148 - accuracy: 0.6655 - val_loss: 0.6302 - val_accuracy: 0.6456

Epoch 415/900

355/355 [==============================] - 7s 19ms/step - loss: 10.8926 - accuracy: 0.6680 - val_loss: 0.6275 - val_accuracy: 0.6488

Epoch 416/900

355/355 [==============================] - 7s 19ms/step - loss: 10.8922 - accuracy: 0.6633 - val_loss: 0.6265 - val_accuracy: 0.6499

Epoch 417/900

355/355 [==============================] - 7s 19ms/step - loss: 10.8892 - accuracy: 0.6668 - val_loss: 0.6234 - val_accuracy: 0.6477

Epoch 418/900

355/355 [==============================] - 7s 19ms/step - loss: 10.9550 - accuracy: 0.6632 - val_loss: 0.6223 - val_accuracy: 0.6509

Epoch 419/900

355/355 [==============================] - 7s 19ms/step - loss: 10.8794 - accuracy: 0.6686 - val_loss: 0.6243 - val_accuracy: 0.6472

Epoch 420/900

355/355 [==============================] - 7s 19ms/step - loss: 10.9159 - accuracy: 0.6629 - val_loss: 0.6173 - val_accuracy: 0.6477

Epoch 421/900

355/355 [==============================] - 7s 19ms/step - loss: 10.9013 - accuracy: 0.6652 - val_loss: 0.6187 - val_accuracy: 0.6477

Epoch 422/900

355/355 [==============================] - 7s 19ms/step - loss: 11.0639 - accuracy: 0.6636 - val_loss: 0.6174 - val_accuracy: 0.6499

Epoch 423/900

355/355 [==============================] - 7s 19ms/step - loss: 10.9423 - accuracy: 0.6633 - val_loss: 0.6257 - val_accuracy: 0.6445

Epoch 424/900

355/355 [==============================] - 7s 19ms/step - loss: 10.9686 - accuracy: 0.6684 - val_loss: 0.6212 - val_accuracy: 0.6504

Epoch 425/900

355/355 [==============================] - 7s 19ms/step - loss: 10.9086 - accuracy: 0.6662 - val_loss: 0.6256 - val_accuracy: 0.6450

Epoch 426/900

355/355 [==============================] - 7s 19ms/step - loss: 10.8913 - accuracy: 0.6675 - val_loss: 0.6203 - val_accuracy: 0.6499

Epoch 427/900

355/355 [==============================] - 7s 19ms/step - loss: 10.8832 - accuracy: 0.6673 - val_loss: 0.6207 - val_accuracy: 0.6483

Epoch 428/900

355/355 [==============================] - 7s 19ms/step - loss: 10.8927 - accuracy: 0.6667 - val_loss: 0.6180 - val_accuracy: 0.6499

Epoch 429/900

355/355 [==============================] - 7s 19ms/step - loss: 10.8363 - accuracy: 0.6716 - val_loss: 0.6215 - val_accuracy: 0.6493

Epoch 430/900

355/355 [==============================] - 7s 19ms/step - loss: 10.8709 - accuracy: 0.6666 - val_loss: 0.6170 - val_accuracy: 0.6466

Epoch 431/900

355/355 [==============================] - 7s 19ms/step - loss: 10.8787 - accuracy: 0.6658 - val_loss: 0.6172 - val_accuracy: 0.6499

Epoch 432/900

355/355 [==============================] - 7s 19ms/step - loss: 10.9152 - accuracy: 0.6698 - val_loss: 0.6194 - val_accuracy: 0.6477

Epoch 433/900

355/355 [==============================] - 7s 19ms/step - loss: 10.9027 - accuracy: 0.6681 - val_loss: 0.6187 - val_accuracy: 0.6531

Epoch 434/900

355/355 [==============================] - 7s 19ms/step - loss: 10.8371 - accuracy: 0.6709 - val_loss: 0.6173 - val_accuracy: 0.6515

Epoch 435/900

355/355 [==============================] - 7s 19ms/step - loss: 10.8112 - accuracy: 0.6729 - val_loss: 0.6222 - val_accuracy: 0.6424

Epoch 436/900

355/355 [==============================] - 7s 19ms/step - loss: 10.8705 - accuracy: 0.6742 - val_loss: 0.6243 - val_accuracy: 0.6472

Epoch 437/900

355/355 [==============================] - 7s 19ms/step - loss: 10.8600 - accuracy: 0.6695 - val_loss: 0.6226 - val_accuracy: 0.6456

Epoch 438/900

355/355 [==============================] - 7s 19ms/step - loss: 10.7940 - accuracy: 0.6712 - val_loss: 0.6161 - val_accuracy: 0.6515

Epoch 439/900

355/355 [==============================] - 7s 19ms/step - loss: 10.8660 - accuracy: 0.6729 - val_loss: 0.6185 - val_accuracy: 0.6525

Epoch 440/900

355/355 [==============================] - 7s 19ms/step - loss: 10.9156 - accuracy: 0.6644 - val_loss: 0.6181 - val_accuracy: 0.6536

Epoch 441/900

355/355 [==============================] - 7s 19ms/step - loss: 10.8298 - accuracy: 0.6708 - val_loss: 0.6138 - val_accuracy: 0.6590

Epoch 442/900

355/355 [==============================] - 7s 19ms/step - loss: 10.8182 - accuracy: 0.6685 - val_loss: 0.6163 - val_accuracy: 0.6547

Epoch 443/900

355/355 [==============================] - 7s 19ms/step - loss: 10.7917 - accuracy: 0.6733 - val_loss: 0.6174 - val_accuracy: 0.6499

Epoch 444/900

355/355 [==============================] - 7s 19ms/step - loss: 10.7989 - accuracy: 0.6733 - val_loss: 0.6198 - val_accuracy: 0.6520

Epoch 445/900

355/355 [==============================] - 7s 19ms/step - loss: 10.8332 - accuracy: 0.6694 - val_loss: 0.6202 - val_accuracy: 0.6488

Epoch 446/900

355/355 [==============================] - 7s 19ms/step - loss: 10.8363 - accuracy: 0.6708 - val_loss: 0.6106 - val_accuracy: 0.6568

Epoch 447/900

355/355 [==============================] - 7s 19ms/step - loss: 10.8301 - accuracy: 0.6714 - val_loss: 0.6194 - val_accuracy: 0.6504

Epoch 448/900

355/355 [==============================] - 7s 19ms/step - loss: 10.7598 - accuracy: 0.6733 - val_loss: 0.6194 - val_accuracy: 0.6472

Epoch 449/900

355/355 [==============================] - 7s 19ms/step - loss: 10.7914 - accuracy: 0.6728 - val_loss: 0.6191 - val_accuracy: 0.6499

Epoch 450/900

355/355 [==============================] - 7s 19ms/step - loss: 10.8234 - accuracy: 0.6706 - val_loss: 0.6174 - val_accuracy: 0.6525

Epoch 451/900

355/355 [==============================] - 7s 19ms/step - loss: 10.7459 - accuracy: 0.6747 - val_loss: 0.6188 - val_accuracy: 0.6483

Epoch 452/900

355/355 [==============================] - 7s 19ms/step - loss: 10.7700 - accuracy: 0.6755 - val_loss: 0.6114 - val_accuracy: 0.6601

Epoch 453/900

355/355 [==============================] - 7s 19ms/step - loss: 10.8086 - accuracy: 0.6743 - val_loss: 0.6153 - val_accuracy: 0.6552

Epoch 454/900

355/355 [==============================] - 7s 19ms/step - loss: 10.7762 - accuracy: 0.6758 - val_loss: 0.6142 - val_accuracy: 0.6547

Epoch 455/900

355/355 [==============================] - 7s 19ms/step - loss: 10.7973 - accuracy: 0.6727 - val_loss: 0.6152 - val_accuracy: 0.6558

Epoch 456/900

355/355 [==============================] - 7s 19ms/step - loss: 10.8440 - accuracy: 0.6729 - val_loss: 0.6161 - val_accuracy: 0.6542

Epoch 457/900

355/355 [==============================] - 7s 19ms/step - loss: 10.8821 - accuracy: 0.6669 - val_loss: 0.6176 - val_accuracy: 0.6525

Epoch 458/900

355/355 [==============================] - 7s 19ms/step - loss: 10.7623 - accuracy: 0.6731 - val_loss: 0.6216 - val_accuracy: 0.6509

Epoch 459/900

355/355 [==============================] - 7s 19ms/step - loss: 10.7212 - accuracy: 0.6770 - val_loss: 0.6136 - val_accuracy: 0.6568

Epoch 460/900

355/355 [==============================] - 7s 19ms/step - loss: 10.7976 - accuracy: 0.6718 - val_loss: 0.6147 - val_accuracy: 0.6531

Epoch 461/900

355/355 [==============================] - 7s 19ms/step - loss: 10.7481 - accuracy: 0.6739 - val_loss: 0.6152 - val_accuracy: 0.6542

Epoch 462/900

355/355 [==============================] - 7s 19ms/step - loss: 10.6862 - accuracy: 0.6779 - val_loss: 0.6152 - val_accuracy: 0.6558

Epoch 463/900

355/355 [==============================] - 7s 19ms/step - loss: 10.6588 - accuracy: 0.6820 - val_loss: 0.6169 - val_accuracy: 0.6483

Epoch 464/900

355/355 [==============================] - 7s 19ms/step - loss: 10.7451 - accuracy: 0.6786 - val_loss: 0.6098 - val_accuracy: 0.6584

Epoch 465/900

355/355 [==============================] - 7s 19ms/step - loss: 10.7270 - accuracy: 0.6764 - val_loss: 0.6182 - val_accuracy: 0.6504

Epoch 466/900

355/355 [==============================] - 7s 19ms/step - loss: 10.7566 - accuracy: 0.6741 - val_loss: 0.6110 - val_accuracy: 0.6654

Epoch 467/900

355/355 [==============================] - 7s 19ms/step - loss: 10.7629 - accuracy: 0.6735 - val_loss: 0.6169 - val_accuracy: 0.6531

Epoch 468/900

355/355 [==============================] - 7s 19ms/step - loss: 10.7828 - accuracy: 0.6727 - val_loss: 0.6179 - val_accuracy: 0.6488

Epoch 469/900

355/355 [==============================] - 7s 19ms/step - loss: 10.8184 - accuracy: 0.6733 - val_loss: 0.6190 - val_accuracy: 0.6483

Epoch 470/900

355/355 [==============================] - 7s 19ms/step - loss: 10.8043 - accuracy: 0.6690 - val_loss: 0.6166 - val_accuracy: 0.6504

Epoch 471/900

355/355 [==============================] - 7s 19ms/step - loss: 10.7244 - accuracy: 0.6733 - val_loss: 0.6131 - val_accuracy: 0.6579

Epoch 472/900

355/355 [==============================] - 7s 19ms/step - loss: 10.7373 - accuracy: 0.6753 - val_loss: 0.6149 - val_accuracy: 0.6542

Epoch 473/900

355/355 [==============================] - 7s 19ms/step - loss: 10.6815 - accuracy: 0.6772 - val_loss: 0.6131 - val_accuracy: 0.6558

Epoch 474/900

355/355 [==============================] - 7s 19ms/step - loss: 10.7142 - accuracy: 0.6739 - val_loss: 0.6173 - val_accuracy: 0.6568

Epoch 475/900

355/355 [==============================] - 7s 19ms/step - loss: 10.7046 - accuracy: 0.6772 - val_loss: 0.6152 - val_accuracy: 0.6584

Epoch 476/900

355/355 [==============================] - 7s 19ms/step - loss: 10.7251 - accuracy: 0.6788 - val_loss: 0.6106 - val_accuracy: 0.6558

Epoch 477/900

355/355 [==============================] - 7s 19ms/step - loss: 10.7950 - accuracy: 0.6717 - val_loss: 0.6176 - val_accuracy: 0.6563

Epoch 478/900

355/355 [==============================] - 7s 19ms/step - loss: 10.7600 - accuracy: 0.6738 - val_loss: 0.6108 - val_accuracy: 0.6542

Epoch 479/900

355/355 [==============================] - 7s 19ms/step - loss: 10.6783 - accuracy: 0.6784 - val_loss: 0.6096 - val_accuracy: 0.6654

Epoch 480/900

355/355 [==============================] - 7s 19ms/step - loss: 10.6771 - accuracy: 0.6780 - val_loss: 0.6115 - val_accuracy: 0.6601

Epoch 481/900

355/355 [==============================] - 7s 19ms/step - loss: 10.7152 - accuracy: 0.6726 - val_loss: 0.6163 - val_accuracy: 0.6536

Epoch 482/900

355/355 [==============================] - 7s 19ms/step - loss: 10.6820 - accuracy: 0.6766 - val_loss: 0.6116 - val_accuracy: 0.6515

Epoch 483/900

355/355 [==============================] - 7s 19ms/step - loss: 10.6769 - accuracy: 0.6807 - val_loss: 0.6094 - val_accuracy: 0.6595

Epoch 484/900

355/355 [==============================] - 7s 19ms/step - loss: 10.6857 - accuracy: 0.6741 - val_loss: 0.6097 - val_accuracy: 0.6601

Epoch 485/900

355/355 [==============================] - 7s 19ms/step - loss: 10.6944 - accuracy: 0.6797 - val_loss: 0.6226 - val_accuracy: 0.6466

Epoch 486/900

355/355 [==============================] - 7s 19ms/step - loss: 10.6428 - accuracy: 0.6797 - val_loss: 0.6077 - val_accuracy: 0.6660

Epoch 487/900

355/355 [==============================] - 7s 19ms/step - loss: 10.6551 - accuracy: 0.6792 - val_loss: 0.6084 - val_accuracy: 0.6627

Epoch 488/900

355/355 [==============================] - 7s 19ms/step - loss: 10.6525 - accuracy: 0.6775 - val_loss: 0.6092 - val_accuracy: 0.6601

Epoch 489/900

355/355 [==============================] - 7s 19ms/step - loss: 10.6337 - accuracy: 0.6795 - val_loss: 0.6127 - val_accuracy: 0.6536

Epoch 490/900

355/355 [==============================] - 7s 19ms/step - loss: 10.6933 - accuracy: 0.6809 - val_loss: 0.6154 - val_accuracy: 0.6542

Epoch 491/900

355/355 [==============================] - 7s 19ms/step - loss: 10.5493 - accuracy: 0.6843 - val_loss: 0.6076 - val_accuracy: 0.6654

Epoch 492/900

355/355 [==============================] - 7s 19ms/step - loss: 10.6181 - accuracy: 0.6827 - val_loss: 0.6048 - val_accuracy: 0.6649

Epoch 493/900

355/355 [==============================] - 7s 19ms/step - loss: 10.6619 - accuracy: 0.6815 - val_loss: 0.6225 - val_accuracy: 0.6542

Epoch 494/900

355/355 [==============================] - 7s 19ms/step - loss: 10.6311 - accuracy: 0.6801 - val_loss: 0.6095 - val_accuracy: 0.6654

Epoch 495/900

355/355 [==============================] - 7s 19ms/step - loss: 10.6437 - accuracy: 0.6811 - val_loss: 0.6090 - val_accuracy: 0.6627

Epoch 496/900

355/355 [==============================] - 7s 19ms/step - loss: 10.6257 - accuracy: 0.6834 - val_loss: 0.6101 - val_accuracy: 0.6601

Epoch 497/900

355/355 [==============================] - 7s 19ms/step - loss: 10.6832 - accuracy: 0.6811 - val_loss: 0.6114 - val_accuracy: 0.6665

Epoch 498/900

355/355 [==============================] - 7s 19ms/step - loss: 10.6184 - accuracy: 0.6786 - val_loss: 0.6067 - val_accuracy: 0.6633

Epoch 499/900

355/355 [==============================] - 7s 19ms/step - loss: 10.6438 - accuracy: 0.6796 - val_loss: 0.6116 - val_accuracy: 0.6633

Epoch 500/900

355/355 [==============================] - 7s 19ms/step - loss: 10.6175 - accuracy: 0.6793 - val_loss: 0.6206 - val_accuracy: 0.6515

Epoch 501/900

355/355 [==============================] - 7s 19ms/step - loss: 10.5581 - accuracy: 0.6804 - val_loss: 0.6192 - val_accuracy: 0.6520

Epoch 502/900

355/355 [==============================] - 7s 19ms/step - loss: 10.6152 - accuracy: 0.6842 - val_loss: 0.6086 - val_accuracy: 0.6622

Epoch 503/900

355/355 [==============================] - 7s 20ms/step - loss: 10.7194 - accuracy: 0.6766 - val_loss: 0.6116 - val_accuracy: 0.6558

Epoch 504/900

355/355 [==============================] - 7s 19ms/step - loss: 10.5504 - accuracy: 0.6873 - val_loss: 0.6124 - val_accuracy: 0.6579

Epoch 505/900

355/355 [==============================] - 7s 19ms/step - loss: 10.6122 - accuracy: 0.6842 - val_loss: 0.6182 - val_accuracy: 0.6584

Epoch 506/900

355/355 [==============================] - 7s 19ms/step - loss: 10.6019 - accuracy: 0.6802 - val_loss: 0.6095 - val_accuracy: 0.6584

Epoch 507/900

355/355 [==============================] - 7s 19ms/step - loss: 10.5966 - accuracy: 0.6802 - val_loss: 0.6081 - val_accuracy: 0.6633

Epoch 508/900

355/355 [==============================] - 7s 19ms/step - loss: 10.6191 - accuracy: 0.6837 - val_loss: 0.6145 - val_accuracy: 0.6595

Epoch 509/900

355/355 [==============================] - 7s 19ms/step - loss: 10.6781 - accuracy: 0.6785 - val_loss: 0.6078 - val_accuracy: 0.6627

Epoch 510/900

355/355 [==============================] - 7s 19ms/step - loss: 10.5213 - accuracy: 0.6841 - val_loss: 0.6123 - val_accuracy: 0.6590

Epoch 511/900

355/355 [==============================] - 7s 19ms/step - loss: 10.6156 - accuracy: 0.6864 - val_loss: 0.6074 - val_accuracy: 0.6681

Epoch 512/900

355/355 [==============================] - 7s 19ms/step - loss: 10.6192 - accuracy: 0.6833 - val_loss: 0.6112 - val_accuracy: 0.6627

Epoch 513/900

355/355 [==============================] - 7s 19ms/step - loss: 10.5953 - accuracy: 0.6818 - val_loss: 0.6090 - val_accuracy: 0.6558

Epoch 514/900

355/355 [==============================] - 7s 19ms/step - loss: 10.6563 - accuracy: 0.6773 - val_loss: 0.6149 - val_accuracy: 0.6590

Epoch 515/900

355/355 [==============================] - 7s 19ms/step - loss: 10.6679 - accuracy: 0.6749 - val_loss: 0.6065 - val_accuracy: 0.6633

Epoch 516/900

355/355 [==============================] - 7s 19ms/step - loss: 10.5926 - accuracy: 0.6791 - val_loss: 0.6106 - val_accuracy: 0.6611

Epoch 517/900

355/355 [==============================] - 7s 19ms/step - loss: 10.6079 - accuracy: 0.6817 - val_loss: 0.6046 - val_accuracy: 0.6660

Epoch 518/900

355/355 [==============================] - 7s 19ms/step - loss: 10.5385 - accuracy: 0.6832 - val_loss: 0.6019 - val_accuracy: 0.6686

Epoch 519/900

355/355 [==============================] - 7s 19ms/step - loss: 10.5440 - accuracy: 0.6824 - val_loss: 0.6048 - val_accuracy: 0.6601

Epoch 520/900

355/355 [==============================] - 7s 19ms/step - loss: 10.5918 - accuracy: 0.6811 - val_loss: 0.6091 - val_accuracy: 0.6579

Epoch 521/900

355/355 [==============================] - 7s 19ms/step - loss: 10.5853 - accuracy: 0.6802 - val_loss: 0.6140 - val_accuracy: 0.6558

Epoch 522/900

355/355 [==============================] - 7s 19ms/step - loss: 10.4995 - accuracy: 0.6848 - val_loss: 0.6066 - val_accuracy: 0.6633

Epoch 523/900

355/355 [==============================] - 7s 19ms/step - loss: 10.6198 - accuracy: 0.6842 - val_loss: 0.6103 - val_accuracy: 0.6601

Epoch 524/900

355/355 [==============================] - 7s 19ms/step - loss: 10.6399 - accuracy: 0.6818 - val_loss: 0.6014 - val_accuracy: 0.6665

Epoch 525/900

355/355 [==============================] - 7s 19ms/step - loss: 10.5713 - accuracy: 0.6860 - val_loss: 0.6055 - val_accuracy: 0.6627

Epoch 526/900

355/355 [==============================] - 7s 19ms/step - loss: 10.5362 - accuracy: 0.6857 - val_loss: 0.6065 - val_accuracy: 0.6606

Epoch 527/900

355/355 [==============================] - 7s 19ms/step - loss: 10.6281 - accuracy: 0.6821 - val_loss: 0.6061 - val_accuracy: 0.6622

Epoch 528/900

355/355 [==============================] - 7s 19ms/step - loss: 10.5515 - accuracy: 0.6847 - val_loss: 0.6138 - val_accuracy: 0.6622

Epoch 529/900

355/355 [==============================] - 7s 19ms/step - loss: 10.5240 - accuracy: 0.6881 - val_loss: 0.6042 - val_accuracy: 0.6595

Epoch 530/900

355/355 [==============================] - 7s 19ms/step - loss: 10.4557 - accuracy: 0.6890 - val_loss: 0.6042 - val_accuracy: 0.6617

Epoch 531/900

355/355 [==============================] - 7s 19ms/step - loss: 10.5839 - accuracy: 0.6875 - val_loss: 0.6126 - val_accuracy: 0.6617

Epoch 532/900

355/355 [==============================] - 7s 19ms/step - loss: 10.5882 - accuracy: 0.6824 - val_loss: 0.6044 - val_accuracy: 0.6649

Epoch 533/900

355/355 [==============================] - 7s 19ms/step - loss: 10.5315 - accuracy: 0.6875 - val_loss: 0.6028 - val_accuracy: 0.6665

Epoch 534/900

355/355 [==============================] - 7s 19ms/step - loss: 10.6063 - accuracy: 0.6819 - val_loss: 0.6108 - val_accuracy: 0.6627

Epoch 535/900

355/355 [==============================] - 7s 19ms/step - loss: 10.5325 - accuracy: 0.6850 - val_loss: 0.6140 - val_accuracy: 0.6584

Epoch 536/900

355/355 [==============================] - 7s 19ms/step - loss: 10.5940 - accuracy: 0.6867 - val_loss: 0.6124 - val_accuracy: 0.6617

Epoch 537/900

355/355 [==============================] - 7s 19ms/step - loss: 10.4567 - accuracy: 0.6917 - val_loss: 0.6115 - val_accuracy: 0.6579

Epoch 538/900

355/355 [==============================] - 7s 19ms/step - loss: 10.5650 - accuracy: 0.6867 - val_loss: 0.6055 - val_accuracy: 0.6611

Epoch 539/900

355/355 [==============================] - 7s 19ms/step - loss: 10.5708 - accuracy: 0.6869 - val_loss: 0.6106 - val_accuracy: 0.6611

Epoch 540/900

355/355 [==============================] - 7s 19ms/step - loss: 10.5433 - accuracy: 0.6849 - val_loss: 0.6085 - val_accuracy: 0.6627

Epoch 541/900

355/355 [==============================] - 7s 19ms/step - loss: 10.5053 - accuracy: 0.6889 - val_loss: 0.6226 - val_accuracy: 0.6568

Epoch 542/900

355/355 [==============================] - 7s 19ms/step - loss: 10.5533 - accuracy: 0.6864 - val_loss: 0.6081 - val_accuracy: 0.6606

Epoch 543/900

355/355 [==============================] - 7s 19ms/step - loss: 10.4700 - accuracy: 0.6868 - val_loss: 0.6065 - val_accuracy: 0.6552

Epoch 544/900

355/355 [==============================] - 7s 19ms/step - loss: 10.5247 - accuracy: 0.6893 - val_loss: 0.6029 - val_accuracy: 0.6622

Epoch 545/900

355/355 [==============================] - 7s 19ms/step - loss: 10.5132 - accuracy: 0.6869 - val_loss: 0.6070 - val_accuracy: 0.6617

Epoch 546/900

355/355 [==============================] - 7s 19ms/step - loss: 10.5184 - accuracy: 0.6841 - val_loss: 0.6038 - val_accuracy: 0.6676

Epoch 547/900

355/355 [==============================] - 7s 19ms/step - loss: 10.4756 - accuracy: 0.6921 - val_loss: 0.6110 - val_accuracy: 0.6584

Epoch 548/900

355/355 [==============================] - 7s 19ms/step - loss: 10.3947 - accuracy: 0.6896 - val_loss: 0.6077 - val_accuracy: 0.6606

Epoch 549/900

355/355 [==============================] - 7s 19ms/step - loss: 10.4295 - accuracy: 0.6916 - val_loss: 0.6097 - val_accuracy: 0.6606

Epoch 550/900

355/355 [==============================] - 7s 19ms/step - loss: 10.4582 - accuracy: 0.6853 - val_loss: 0.6083 - val_accuracy: 0.6665

Epoch 551/900

355/355 [==============================] - 7s 19ms/step - loss: 10.3893 - accuracy: 0.6935 - val_loss: 0.6071 - val_accuracy: 0.6611

Epoch 552/900

355/355 [==============================] - 7s 19ms/step - loss: 10.4114 - accuracy: 0.6905 - val_loss: 0.6068 - val_accuracy: 0.6568

Epoch 553/900

355/355 [==============================] - 7s 19ms/step - loss: 10.4227 - accuracy: 0.6903 - val_loss: 0.6113 - val_accuracy: 0.6643

Epoch 554/900

355/355 [==============================] - 7s 19ms/step - loss: 10.4771 - accuracy: 0.6885 - val_loss: 0.6116 - val_accuracy: 0.6617

Epoch 555/900

355/355 [==============================] - 7s 19ms/step - loss: 10.5022 - accuracy: 0.6840 - val_loss: 0.6119 - val_accuracy: 0.6676

Epoch 556/900

355/355 [==============================] - 7s 19ms/step - loss: 10.4671 - accuracy: 0.6884 - val_loss: 0.6108 - val_accuracy: 0.6633

Epoch 557/900

355/355 [==============================] - 7s 19ms/step - loss: 10.3582 - accuracy: 0.6903 - val_loss: 0.6079 - val_accuracy: 0.6622

Epoch 558/900

355/355 [==============================] - 7s 19ms/step - loss: 10.4622 - accuracy: 0.6885 - val_loss: 0.6103 - val_accuracy: 0.6627

Epoch 559/900

355/355 [==============================] - 7s 19ms/step - loss: 10.4493 - accuracy: 0.6928 - val_loss: 0.6066 - val_accuracy: 0.6611

Epoch 560/900

355/355 [==============================] - 7s 19ms/step - loss: 10.4003 - accuracy: 0.6925 - val_loss: 0.6130 - val_accuracy: 0.6595

Epoch 561/900

355/355 [==============================] - 7s 19ms/step - loss: 10.3935 - accuracy: 0.6912 - val_loss: 0.6080 - val_accuracy: 0.6590

Epoch 562/900

355/355 [==============================] - 7s 19ms/step - loss: 10.3929 - accuracy: 0.6947 - val_loss: 0.6076 - val_accuracy: 0.6611

Epoch 563/900

355/355 [==============================] - 7s 19ms/step - loss: 10.4492 - accuracy: 0.6911 - val_loss: 0.6046 - val_accuracy: 0.6617

Epoch 564/900

355/355 [==============================] - 7s 19ms/step - loss: 10.4410 - accuracy: 0.6940 - val_loss: 0.6030 - val_accuracy: 0.6622

Epoch 565/900

355/355 [==============================] - 7s 19ms/step - loss: 10.4316 - accuracy: 0.6889 - val_loss: 0.6217 - val_accuracy: 0.6563

Epoch 566/900

355/355 [==============================] - 7s 19ms/step - loss: 10.4482 - accuracy: 0.6950 - val_loss: 0.6080 - val_accuracy: 0.6622

Epoch 567/900

355/355 [==============================] - 7s 19ms/step - loss: 10.3163 - accuracy: 0.6952 - val_loss: 0.6051 - val_accuracy: 0.6627

Epoch 568/900

355/355 [==============================] - 7s 19ms/step - loss: 10.3317 - accuracy: 0.6959 - val_loss: 0.6071 - val_accuracy: 0.6590

Epoch 569/900

355/355 [==============================] - 7s 19ms/step - loss: 10.4280 - accuracy: 0.6889 - val_loss: 0.6032 - val_accuracy: 0.6654

Epoch 570/900

355/355 [==============================] - 7s 19ms/step - loss: 10.4087 - accuracy: 0.6963 - val_loss: 0.6051 - val_accuracy: 0.6617

Epoch 571/900

355/355 [==============================] - 7s 19ms/step - loss: 10.4760 - accuracy: 0.6914 - val_loss: 0.6040 - val_accuracy: 0.6649

Epoch 572/900

355/355 [==============================] - 7s 19ms/step - loss: 10.5282 - accuracy: 0.6883 - val_loss: 0.6068 - val_accuracy: 0.6611

Epoch 573/900

355/355 [==============================] - 7s 19ms/step - loss: 10.4015 - accuracy: 0.6907 - val_loss: 0.6028 - val_accuracy: 0.6649

Epoch 574/900

355/355 [==============================] - 7s 19ms/step - loss: 10.3702 - accuracy: 0.6937 - val_loss: 0.6057 - val_accuracy: 0.6681

Epoch 575/900

355/355 [==============================] - 7s 19ms/step - loss: 10.3446 - accuracy: 0.6988 - val_loss: 0.6016 - val_accuracy: 0.6638

Epoch 576/900

355/355 [==============================] - 7s 19ms/step - loss: 10.3166 - accuracy: 0.6944 - val_loss: 0.5993 - val_accuracy: 0.6649

Epoch 577/900

355/355 [==============================] - 7s 19ms/step - loss: 10.3459 - accuracy: 0.6973 - val_loss: 0.6071 - val_accuracy: 0.6638

Epoch 578/900

355/355 [==============================] - 7s 19ms/step - loss: 10.3796 - accuracy: 0.6931 - val_loss: 0.6079 - val_accuracy: 0.6649

Epoch 579/900

355/355 [==============================] - 7s 19ms/step - loss: 10.3401 - accuracy: 0.6973 - val_loss: 0.6105 - val_accuracy: 0.6595

Epoch 580/900

355/355 [==============================] - 7s 19ms/step - loss: 10.3566 - accuracy: 0.6915 - val_loss: 0.6065 - val_accuracy: 0.6611

Epoch 581/900

355/355 [==============================] - 7s 19ms/step - loss: 10.3388 - accuracy: 0.6949 - val_loss: 0.6049 - val_accuracy: 0.6638

Epoch 582/900

355/355 [==============================] - 7s 19ms/step - loss: 10.3417 - accuracy: 0.6930 - val_loss: 0.6075 - val_accuracy: 0.6617

Epoch 583/900

355/355 [==============================] - 7s 19ms/step - loss: 10.3678 - accuracy: 0.6912 - val_loss: 0.6031 - val_accuracy: 0.6649

Epoch 584/900

355/355 [==============================] - 7s 19ms/step - loss: 10.3900 - accuracy: 0.6913 - val_loss: 0.6155 - val_accuracy: 0.6547

Epoch 585/900

355/355 [==============================] - 7s 19ms/step - loss: 10.2847 - accuracy: 0.6954 - val_loss: 0.6198 - val_accuracy: 0.6531

Epoch 586/900

355/355 [==============================] - 7s 19ms/step - loss: 10.4536 - accuracy: 0.6925 - val_loss: 0.6094 - val_accuracy: 0.6563

Epoch 587/900

355/355 [==============================] - 7s 19ms/step - loss: 10.2973 - accuracy: 0.6940 - val_loss: 0.6073 - val_accuracy: 0.6595

Epoch 588/900

355/355 [==============================] - 7s 19ms/step - loss: 10.3556 - accuracy: 0.6949 - val_loss: 0.5999 - val_accuracy: 0.6665

Epoch 589/900

355/355 [==============================] - 7s 19ms/step - loss: 10.3663 - accuracy: 0.6971 - val_loss: 0.6043 - val_accuracy: 0.6649

Epoch 590/900

355/355 [==============================] - 7s 19ms/step - loss: 10.3864 - accuracy: 0.6978 - val_loss: 0.6046 - val_accuracy: 0.6601

Epoch 591/900

355/355 [==============================] - 7s 19ms/step - loss: 10.3492 - accuracy: 0.6965 - val_loss: 0.6036 - val_accuracy: 0.6627

Epoch 592/900

355/355 [==============================] - 7s 19ms/step - loss: 10.2622 - accuracy: 0.6954 - val_loss: 0.6134 - val_accuracy: 0.6606

Epoch 593/900

355/355 [==============================] - 7s 19ms/step - loss: 10.3169 - accuracy: 0.6953 - val_loss: 0.6022 - val_accuracy: 0.6697

Epoch 594/900

355/355 [==============================] - 7s 19ms/step - loss: 10.3745 - accuracy: 0.6973 - val_loss: 0.6016 - val_accuracy: 0.6660

Epoch 595/900

355/355 [==============================] - 7s 19ms/step - loss: 10.3543 - accuracy: 0.6946 - val_loss: 0.6162 - val_accuracy: 0.6649

Epoch 596/900

355/355 [==============================] - 7s 19ms/step - loss: 10.3308 - accuracy: 0.6957 - val_loss: 0.5986 - val_accuracy: 0.6654

Epoch 597/900

355/355 [==============================] - 7s 19ms/step - loss: 10.3998 - accuracy: 0.6959 - val_loss: 0.6048 - val_accuracy: 0.6676

Epoch 598/900

355/355 [==============================] - 7s 19ms/step - loss: 10.2825 - accuracy: 0.6977 - val_loss: 0.6065 - val_accuracy: 0.6611

Epoch 599/900

355/355 [==============================] - 7s 19ms/step - loss: 10.3275 - accuracy: 0.6938 - val_loss: 0.6071 - val_accuracy: 0.6627

Epoch 600/900

355/355 [==============================] - 7s 19ms/step - loss: 10.3253 - accuracy: 0.6937 - val_loss: 0.5985 - val_accuracy: 0.6638

Epoch 601/900

355/355 [==============================] - 7s 19ms/step - loss: 10.2597 - accuracy: 0.7002 - val_loss: 0.6050 - val_accuracy: 0.6638

Epoch 602/900

355/355 [==============================] - 7s 19ms/step - loss: 10.2421 - accuracy: 0.7003 - val_loss: 0.5999 - val_accuracy: 0.6708

Epoch 603/900

355/355 [==============================] - 7s 19ms/step - loss: 10.2843 - accuracy: 0.6939 - val_loss: 0.6122 - val_accuracy: 0.6627

Epoch 604/900

355/355 [==============================] - 7s 19ms/step - loss: 10.2502 - accuracy: 0.7011 - val_loss: 0.6015 - val_accuracy: 0.6697

Epoch 605/900

355/355 [==============================] - 7s 19ms/step - loss: 10.2607 - accuracy: 0.6953 - val_loss: 0.6087 - val_accuracy: 0.6617

Epoch 606/900

355/355 [==============================] - 7s 19ms/step - loss: 10.2276 - accuracy: 0.7032 - val_loss: 0.6100 - val_accuracy: 0.6660

Epoch 607/900

355/355 [==============================] - 7s 19ms/step - loss: 10.2229 - accuracy: 0.7028 - val_loss: 0.6050 - val_accuracy: 0.6643

Epoch 608/900

355/355 [==============================] - 7s 19ms/step - loss: 10.2899 - accuracy: 0.6995 - val_loss: 0.6027 - val_accuracy: 0.6686

Epoch 609/900

355/355 [==============================] - 7s 19ms/step - loss: 10.3405 - accuracy: 0.6966 - val_loss: 0.5982 - val_accuracy: 0.6660

Epoch 610/900

355/355 [==============================] - 7s 19ms/step - loss: 10.2726 - accuracy: 0.6995 - val_loss: 0.6049 - val_accuracy: 0.6584

Epoch 611/900

355/355 [==============================] - 7s 19ms/step - loss: 10.1531 - accuracy: 0.7068 - val_loss: 0.6028 - val_accuracy: 0.6638

Epoch 612/900

355/355 [==============================] - 7s 19ms/step - loss: 10.2623 - accuracy: 0.6978 - val_loss: 0.6108 - val_accuracy: 0.6617

Epoch 613/900

355/355 [==============================] - 7s 19ms/step - loss: 10.2710 - accuracy: 0.6969 - val_loss: 0.6051 - val_accuracy: 0.6660

Epoch 614/900

355/355 [==============================] - 7s 19ms/step - loss: 10.2090 - accuracy: 0.6994 - val_loss: 0.6020 - val_accuracy: 0.6649

Epoch 615/900

355/355 [==============================] - 7s 19ms/step - loss: 10.2205 - accuracy: 0.7022 - val_loss: 0.6062 - val_accuracy: 0.6643

Epoch 616/900

355/355 [==============================] - 7s 19ms/step - loss: 10.1802 - accuracy: 0.7029 - val_loss: 0.6066 - val_accuracy: 0.6665

Epoch 617/900

355/355 [==============================] - 7s 19ms/step - loss: 10.2991 - accuracy: 0.6946 - val_loss: 0.6050 - val_accuracy: 0.6649

Epoch 618/900

355/355 [==============================] - 7s 19ms/step - loss: 10.2129 - accuracy: 0.7049 - val_loss: 0.6110 - val_accuracy: 0.6660

Epoch 619/900

355/355 [==============================] - 7s 19ms/step - loss: 10.2205 - accuracy: 0.7051 - val_loss: 0.5992 - val_accuracy: 0.6627

Epoch 620/900

355/355 [==============================] - 7s 19ms/step - loss: 10.2888 - accuracy: 0.7005 - val_loss: 0.5976 - val_accuracy: 0.6670

Epoch 621/900

355/355 [==============================] - 7s 19ms/step - loss: 10.2756 - accuracy: 0.7003 - val_loss: 0.6013 - val_accuracy: 0.6681

Epoch 622/900

355/355 [==============================] - 7s 19ms/step - loss: 10.2453 - accuracy: 0.7027 - val_loss: 0.6096 - val_accuracy: 0.6665

Epoch 623/900

355/355 [==============================] - 7s 19ms/step - loss: 10.2415 - accuracy: 0.6961 - val_loss: 0.6035 - val_accuracy: 0.6649

Epoch 624/900

355/355 [==============================] - 7s 19ms/step - loss: 10.1969 - accuracy: 0.7008 - val_loss: 0.6005 - val_accuracy: 0.6681

Epoch 625/900

355/355 [==============================] - 7s 19ms/step - loss: 10.2333 - accuracy: 0.6982 - val_loss: 0.6023 - val_accuracy: 0.6692

Epoch 626/900

355/355 [==============================] - 7s 19ms/step - loss: 10.2766 - accuracy: 0.6976 - val_loss: 0.6030 - val_accuracy: 0.6686

Epoch 627/900

355/355 [==============================] - 7s 19ms/step - loss: 10.2719 - accuracy: 0.6954 - val_loss: 0.6026 - val_accuracy: 0.6697

Epoch 628/900

355/355 [==============================] - 7s 19ms/step - loss: 10.2443 - accuracy: 0.7026 - val_loss: 0.6013 - val_accuracy: 0.6633

Epoch 629/900

355/355 [==============================] - 7s 19ms/step - loss: 10.1973 - accuracy: 0.7006 - val_loss: 0.5973 - val_accuracy: 0.6670

Epoch 630/900

355/355 [==============================] - 7s 19ms/step - loss: 10.1788 - accuracy: 0.7039 - val_loss: 0.6007 - val_accuracy: 0.6676

Epoch 631/900

355/355 [==============================] - 7s 19ms/step - loss: 10.1702 - accuracy: 0.7069 - val_loss: 0.5993 - val_accuracy: 0.6681

Epoch 632/900

355/355 [==============================] - 7s 19ms/step - loss: 10.1644 - accuracy: 0.7043 - val_loss: 0.6047 - val_accuracy: 0.6649

Epoch 633/900

355/355 [==============================] - 7s 19ms/step - loss: 10.1386 - accuracy: 0.7078 - val_loss: 0.6038 - val_accuracy: 0.6681

Epoch 634/900

355/355 [==============================] - 7s 19ms/step - loss: 10.1084 - accuracy: 0.7038 - val_loss: 0.6033 - val_accuracy: 0.6697

Epoch 635/900

355/355 [==============================] - 7s 19ms/step - loss: 10.1553 - accuracy: 0.7032 - val_loss: 0.6016 - val_accuracy: 0.6670

Epoch 636/900

355/355 [==============================] - 7s 19ms/step - loss: 10.1369 - accuracy: 0.7052 - val_loss: 0.6141 - val_accuracy: 0.6606

Epoch 637/900

355/355 [==============================] - 7s 19ms/step - loss: 10.2312 - accuracy: 0.7018 - val_loss: 0.5964 - val_accuracy: 0.6665

Epoch 638/900

355/355 [==============================] - 7s 19ms/step - loss: 10.0822 - accuracy: 0.7104 - val_loss: 0.5985 - val_accuracy: 0.6670

Epoch 639/900

355/355 [==============================] - 7s 19ms/step - loss: 10.1628 - accuracy: 0.7028 - val_loss: 0.6015 - val_accuracy: 0.6670

Epoch 640/900

355/355 [==============================] - 7s 19ms/step - loss: 10.1540 - accuracy: 0.7077 - val_loss: 0.5997 - val_accuracy: 0.6649

Epoch 641/900

355/355 [==============================] - 7s 19ms/step - loss: 10.1319 - accuracy: 0.7080 - val_loss: 0.6069 - val_accuracy: 0.6665

Epoch 642/900

355/355 [==============================] - 7s 19ms/step - loss: 10.0942 - accuracy: 0.7049 - val_loss: 0.6052 - val_accuracy: 0.6649

Epoch 643/900

355/355 [==============================] - 7s 19ms/step - loss: 10.1882 - accuracy: 0.7029 - val_loss: 0.5997 - val_accuracy: 0.6676

Epoch 644/900

355/355 [==============================] - 7s 19ms/step - loss: 10.1820 - accuracy: 0.7053 - val_loss: 0.6046 - val_accuracy: 0.6617

Epoch 645/900

355/355 [==============================] - 7s 19ms/step - loss: 10.1292 - accuracy: 0.7050 - val_loss: 0.6084 - val_accuracy: 0.6595

Epoch 646/900

355/355 [==============================] - 7s 19ms/step - loss: 10.1707 - accuracy: 0.7019 - val_loss: 0.6024 - val_accuracy: 0.6606

Epoch 647/900

355/355 [==============================] - 7s 19ms/step - loss: 10.1028 - accuracy: 0.7066 - val_loss: 0.5979 - val_accuracy: 0.6611

Epoch 648/900

355/355 [==============================] - 7s 19ms/step - loss: 10.1496 - accuracy: 0.7035 - val_loss: 0.5992 - val_accuracy: 0.6633

Epoch 649/900

355/355 [==============================] - 7s 20ms/step - loss: 9.9955 - accuracy: 0.7091 - val_loss: 0.6029 - val_accuracy: 0.6718

Epoch 650/900

355/355 [==============================] - 7s 19ms/step - loss: 10.1176 - accuracy: 0.7066 - val_loss: 0.5999 - val_accuracy: 0.6643

Epoch 651/900

355/355 [==============================] - 7s 19ms/step - loss: 10.1744 - accuracy: 0.7056 - val_loss: 0.6063 - val_accuracy: 0.6670

Epoch 652/900

355/355 [==============================] - 7s 19ms/step - loss: 10.1288 - accuracy: 0.7032 - val_loss: 0.6009 - val_accuracy: 0.6681

Epoch 653/900

355/355 [==============================] - 7s 19ms/step - loss: 10.1066 - accuracy: 0.7089 - val_loss: 0.6070 - val_accuracy: 0.6697

Epoch 654/900

355/355 [==============================] - 7s 19ms/step - loss: 10.1172 - accuracy: 0.7056 - val_loss: 0.6085 - val_accuracy: 0.6627

Epoch 655/900

355/355 [==============================] - 7s 19ms/step - loss: 10.1636 - accuracy: 0.7063 - val_loss: 0.6004 - val_accuracy: 0.6676

Epoch 656/900

355/355 [==============================] - 7s 19ms/step - loss: 10.1304 - accuracy: 0.7064 - val_loss: 0.6073 - val_accuracy: 0.6670

Epoch 657/900

355/355 [==============================] - 7s 19ms/step - loss: 10.0928 - accuracy: 0.7066 - val_loss: 0.6020 - val_accuracy: 0.6649

Epoch 658/900

355/355 [==============================] - 7s 19ms/step - loss: 10.1953 - accuracy: 0.6991 - val_loss: 0.6027 - val_accuracy: 0.6686

Epoch 659/900

355/355 [==============================] - 7s 19ms/step - loss: 10.0192 - accuracy: 0.7103 - val_loss: 0.6142 - val_accuracy: 0.6611

Epoch 660/900

355/355 [==============================] - 7s 19ms/step - loss: 10.1571 - accuracy: 0.7052 - val_loss: 0.6098 - val_accuracy: 0.6665

Epoch 661/900

355/355 [==============================] - 7s 19ms/step - loss: 10.0325 - accuracy: 0.7065 - val_loss: 0.6011 - val_accuracy: 0.6708

Epoch 662/900

355/355 [==============================] - 7s 19ms/step - loss: 10.0875 - accuracy: 0.7028 - val_loss: 0.5956 - val_accuracy: 0.6670

Epoch 663/900

355/355 [==============================] - 7s 19ms/step - loss: 10.1759 - accuracy: 0.7004 - val_loss: 0.6024 - val_accuracy: 0.6708

Epoch 664/900

355/355 [==============================] - 7s 19ms/step - loss: 10.1107 - accuracy: 0.7043 - val_loss: 0.6039 - val_accuracy: 0.6718

Epoch 665/900

355/355 [==============================] - 7s 19ms/step - loss: 10.0852 - accuracy: 0.7073 - val_loss: 0.6134 - val_accuracy: 0.6654

Epoch 666/900

355/355 [==============================] - 7s 19ms/step - loss: 10.2305 - accuracy: 0.6989 - val_loss: 0.6124 - val_accuracy: 0.6627

Epoch 667/900

355/355 [==============================] - 7s 19ms/step - loss: 10.1284 - accuracy: 0.7009 - val_loss: 0.5993 - val_accuracy: 0.6665

Epoch 668/900

355/355 [==============================] - 7s 19ms/step - loss: 10.0507 - accuracy: 0.7128 - val_loss: 0.6044 - val_accuracy: 0.6665

Epoch 669/900

355/355 [==============================] - 7s 19ms/step - loss: 10.0419 - accuracy: 0.7076 - val_loss: 0.6053 - val_accuracy: 0.6676

Epoch 670/900

355/355 [==============================] - 7s 19ms/step - loss: 10.0296 - accuracy: 0.7102 - val_loss: 0.6080 - val_accuracy: 0.6735

Epoch 671/900

355/355 [==============================] - 7s 19ms/step - loss: 10.0812 - accuracy: 0.7076 - val_loss: 0.6068 - val_accuracy: 0.6643

Epoch 672/900

355/355 [==============================] - 7s 19ms/step - loss: 10.0085 - accuracy: 0.7144 - val_loss: 0.6026 - val_accuracy: 0.6692

Epoch 673/900

355/355 [==============================] - 7s 19ms/step - loss: 10.0858 - accuracy: 0.7076 - val_loss: 0.6075 - val_accuracy: 0.6692

Epoch 674/900

355/355 [==============================] - 7s 19ms/step - loss: 10.0823 - accuracy: 0.7030 - val_loss: 0.6052 - val_accuracy: 0.6713

Epoch 675/900

355/355 [==============================] - 7s 19ms/step - loss: 10.0983 - accuracy: 0.7026 - val_loss: 0.6024 - val_accuracy: 0.6670

Epoch 676/900

355/355 [==============================] - 7s 19ms/step - loss: 10.0814 - accuracy: 0.7108 - val_loss: 0.6051 - val_accuracy: 0.6670

Epoch 677/900

355/355 [==============================] - 7s 19ms/step - loss: 9.9806 - accuracy: 0.7094 - val_loss: 0.6033 - val_accuracy: 0.6713

Epoch 678/900

355/355 [==============================] - 7s 19ms/step - loss: 10.0660 - accuracy: 0.7107 - val_loss: 0.6030 - val_accuracy: 0.6692

Epoch 679/900

355/355 [==============================] - 7s 19ms/step - loss: 10.0689 - accuracy: 0.7111 - val_loss: 0.6009 - val_accuracy: 0.6676

Epoch 680/900

355/355 [==============================] - 7s 19ms/step - loss: 10.1516 - accuracy: 0.7072 - val_loss: 0.6015 - val_accuracy: 0.6692

Epoch 681/900

355/355 [==============================] - 7s 19ms/step - loss: 9.9284 - accuracy: 0.7137 - val_loss: 0.5982 - val_accuracy: 0.6654

Epoch 682/900

355/355 [==============================] - 7s 19ms/step - loss: 9.9779 - accuracy: 0.7125 - val_loss: 0.6089 - val_accuracy: 0.6638

Epoch 683/900

355/355 [==============================] - 7s 19ms/step - loss: 9.9312 - accuracy: 0.7132 - val_loss: 0.6012 - val_accuracy: 0.6702

Epoch 684/900

355/355 [==============================] - 7s 19ms/step - loss: 10.0342 - accuracy: 0.7064 - val_loss: 0.6029 - val_accuracy: 0.6692

Epoch 685/900

355/355 [==============================] - 7s 19ms/step - loss: 10.0158 - accuracy: 0.7103 - val_loss: 0.6012 - val_accuracy: 0.6702

Epoch 686/900

355/355 [==============================] - 7s 19ms/step - loss: 9.8862 - accuracy: 0.7142 - val_loss: 0.6034 - val_accuracy: 0.6724

Epoch 687/900

355/355 [==============================] - 7s 19ms/step - loss: 9.9960 - accuracy: 0.7112 - val_loss: 0.6093 - val_accuracy: 0.6708

Epoch 688/900

355/355 [==============================] - 7s 19ms/step - loss: 10.0238 - accuracy: 0.7125 - val_loss: 0.5997 - val_accuracy: 0.6702

Epoch 689/900

355/355 [==============================] - 7s 19ms/step - loss: 10.0637 - accuracy: 0.7073 - val_loss: 0.6001 - val_accuracy: 0.6686

Epoch 690/900

355/355 [==============================] - 7s 19ms/step - loss: 9.9473 - accuracy: 0.7138 - val_loss: 0.6063 - val_accuracy: 0.6643

Epoch 691/900

355/355 [==============================] - 7s 19ms/step - loss: 10.0623 - accuracy: 0.7087 - val_loss: 0.6038 - val_accuracy: 0.6692

Epoch 692/900

355/355 [==============================] - 7s 19ms/step - loss: 9.9237 - accuracy: 0.7159 - val_loss: 0.6013 - val_accuracy: 0.6686

Epoch 693/900

355/355 [==============================] - 7s 19ms/step - loss: 9.8908 - accuracy: 0.7157 - val_loss: 0.6060 - val_accuracy: 0.6622

Epoch 694/900

355/355 [==============================] - 7s 19ms/step - loss: 9.9567 - accuracy: 0.7125 - val_loss: 0.6015 - val_accuracy: 0.6676

Epoch 695/900

355/355 [==============================] - 7s 19ms/step - loss: 9.9486 - accuracy: 0.7121 - val_loss: 0.5995 - val_accuracy: 0.6638

Epoch 696/900

355/355 [==============================] - 7s 19ms/step - loss: 10.1135 - accuracy: 0.7044 - val_loss: 0.6071 - val_accuracy: 0.6649

Epoch 697/900

355/355 [==============================] - 7s 19ms/step - loss: 9.9932 - accuracy: 0.7073 - val_loss: 0.6052 - val_accuracy: 0.6697

Epoch 698/900

355/355 [==============================] - 7s 19ms/step - loss: 9.9543 - accuracy: 0.7122 - val_loss: 0.6013 - val_accuracy: 0.6654

Epoch 699/900

355/355 [==============================] - 7s 19ms/step - loss: 9.9926 - accuracy: 0.7113 - val_loss: 0.6004 - val_accuracy: 0.6676

Epoch 700/900

355/355 [==============================] - 7s 19ms/step - loss: 9.9549 - accuracy: 0.7130 - val_loss: 0.5989 - val_accuracy: 0.6654

Epoch 701/900

355/355 [==============================] - 7s 19ms/step - loss: 9.9622 - accuracy: 0.7144 - val_loss: 0.6034 - val_accuracy: 0.6676

Epoch 702/900

355/355 [==============================] - 7s 19ms/step - loss: 10.0471 - accuracy: 0.7105 - val_loss: 0.5975 - val_accuracy: 0.6697

Epoch 703/900

355/355 [==============================] - 7s 19ms/step - loss: 9.8493 - accuracy: 0.7167 - val_loss: 0.6068 - val_accuracy: 0.6638

Epoch 704/900

355/355 [==============================] - 7s 19ms/step - loss: 9.9792 - accuracy: 0.7139 - val_loss: 0.6044 - val_accuracy: 0.6686

Epoch 705/900

355/355 [==============================] - 7s 19ms/step - loss: 9.9479 - accuracy: 0.7124 - val_loss: 0.6119 - val_accuracy: 0.6649

Epoch 706/900

355/355 [==============================] - 7s 19ms/step - loss: 9.9689 - accuracy: 0.7109 - val_loss: 0.6043 - val_accuracy: 0.6676

Epoch 707/900

355/355 [==============================] - 7s 19ms/step - loss: 9.9419 - accuracy: 0.7104 - val_loss: 0.5978 - val_accuracy: 0.6633

Epoch 708/900

355/355 [==============================] - 7s 19ms/step - loss: 9.8153 - accuracy: 0.7202 - val_loss: 0.6103 - val_accuracy: 0.6606

Epoch 709/900

355/355 [==============================] - 7s 19ms/step - loss: 9.9617 - accuracy: 0.7107 - val_loss: 0.6063 - val_accuracy: 0.6670

Epoch 710/900

355/355 [==============================] - 7s 19ms/step - loss: 9.8619 - accuracy: 0.7187 - val_loss: 0.6084 - val_accuracy: 0.6665

Epoch 711/900

355/355 [==============================] - 7s 19ms/step - loss: 9.9778 - accuracy: 0.7099 - val_loss: 0.5963 - val_accuracy: 0.6633

Epoch 712/900

355/355 [==============================] - 7s 19ms/step - loss: 9.8791 - accuracy: 0.7141 - val_loss: 0.6010 - val_accuracy: 0.6670

Epoch 713/900

355/355 [==============================] - 7s 19ms/step - loss: 9.8930 - accuracy: 0.7172 - val_loss: 0.6018 - val_accuracy: 0.6670

Epoch 714/900

355/355 [==============================] - 7s 19ms/step - loss: 9.8941 - accuracy: 0.7171 - val_loss: 0.6004 - val_accuracy: 0.6697

Epoch 715/900

355/355 [==============================] - 7s 19ms/step - loss: 9.8908 - accuracy: 0.7163 - val_loss: 0.6136 - val_accuracy: 0.6686

Epoch 716/900

355/355 [==============================] - 7s 19ms/step - loss: 9.9874 - accuracy: 0.7125 - val_loss: 0.6010 - val_accuracy: 0.6686

Epoch 717/900

355/355 [==============================] - 7s 19ms/step - loss: 9.9207 - accuracy: 0.7158 - val_loss: 0.6046 - val_accuracy: 0.6697

Epoch 718/900

355/355 [==============================] - 7s 19ms/step - loss: 9.8810 - accuracy: 0.7159 - val_loss: 0.6056 - val_accuracy: 0.6676

Epoch 719/900

355/355 [==============================] - 7s 19ms/step - loss: 9.9755 - accuracy: 0.7129 - val_loss: 0.5980 - val_accuracy: 0.6665

Epoch 720/900

355/355 [==============================] - 7s 19ms/step - loss: 9.8825 - accuracy: 0.7142 - val_loss: 0.6093 - val_accuracy: 0.6633

Epoch 721/900

355/355 [==============================] - 7s 19ms/step - loss: 9.8688 - accuracy: 0.7193 - val_loss: 0.6000 - val_accuracy: 0.6708

Epoch 722/900

355/355 [==============================] - 7s 19ms/step - loss: 9.8647 - accuracy: 0.7196 - val_loss: 0.6061 - val_accuracy: 0.6617

Epoch 723/900

355/355 [==============================] - 7s 19ms/step - loss: 9.8982 - accuracy: 0.7130 - val_loss: 0.6029 - val_accuracy: 0.6676

Epoch 724/900

355/355 [==============================] - 7s 19ms/step - loss: 9.8667 - accuracy: 0.7175 - val_loss: 0.6039 - val_accuracy: 0.6665

Epoch 725/900

355/355 [==============================] - 7s 19ms/step - loss: 9.9267 - accuracy: 0.7126 - val_loss: 0.6027 - val_accuracy: 0.6708

Epoch 726/900

355/355 [==============================] - 7s 19ms/step - loss: 9.8056 - accuracy: 0.7195 - val_loss: 0.6040 - val_accuracy: 0.6681

Epoch 727/900

355/355 [==============================] - 7s 19ms/step - loss: 9.8916 - accuracy: 0.7178 - val_loss: 0.6080 - val_accuracy: 0.6676

Epoch 728/900

355/355 [==============================] - 7s 19ms/step - loss: 9.8463 - accuracy: 0.7193 - val_loss: 0.6030 - val_accuracy: 0.6649

Epoch 729/900

355/355 [==============================] - 7s 19ms/step - loss: 9.9100 - accuracy: 0.7120 - val_loss: 0.5962 - val_accuracy: 0.6627

Epoch 730/900

355/355 [==============================] - 7s 19ms/step - loss: 9.8801 - accuracy: 0.7167 - val_loss: 0.5978 - val_accuracy: 0.6670

Epoch 731/900

355/355 [==============================] - 7s 19ms/step - loss: 9.7751 - accuracy: 0.7207 - val_loss: 0.6049 - val_accuracy: 0.6676

Epoch 732/900

355/355 [==============================] - 7s 19ms/step - loss: 9.8823 - accuracy: 0.7176 - val_loss: 0.5989 - val_accuracy: 0.6643

Epoch 00732: early stopping

110/110 [==============================] - 0s 3ms/step - loss: 0.9721 - accuracy: 0.5622
